# Global, Regional, and National Burden of Chronic Myeloid Leukemia, 1990–2017: A Systematic Analysis for the Global Burden of Disease Study 2017

**DOI:** 10.3389/fonc.2020.580759

**Published:** 2020-12-15

**Authors:** Qingqing Lin, Liping Mao, Li Shao, Li Zhu, Qingmei Han, Honghu Zhu, Jie Jin, Liangshun You

**Affiliations:** ^1^ Department of Hematology, The First Affiliated Hospital, College of Medicine, Zhejiang University, Hangzhou, China; ^2^ Malignant Lymphoma Diagnosis and Therapy Center, The First Affiliated Hospital, College of Medicine, Zhejiang University, Hangzhou, China; ^3^ Department of Pathology, Zhejiang University, Hangzhou, China

**Keywords:** chronic myeloid leukemia, epidemiology, incidence, death, disability-adjusted life-years

## Abstract

**Background:**

With the advent of tyrosine kinase inhibitors (TKIs), the prognosis of chronic myeloid leukemia (CML) seems to have dramatically improved over the last two decades. Accurate information of the global burden of CML is critical for direct health policy and healthcare resource allocation in the era of high-cost TKI therapy.

**Objective:**

This study aimed to evaluate the health burden of CML at global, regional, and national levels from 1990 to 2017.

**Methods:**

We collected data of CML between 1990 and 2017 from the Global Burden of Disease (GBD) study 2017 including, annual incidence, disease-related mortality, and disability-adjusted life-years (DALY), and the corresponding age-standardized rates (ASRs). To summarize the results, countries were categorized by sociodemographic index (SDI) quintiles and 21 GBD regions.

**Results:**

In 2017, an estimated 34,179 [95% Uncertainty Interval (UI), 31,516–36,714) incident cases of CML were recorded, and 24,054 (95%UI, 22,233–26,072) CML-related deaths were reported worldwide. Both, the age-standardized incidence rate (ASIR) and age-standardized death rate (ASDR) steadily decreased from 1990 to 2017, with estimated annual percentage changes (EAPCs) of −2.39 (95%UI, −8.13–3.71) and −2.74 (95%UI, −9.31–4.31), respectively. The global incidence and mortality of CML in males were higher than that in females. The ASRs varied substantially across regions, with the highest burden in Andean Latin America, Central Sub-Saharan Africa, and Southeast Asia. Besides, the ASRs decreased most obviously in the high-SDI regions compared to non-high-SDI regions. Moreover, the lower the SDI, the higher was the proportion of deaths in the younger age groups.

**Conclusion:**

Despite the decreasing trends of ASRs of CML from 1990 to 2017, the health-related burden of CML remains a challenge for the low-SDI regions. These findings highlight that appropriate strategies should be adopted in low-SDI countries to reduce the ASRs of CML.

## Introduction

Chronic myeloid leukemia (CML) is a clonal hematopoietic stem cell disorder and accounts for approximately 30% of the incidence of adult leukemias (1). Life expectancy for patients with CML has substantially improved since the advent of tyrosine kinase inhibitors (TKIs) at the turn of the century (2). Of note, the prognosis of CML has changed from a fatal disease to a disorder that is compatible with a normal lifespan (3). As a result, the prevalence of CML has increased dramatically, and may reach a plateau in 2050s (4). However, the high-cost TKI treatment combined with the rising prevalence of CML has led to a high global burden of CML treatment. Therefore, comparable epidemiological statistics such as age-standardized rates (ASRs) are important metrics to assess the global burden of CML in different countries at various levels of economic development, which may show potentially distinct patterns that can direct health policy and health care resource allocation in the era of high-cost TKI therapy.

At present, available data shows that the CML incidence distribution varies with age, gender, and regions (1, 5). The annual incidence of CML varies from 0.4/100,000 persons to 1.75/100,000 persons in different countries (6–8). The incidence of CML increases with age and is rare in children less than 14 years of age (0.7/million children/year) (9). Besides, CML is more common in males than in females with the male/female ratio of 1.2–1.7 (6, 10, 11). However, to the best of our knowledge, no studies have characterized the distribution and burden of CML worldwide.

The Global Burden of Disease (GBD) study from the Global Health Data Exchange (GHDx) assessed the burden of 354 diseases and injuries in 21 GBD regions and 194 countries worldwide. In this study, we collected data of CML between 1990 and 2017 from the GBD 2017 study, including incidence, disease-related mortality, disability adjusted life-years (DALYs), and the corresponding ASRs across gender, age, socio-demographic index (SDI), region, and country. Next, estimated annual percentage changes (EAPCs) were calculated to quantify the trends of ASRs. This was aimed to assess the accurate assessment of the distribution, burden, and trends of CML in different regions and countries that may provide directions to allocate limited resources and formulate more rational policies.

## Methods

### Data Source and Collection

All GBD 2017 data resources were available online from the GHDx query tool (http://ghdx.healthdata.org/gbd-results-tool). Regarding CML, we collected the information on incidence rate, death rate, and DALYs that was sorted by age, gender, and location from 1990 to 2017. The sociodemographic index (SDI), which was measured by the geometric average of total income per capita, educational attainment, and fertility, was used to divide the 194 countries into high-SDI, high-middle-SDI, middle-SDI, low-middle-SDI, and low-SDI countries. Based on a geographical hierarchy, GBD 2017 grouped the 194 countries into 21 regions. Therefore, the differences of CML burden and trends across these countries and regions were analyzed (12–14).

### Statistical Analysis

The annual age-standardized incidence rate (ASIR), age standardized death rate (ASDR), age-standardized DALY rate, and the corresponding estimated annual percentage changes (EAPCs) were calculated to assess the burden of CML prevalence. To assess the disease burden, the index of DALYs, which was estimated by addition of the YLDs and the years of life lost (YLLs), was applied. To assess the effect of the development status on the CML burden, we analyzed the association between SDI and prevalence indexes. The 95% Uncertainty Interval (UI) for each quantity was estimated in the analyses. The EAPCs were used to evaluate the trends of ASRs, which were calculated using a regression model: y = *α* + *β*x + *ϵ*, where y = ln (rate), x = calendar year, and *ϵ* = error term. EAPC = 100 × (exp^(^ *^β^* ^)^ − 1) and its 95%UI was obtained from the regression model (13, 15). If the EAPC and lower limit of UI were negative values, the incidence rate was considered to have a descending trend; in contrast, if the EAPC and upper limit of UI were positive, the incidence rate was considered to have an ascending trend. All calculations and analysis were performed using the R software (version 3.6.3). All tests were two-tailed, and a *P*-value of <0.05 was considered statistically significant.

## Results

### Temporal Trends on Incidence of CML

**Figure 1:**
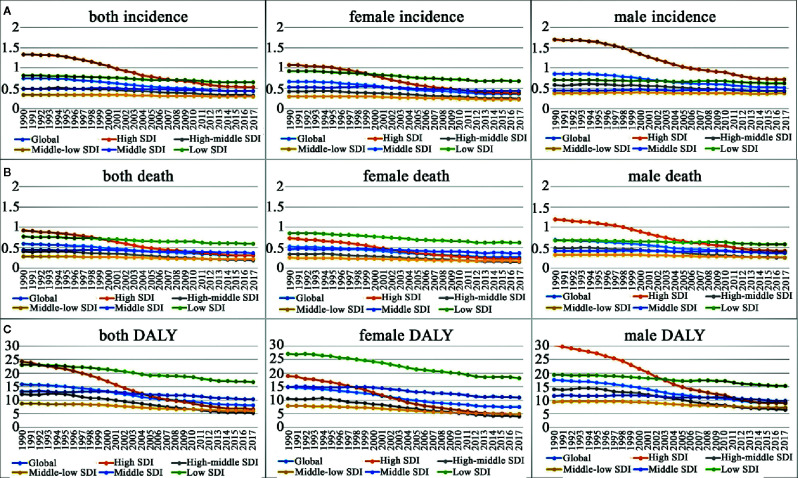
Change trends in age-standardized incidence, death and DALY rate by gender in different SDI quintiles from 1990 to 2017. **(A)** Age-standardized incidence rate; **(B)** age-standardized death rate; **(C)** age-standardized DALY rate. SDI, socio-demographic index.

**Figure 2:**
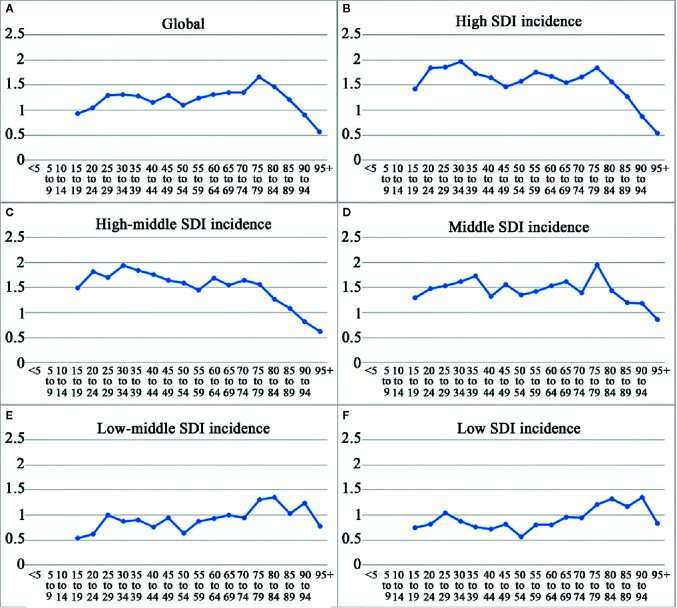
The ratio of male to female by age groups in different SDI quintiles in 2017. **(A)** Global; **(B)** high SDI; **(C)** high-middle SDI; **(D)** middle SDI; **(E)** low-middle SDI; **(F)** low SDI.

**Figure 3:**
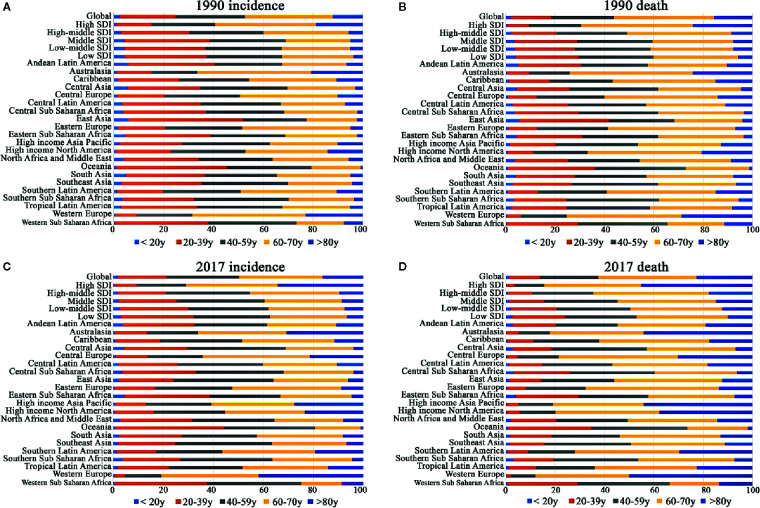
Proportion of age groups on incidence and death by region in 1990 and 2017. **(A)** incidence in 1990; **(B)** incidence in 2017; **(C)** death in 1990; **(D)** death in 2017.

**Figure 4:**
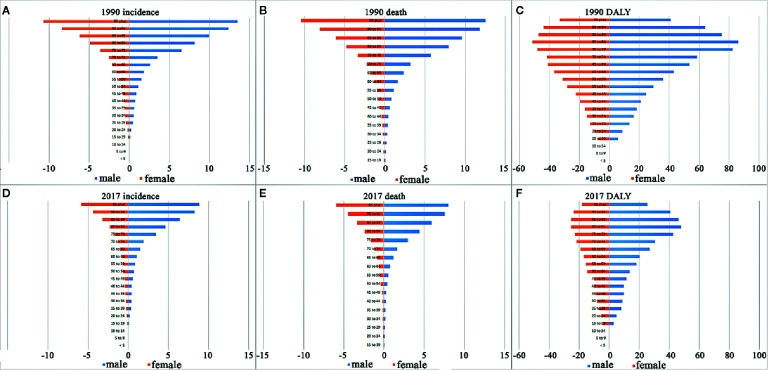
The incidence, death, and DALY rates by gender and age groups in 1990 and 2017. **(A)** Incidence rate in 1990; **(B)** death rate in 1990; **(C)** DALY rate in 1990; **(D)** incidence rate in 2017; **(E)** death rate in 2017; **(F)** DALY rate in 2017.

Globally, from 1990 to 2017, the number of annual incident cases of CML was stable, and there were 31,752 (95%UI, 29,590–34,066) cases in 1990 and 34,179 (95%UI, 31,516–36,714) cases in 2017 (**Table 1** **),** with a total increase of 7.64%. Contrarily, the ASIR decreased steadily, from 0.75 per 100,000 persons (95%UI, 0.71–0.8) in 1990 to 0.43 per 100,000 persons (95%UI, 0.4–0.46) in 2017. The EAPC (−2.39, 95%UI, −8.13–3.71) was negative, pointing to an obviously descending trend in the global ASIR over the past 28 years, especially since 1997 (**Table 1**, **Figure 1A**, **Supplementary Table S1**). CML occurred almost exclusively after the age of 20 years, and the incidence increased with age, both in males and females (**Table 1**, **Figures 2A**, **3A, C**, **4A, D**, **Supplementary Tables S1**, **S4**, **S5**, **S7**, **S9**, **S10**). However, the Global ASIR in males was higher than that in females over the past 28 years [0.52 (0.47–0.57) *vs.* 0.36 (0.31–0.41), respectively, in 2017] (**Table 1**, **Supplementary Table S1**). The ratio of the incidence between males and females showed a peak in the age group of 75-80 years, and dropped sharply thereafter (**Figure 2A**, **Supplementary Table S4**).

**Table 1:** Incident cases and ASIR in 1990 and 2017 and its change trends from 1990 to 2017.

	1990		2017		1990–2017 EAPC No. (95% CI)
	Incident casesNo. *10^2^(95% UI)	ASIR Per 100,000 No. (95% UI)	Incident cases No. *10^2^(95% UI)	ASIR Per 100,000 No. (95% UI)	
**Overall**	317.52 (295.9-340.66)	0.75 (0.71-0.8)	341.79 (315.16-367.14)	0.43 (0.4-0.46)	-2.39 ( -8.13 - 3.71)
**Sex**					
Female	151.96 (135.82-171.91)	0.67 (0.61-0.75)	150.33 (130.96-170.43)	0.36 (0.31-0.41)	-2.69 (-8.84 - 3.87)
Male	165.57 (153.46-177.23)	0.86 (0.8-0.92)	191.45 (173.2-208.22)	0.52 (0.47-0.57)	-2.16 (-7.47 - 3.45)
**Socio-demographic index**					
High SDI	163.42 (157.15-169.51)	1.34 (1.29-1.38)	106.4 (100.95-112.18)	0.53 (0.51-0.56)	-3.96 (-8.61 - 0.93)
High-middle SDI	49.58 (43.76-55.26)	0.49 (0.43-0.54)	57.35 (52.42-62.39)	0.33 (0.3-0.36)	-1.72 (-8.44 - 5.46)
Middle SDI	38.31 (34.17-44.49)	0.33 (0.3-0.39)	67.71 (58.04-72.69)	0.30 (0.26-0.32)	-0.55 (-8.25 - 7.80)
Low-middle SDI	32.8 (28.42-41.59)	0.48 (0.42-0.61)	56.67 (49.19-65.98)	0.43 (0.37-0.5)	-0.47 (-6.90 - 6.40)
Low SDI	32.8 (25.9-41.69)	0.81 (0.67-1.01)	52.85 (44.44-59.95)	0.65 (0.55-0.74)	-0.92 (-6.10 - 4.53)
**Region**					
Andean Latin America	0.87 (0.73-1)	0.34 (0.28-0.38)	2.08 (1.69-2.39)	0.36 (0.29-0.42)	0.45 (-6.92 - 8.42)
Australasia	3.87 (3.36-4.44)	1.64 (1.43-1.87)	3.58 (2.95-4.32)	0.83 (0.68-1)	-3.24 (-7.18 - 0.86)
Caribbean	2.64 (2.41-2.91)	0.93 (0.85-1.02)	3.07 (2.73-3.48)	0.61 (0.54-0.69)	-1.74 (-6.87 - 3.69)
Central Asia	2.52 (2.16-2.88)	0.45 (0.39-0.51)	2.74 (2.41-3.07)	0.32 (0.28-0.36)	-1.29 (-8.10 - 6.02)
Central Europe	11.11 (10.48-11.77)	0.77 (0.73-0.82)	6.82 (6.42-7.27)	0.37 (0.35-0.4)	-2.74 (-8.41 - 3.28)
Central Latin America	6.98 (6.69-7.4)	0.66 (0.64-0.7)	11.96 (11.22-12.85)	0.49 (0.46-0.53)	-1.42 (-7.05 - 4.55)
Central Sub-Saharan Africa	1.49 (0.99-1.92)	0.51 (0.35-0.64)	3.61 (2.64-4.51)	0.57 (0.42-0.72)	0.58 (-5.43 - 6.97)
East Asia	22.64 (17.43-28.02)	0.19 (0.16-0.24)	32.71 (27.97-37.64)	0.17 (0.15-0.19)	-0.99 (-11.04 - 10.19)
Eastern Europe	18.01 (15.98-20.72)	0.66 (0.59-0.75)	18.51 (16.46-20.67)	0.61 (0.54-0.69)	-0.54 (-5.89 - 5.11)
Eastern Sub-Saharan Africa	11.1 (8.15-15.09)	1.11 (0.86-1.49)	16.39 (12.56-19.89)	0.81 (0.63-0.98)	-1.41 (-5.88 - 3.29)
High-income Asia Pacific	17.08 (15.68-18.57)	0.87 (0.79-0.94)	12.81 (11.33-14.46)	0.39 (0.34-0.44)	-3.12 (-8.69 - 2.79)
High-income North America	34.1 (32.98-35.37)	1.02 (0.98-1.06)	20.55 (19.44-21.81)	0.4 (0.37-0.43)	-4.30 (-9.68 - 1.39)
North Africa and Middle East	11.85 (8.69-14.29)	0.57 (0.42-0.69)	18.32 (15.18-21.5)	0.38 (0.32-0.44)	-1.58 ( -7.91 - 5.19)
Oceania	0.33 (0.25-0.44)	0.78 (0.6-1)	0.55 (0.41-0.78)	0.59 (0.45-0.79)	-1.06 (-6.43 - 4.61)
South Asia	44.97 (38.05-55.49)	0.64 (0.55-0.79)	80.44 (69.27-91.61)	0.56 (0.48-0.63)	-0.58 (-6.27 - 5.46)
Southeast Asia	12.41 (10.18-17.69)	0.39 (0.33-0.57)	25.17 (19.85-29.4)	0.4 (0.31-0.46)	0.046 (-6.90 - 7.51)
Southern Latin America	3.77 (3.47-4.09)	0.8 (0.73-0.87)	3.0 (2.69-3.35)	0.38 (0.34-0.43)	-3.12 (-8.72 - 2.84)
Southern Sub-Saharan Africa	0.33 (0.25-0.42)	0.1 (0.07-0.12)	0.48 (0.36-0.6)	0.08 (0.06-0.09)	-0.69 (-14.17 - 14.90)
Tropical Latin America	7.2 (6.88-7.55)	0.65 (0.62-0.68)	7.65 (7.26-8.15)	0.33 (0.31-0.35)	-2.76 (-8.85 - 3.75)
Western Europe	99.5 (94.04-104.66)	1.78 (1.69-1.87)	61.62 (56.85-66.77)	0.69 (0.64-0.75)	-4.06 (-8.11 - 0.170)
Western Sub-Saharan Africa	4.74 (3.75-5.92)	0.43 (0.34-0.54)	9.72 (7.87-12.33)	0.42 (0.33-0.52)	-0.13 (-6.91 - 7.14)

ASIR, age-standardized incidence rate; EAPC, estimated annual percentage change.

The SDI-based regional analysis showed that the CML incidence was the highest in the high-SDI regions in 1990 and decreased steadily every year, especially after 1997. As shown in **Table 1**, the incident cases of CML in high-SDI regions were 16,342 (95%UI, 15,715–16951) in 1990, while they were 10,640 (95%UI, 10,095–11,218) in 2017, which were consistent with the trend of ASIR. Conversely, in the non-high-SDI regions, CML incident cases increased, whereas ASIR decreased. In addition, we also found a trend that the higher the SDI, the higher were the male/female ratio of incidence rates (**Figure 2**, **Supplementary Table S4**). It was remarkable that the proportion of younger (<60-years-old) incident cases in the high-SDI region decreased each year from 1990 to 2017, which was lower than that in non-high-SDI regions in 2017 (**Figure 3**, **Supplementary Table S5**, **S7**).

**Figure 5:**
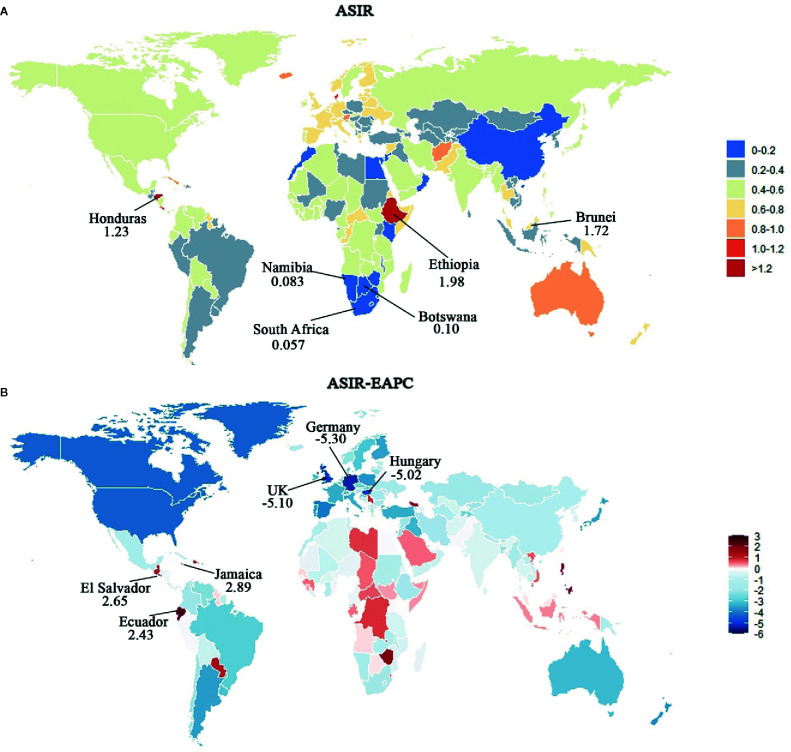
Map of ASIR and corresponding EAPCs by country in 2017 and their relative change trends from 1990 to 2017. **(A)** ASIR in 2017. Heat gradient represents ASIR from red (highest) to blue (lowest). Top three and bottom three countries with ASIR were annotated with numbers. **(B)** EAPCs of ASIR from 1990 to 2017. Heat gradient represents the change trends of EAPCs from red (highest) to blue (lowest). Blue indicates a downward trend and Red indicates an upward trend. Top three and bottom three countries with EAPCs were annotated with numbers.

While analyzing data for the 21 GBD regions, ASIR showed an upward trend in 3 GBD regions: Andean Latin America, Central Sub-Saharan Africa, and Southeast Asia, and a downward trend in the other 18 GBD regions. A significant decline of EAPC was observed in eight regions: Australasia, Central Europe, High-income Asia Pacific, High-income North America, Southern Latin America, Tropical Latin America, and Western Europe. Interestingly, compared with other regions, the proportion of younger patients (<60-years-old) in these eight regions decreased most significantly. The ASIR in 2017 and trends of ASIR over 28 years in 194 countries are presented in **Figure 5A**, of which, six countries with the ASIR more than one in 2017 were: Ethiopia, Brunei, Honduras, Seychelles, Denmark, and Costa Rica, in descending order. The five countries with the highest EAPC were Jamaica, El Salvador, Ecuador, Philippines, and Zimbabwe, while the five countries with the lowest EAPC were Germany, UK, Hungary, Israel, and Puerto Rico (details are available in **Supplementary Table S11**).

### Trends on Deaths Attributable to CML

The deaths attributable to CML were stable over the past 28 years with 24,198 (95%UI, 22,632–26,108) deaths in 1990 and 24,054 (95%UI, 22,233–26,072) deaths in 2017, while the ASDR decreased significantly with an EAPC −2.74 (95%UI, −9.31–4.31), dropping from 0.59/100,000 persons (95%UI, 0.56–0.63) to 0.31/100,000 persons (95%UI, 0.28–0.33) between 1990 and 2017 (**Table 2**). Consistent with the changes of ASIR, the ASDR increased with age in both sexes. However, ASDR in males was higher than that in females over the past 28 years (**Figure 1B**, **Supplementary Table S2**).

**Table 2:** Death cases and ASDR in 1990 and 2017 and its change trends from 1990 to 2017.

	1990		2017		1990–2017 EAPC No. (95% CI)
	Death cases No. *10^2^(95% UI)	ASDR Per 100,000No. (95% UI)	Death cases No. *10^2^(95% UI)	ASDRPer 100,000 No. (95% UI)	
**Overall**	734.92(666.26-806.31)	15.96(14.6-17.44)	240.54(222.33-260.72)	0.31(0.28-0.33)	-2.74(-9.31-4.31)
**Sex**					
Female	351.99(299.2-414.08)	14.78(17.36-12.72)	111.39(96.83-127.32)	0.26(0.23-0.3)	-2.88(-9.9-4.69)
Male	382.94(349.15-415.35)	17.48(18.92-16.08)	129.15(116.44-140.91)	0.36(0.33-0.39)	-2.63(-8.7-3.84)
**Socio-demographic index**					
High SDI	286.21(278.31-294.78)	24.23(24.96-23.53)	70.66(67.96-73.85)	0.31(0.3-0.32)	-4.51(-10.31-1.65)
High-middle SDI	129.2(112.36-144.95)	12.17(13.57-10.67)	33.92(31.8-37.07)	0.2(0.18-0.21)	-2.94(-10.73-5.54)
Middle SDI	110.8(96.98-129.21)	8.68(10.09-7.68)	45.79(39.63-49.07)	0.21(0.18-0.23)	-1.28(-9.92-8.2)
Low-middle SDI	100.72(86-128.82)	13.25(16.8-11.49)	45.12(39.06-52.46)	0.37(0.32-0.43)	-0.7(-7.51-6.6)
Low SDI	106.41(82.75-137.73)	23.14(29.19-18.45)	44.67(37.71-50.65)	0.6(0.5-0.68)	-1.03(-6.37-4.63)
**Region**					
Andean Latin America	2.58(2.16-2.98)	9.01(10.28-7.53)	1.46(1.19-1.64)	0.26(0.21-0.29)	-0.28(-8.51-8.68)
Australasia	6.8(6.49-7.13)	29.19(30.62-27.86)	2.13(1.89-2.43)	0.44(0.39-0.5)	-4.52(-9.53-0.77)
Caribbean	6.72(6.12-7.52)	22.56(25.15-20.54)	2.45(2.21-2.73)	0.48(0.44-0.54)	-2.08(-7.7-3.88)
Central Asia	7.39(6.31-8.47)	12.76(14.6-10.93)	1.98(1.75-2.2)	0.25(0.22-0.27)	-1.62(-9.21-6.61)
Central Europe	27.55(26.09-29.01)	19.12(20.16-18.14)	6.42(6.06-6.83)	0.31(0.29-0.33)	-2.98(-9.17-3.64)
Central Latin America	19.46(18.71-20.35)	16.88(17.64-16.22)	8.55(8.06-9.03)	0.36(0.34-0.38)	-1.97(-8.25-4.74)
Central Sub-Saharan Africa	4.86(3.14-6.32)	14.47(18.41-9.83)	3.02(2.21-3.75)	0.54(0.39-0.67)	0.55(-5.65-7.15)
East Asia	62.56(46.66-79.09)	5.02(6.27-3.85)	11.79(9.94-13.91)	0.06(0.05-0.07)	-3.49(-16.86-12.04)
Eastern Europe	44.34(40.1-50.69)	16.35(18.62-14.87)	12.37(11.71-13.09)	0.38(0.36-0.4)	-1.52(-7.72-5.1)
Eastern Sub-Saharan Africa	37.33(26.7-51.97)	32.79(43.98-24.4)	13.98(10.71-16.95)	0.77(0.59-0.94)	-1.4(-5.98-3.41)
High-income Asia Pacific	34.19(32.85-35.83)	17.16(18.02-16.47)	6.01(5.64-6.4)	0.14(0.13-0.15)	-5.14(-13.28-3.76)
High-income North America	73.89(71.98-76.1)	22.53(23.2-21.93)	15.6(14.97-16.43)	0.26(0.25-0.27)	-5.15(-11.56-1.71)
North Africa and Middle East	33.74(24.28-41.55)	14.58(17.84-10.63)	12.32(10.13-14.35)	0.28(0.23-0.32)	-2.25(-9.22-5.25)
Oceania	1.06(0.79-1.44)	23.03(30.17-17.52)	0.41(0.31-0.56)	0.47(0.37-0.62)	-1.28(-7.18-4.99)
South Asia	140.17(116.31-172.87)	17.35(21.55-14.7)	66.6(57.12-76.5)	0.49(0.43-0.57)	-0.8(-6.73-5.51)
Southeast Asia	36.28(28.98-52.09)	10.3(15-8.45)	16.51(13.01-19.91)	0.28(0.22-0.33)	-0.73(-8.48-7.69)
Southern Latin America	9.22(8.5-9.93)	19.28(20.78-17.79)	2.29(2.05-2.52)	0.28(0.25-0.31)	-3.66(-9.9-3.02)
Southern Sub-Saharan Africa	0.98(0.73-1.19)	2.63(3.24-1.96)	0.37(0.28-0.46)	0.06(0.05-0.08)	-0.79(-15.48-16.45)
Tropical Latin America	20.5(19.63-21.45)	16.99(17.74-16.31)	5.97(5.73-6.29)	0.26(0.25-0.28)	-3.06(-9.75-4.14)
Western Europe	150.45(144.73-156.46)	28.93(30.07-27.85)	42.45(40.25-44.87)	0.44(0.41-0.46)	-4.03(-9.01-1.22)
Western Sub-Saharan Africa	14.87(11.7-18.64)	12.23(15.37-9.58)	7.87(6.28-9.9)	0.37(0.3-0.47)	-0.21(-7.3-7.43)

ASDR, age-standardized death rate; EAPC, estimated annual percentage change.

The SDI-based analysis revealed that the ASDR in high- and high-middle-SDI regions decreased obviously each year, especially in the male subjects (**Figure 1B**, **Supplementary Table S2**). However, no significant changes were observed in the low- and middle-low-SDI regions. It was noteworthy that the low-SDI regions had the highest ASDR 0.6/100,000 persons (95%UI, 0.5–0.68) (**Table 2**, **Figure 1B**, **Supplementary Table S2**) in 2017. Moreover, the lower the SDI, the higher was the proportion of deaths in younger groups (<60-years-old) (**Figures 3B, D**, **Supplementary Table S6**, **S8**).

**Figure 6:**
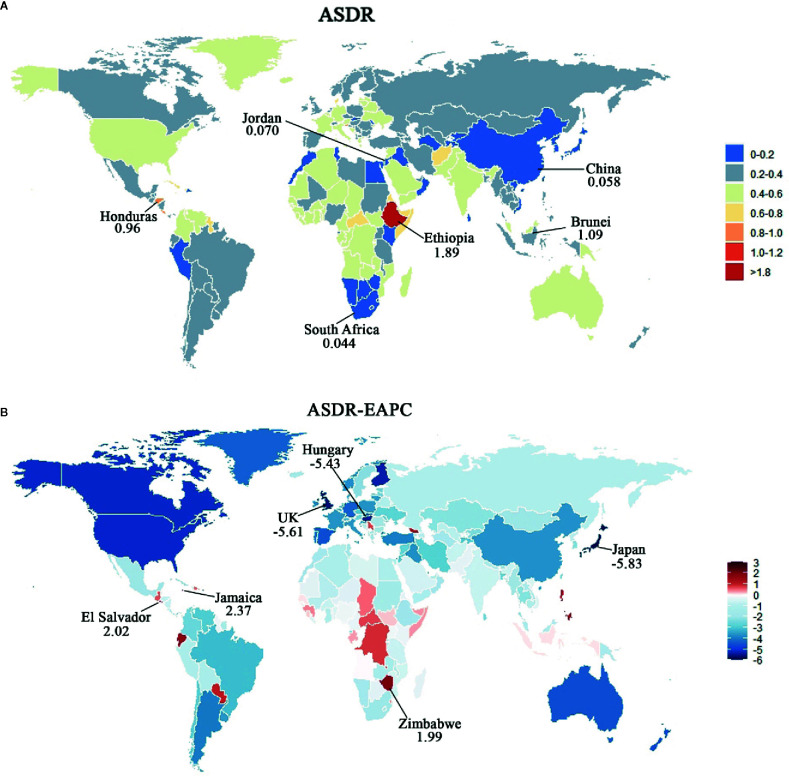
Map of ASDR and corresponding EAPCs by country in 2017 and their relative change trends from 1990 to 2017. **(A)** ASDR in 2017. Heat gradient represents ASIR from red (highest) to blue (lowest). Top three and bottom three countries with ASDR were annotated with numbers. **(B)** EAPCs of ASDR from 1990 to 2017. Heat gradient represents the change trends of EAPCs from red (highest) to blue (lowest). Blue indicates a downward trend and red indicates an upward trend. Top three and bottom three countries with EAPCs were annotated with numbers.

The analysis at the level of GBD regions revealed that the ASDR in all regions declined over past the 28 years, except for Central Sub-Saharan Africa (0.55, 95%UI, −5.65–7.15). Interestingly, the regions with the most obvious decrease of ASDR were the same as the regions with the most obvious decline of ASIR. Similarly, the top three countries in terms of ASDR were Ethiopia, Brunei, and Honduras, which also had the highest ASIR (**Figure 6A**, **Supplementary Table S12**). From 1990 to 2017, the ASDR declined in 168 countries (**Figure 6B**), of which nine countries with EAPC lower than −5 were Japan, Puerto Rico, UK, Hungary, Finland, Singapore, USA, Canada, and Bahrain. While, the seven countries with the highest EAPC (>1) were Jamaica, El Salvador, Zimbabwe, Ecuador, Lesotho, Philippines, and Georgia (details are available in **Supplementary Table S12**).

### Trends on DALYs Attributable to CML

The DALY attributable to CML decreased from 734,923 (95%UI, 666,259 to 806,312) in 1990 to 654,983 (95%UI, 594,726 to 712,947) in 2017. Simultaneously, the age-standardized DALY rate had fell significantly from 15.96 per 100,000 persons (95%UI, 14.60–17.44) to 8.17 per 100,000 persons (95%UI, 7.42–8.88) with an EAPC of −2.82 (95%UI, from −4.12 to −1.49). The Global DALY in males was higher than that in females over the past 28 years, [9 (95%UI, 8.01–9.9) *vs.* 7.48 (95%UI, 6.27–8.68), respectively, in 2017] (**Table 3**, **Figure 1C**, **Supplementary Table S3**). ASDR increased with age in both sexes and achieved the peak in 80–84-years-old age group (**Figures 4C, F**, **Supplementary Tables S3**, **S9**, **S10**).

**Table 3:** DALY and age-standardized DALY rate in 1990 and 2017 and its change trends from 1990 to 2017.

	1990		2017		1990–2017EAPCNo. (95% CI)
	DALY No. *10^3^(95% UI)	Age-standardized DALY ratePer 100,000 No. (95% UI)	DALYNo. *10^3^(95% UI)	Age-standardized DALY ratePer 100,000 No. (95% UI)	
**Overall**	317.52 (295.9-340.66)	0.75 (0.71-0.8)	654.98(594.73-712.95)	8.17(7.42-8.88)	-2.82(-4.12--1.49)
**Sex**					
Female	151.96 (135.82-171.91)	0.67 (0.61-0.75)	307.74(258.51-357.55)	7.48(6.27-8.68)	-2.88(-4.24--1.49)
Male	165.57 (153.46-177.23)	0.86 (0.8-0.92)	347.24(308.67-383.12)	9(8.01-9.9)	-2.77(-4.01--1.51)
**Socio-demographic index**					
High SDI	163.42 (157.15-169.51)	1.34 (1.29-1.38)	125.91(121.16-131.32)	6.81(6.57-7.08)	-5.25(-6.46--4.02)
High-middle SDI	49.58 (43.76-55.26)	0.49 (0.43-0.54)	91.51(85-100.26)	5.26(4.9-5.76)	-3.47(-4.97--1.94)
Middle SDI	38.31 (34.17-44.49)	0.33 (0.3-0.39)	137.8(120.1-148.24)	6.03(5.25-6.47)	-1.58(-3.23-0.09)
Low-middle SDI	32.8 (28.42-41.59)	0.48 (0.42-0.61)	146.73(127.78-171.91)	10.33(8.95-12.07)	-0.99(-2.28-0.32)
Low SDI	32.8 (25.9-41.69)	0.81 (0.67-1.01)	151.91(126.85-172.16)	16.71(14.03-18.84)	-1.34(-2.35--0.32)
**Region**					
Andean Latin America	0.87 (0.73-1)	0.34 (0.28-0.38)	4.49(3.61-5.14)	7.68(6.21-8.76)	-0.45(-2-1.13)
Australasia	3.87 (3.36-4.44)	1.64 (1.43-1.87)	4.02(3.54-4.58)	9.87(8.64-11.2)	-4.72(-5.78--3.65)
Caribbean	2.64 (2.41-2.91)	0.93 (0.85-1.02)	6.66(5.9-7.62)	13.28(11.77-15.18)	-2.16(-3.26--1.05)
Central Asia	2.52 (2.16-2.88)	0.45 (0.39-0.51)	6.69(5.93-7.44)	7.62(6.77-8.45)	-2(-3.37--0.62)
Central Europe	11.11 (10.48-11.77)	0.77 (0.73-0.82)	13.52(12.8-14.32)	7.53(7.14-7.98)	-3.65(-4.89--2.4)
Central Latin America	6.98 (6.69-7.4)	0.66 (0.64-0.7)	24.79(23.35-26.32)	9.98(9.41-10.58)	-2.28(-3.49--1.05)
Central Sub-Saharan Africa	1.49 (0.99-1.92)	0.51 (0.35-0.64)	11.01(7.99-13.7)	14.97(10.91-18.55)	0.26(-0.92-1.45)
East Asia	22.64 (17.43-28.02)	0.19 (0.16-0.24)	36.19(30.76-43.35)	1.9(1.63-2.27)	-4.29(-6.71--1.8)
Eastern Europe	18.01 (15.98-20.72)	0.66 (0.59-0.75)	32.48(30.66-34.41)	10.85(10.26-11.55)	-1.94(-3.1--0.77)
Eastern Sub-Saharan Africa	11.1 (8.15-15.09)	1.11 (0.86-1.49)	51.15(39.01-62.33)	21.72(16.64-26.33)	-1.81(-2.67--0.95)
High-income Asia Pacific	17.08 (15.68-18.57)	0.87 (0.79-0.94)	11.61(10.84-12.33)	3.85(3.59-4.12)	-5.94(-7.47--4.37)
High-income North America	34.1 (32.98-35.37)	1.02 (0.98-1.06)	31.36(29.89-33.21)	6.14(5.82-6.5)	-5.87(-7.17--4.55)
North Africa and Middle East	11.85 (8.69-14.29)	0.57 (0.42-0.69)	39.69(32.3-47.11)	7.66(6.27-9)	-2.47(-3.82--1.09)
Oceania	0.33 (0.25-0.44)	0.78 (0.6-1)	1.65(1.2-2.34)	16.36(12.26-22.41)	-1.3(-2.33--0.27)
South Asia	44.97 (38.05-55.49)	0.64 (0.55-0.79)	207.79(180.43-239.39)	13.25(11.44-15.19)	-1.06(-2.21-0.09)
Southeast Asia	12.41 (10.18-17.69)	0.39 (0.33-0.57)	52.2(41.39-62.2)	7.87(6.24-9.39)	-0.99(-2.46-0.5)
Southern Latin America	3.77 (3.47-4.09)	0.8 (0.73-0.87)	5.36(4.81-5.91)	7(6.27-7.73)	-4.08(-5.33--2.82)
Southern Sub-Saharan Africa	0.33 (0.25-0.42)	0.1 (0.07-0.12)	1.26(0.93-1.57)	1.88(1.39-2.3)	-1.03(-3.82-1.84)
Tropical Latin America	7.2 (6.88-7.55)	0.65 (0.62-0.68)	15.79(15.04-16.59)	6.64(6.33-6.97)	-3.68(-4.98--2.37)
Western Europe	99.5 (94.04-104.66)	1.78 (1.69-1.87)	68.46(64.8-72.73)	8.84(8.39-9.38)	-4.8(-5.87--3.72)
Western Sub-Saharan Africa	4.74 (3.75-5.92)	0.43 (0.34-0.54)	28.79(23.26-37.07)	10.82(8.63-13.73)	-0.51(-1.84-0.84)

DALY, disability-adjusted life year; EAPC, estimated annual percentage change.

**Figure 7:**
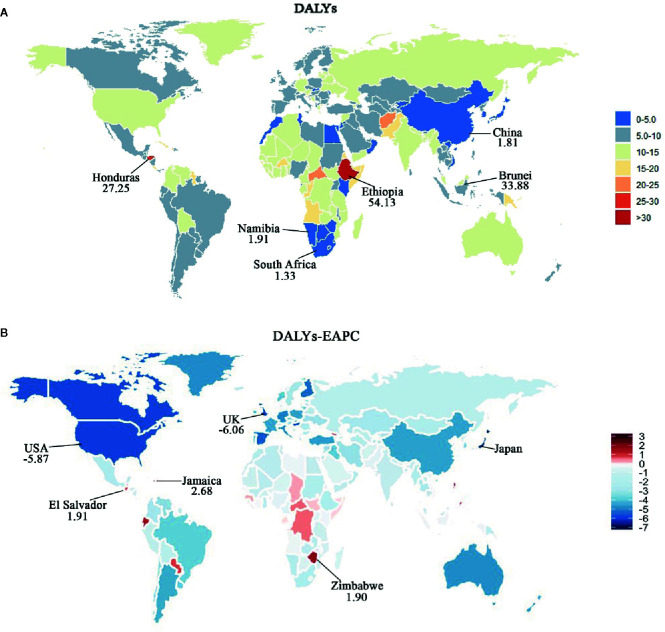
Map of age-standardized DALY rate and corresponding EAPCs by country in 2017 and their relative change trends from 1990 to 2017. **(A)** Age-standardized DALY rate in 2017. Heat gradient represents age-standardized DALY rate from red (highest) to blue (lowest). Top three and bottom three countries with age-standardized DALY rate were annotated with numbers. **(B)** EAPCs of age-standardized DALY rate from 1990 to 2017. Heat gradient represents the change trends of EAPCs from red (highest) to blue (lowest). Blue indicates a downward trend and Red indicates an upward trend. Top three and bottom three countries with EAPCs were annotated with numbers.

The age-standardized DALY rates declined in each SDI region over the past 28 years, especially since 1997. However, the decline was more pronounced in high- and high-middle-SDI regions (**Table 3**, **Figure 1C**, **Supplementary Table S3**). There was wide geographic variation in the age-standardized DALY rate of CML. Regions with an increase were only observed in Central Sub Saharan Africa. Ethiopia, one country of Central Sub Saharan Africa, had the largest age-standardized DALY over the past 28 years and that of 54.13 per 100,000 persons (95%UI, 38.02–69.90) in 2017 (**Table 3**, **Supplementary Table S13** **).** The top three countries of age-standardized DALY were Ethiopia, Brunei, and Honduras, which also had the highest ASIR and ASDR The seven countries with the highest EAPC (>1) of age-standardized DALY were the same which also had the highest ASDR (**Figures 7A, B**, **Supplementary Table S3**).

## Discussion

Our analysis based on the GBD study 2017 presented the latest global patterns and long-term trends of the incidence, mortality, and DALYs attributable to CML. Overall, the cases of incidence, deaths, and DALYs were stable between 1990 and 2017 globally. On the contrary, the ASIR, ASDR, and age-standardized DALY rates of CML showed decreasing trends. Especially since 1997, the year of approval of the first TKI, the decline of ASRs became more pronounced, pointing to the contribution of TKI treatment to the changes in the global patterns of CML. In addition, the burden of CML varied widely across different SDI-based regions. Our analysis showed that the age-adjusted incidence, mortality, and DALY rates of CML in the high- and high-middle-SDI regions decreased steadily over the past 28 years. However, in the low-SDI regions, these rates did not improve significantly, and they had the highest incidence, and mortality. We also revealed that the lower the SDI, the higher was the proportion of incidence and deaths in the younger age groups.

Previous studies have demonstrated that cancer survival rates between countries with different income levels differ for reasons such as variations in effective treatment, specialized health care, insurance status, and cancer prevention programs (5, 16, 17). We speculate that out of these factors, the most important explanation of our findings might be the lack of basic diagnostic and treatment options in the low-income countries that were well-established in the high-income countries, including the non-availability of TKI therapy or non-adherence to that (6). Besides, due to the lack of widely-available monitoring technology in the low-SDI regions, the efficacy of TKI treatment couldn’t be evaluated in time, which might be another important factor. These findings raise an alarm that rational allocation of existing resources is urgently needed to reduce the burden of CML in the low-SDI regions.

Consistent with previous findings, the incidence and mortality of CML varied across GBD regions and countries (6–8, 18–21). ASIR showed an upward trend in three GBD regions: Central Sub-Saharan Africa, Andean Latin America, and Southeast Asia. Moreover, Central Sub-Saharan Africa also showed an upward trend of ASDR, and age-standardized DALY, suggesting the highest burden of CML in this region. The geographic variations in the incidence of CML could partly be attributed to the methodological factors (6). In particular, the inclusion of referral patients in some registries might have resulted in an overestimation of the incidence of CML, whereas incomplete reporting of new CML cases might lead to a low incidence. Although these methodological issues cannot be ruled out as the cause of the divergence in CML incidence, the possibility of a real variation between different regions cannot be excluded. In fact, geographical differences of CML incidence have been shown in few previous studies. A report from the EUTOS registry (22), based on epidemiological data from 20 European countries between 2008 and 2012, showed that the CML incidence varied from 0.76 (UK) to 1.96 (Finland) per 100,000 persons. Another report from Chen Y. et al. (7, 23) suggested that the incidence of CML is lower in some Asian countries, which was consistent with our findings. Interestingly, similar to the regional patterns of Hodgkin’s Lymphoma (24), the countries with the highest incidence and mortality of CML were mostly coastal or island countries, such as Ethiopia, Brunei, Honduras, Seychelles, Denmark, and Costa Rica. The causes of this phenomenon remain to be elucidated and require further study.

CML is a clonal myeloproliferative neoplasm characterized by a reciprocal translocation between chromosomes 9 and 22. Although the pathogenesis is well known, the etiology of CML remains incompletely understood. A recent study based on the Swedish Cancer Registry showed no evidence that CML was heritable (25). To date, ionizing radiation is the only confirmed risk factor for CML (26). The association between smoking or benzene exposure and CML has also been reported in few studies, but this needs to be confirmed (26–29). The analysis of epidemiological characteristics of CML may be helpful for the exploration of its etiology. Incidence of CML is rare in children and increases with age, at least up to 75–80 years, suggesting that the aging may be a risk factor of CML. The ratio of male to female incidence showed a peak in 75–80 years age group and dropped sharply after 80 years old. These results demonstrated that the occurrence of CML was related to sex, and female-related hormones might be a protective factor of CML. Moreover, the countries with the highest incidence of CML were mostly coastal or island countries. In particular, the incidence of Ethiopia was significantly higher than other countries. In-depth investigation of the environment in these countries may reveal additional clues to the causes of CML.

This study inevitably suffers from some limitations. The accuracy of the results depended on the quality of GBD data. However, in some underdeveloped regions, because of limited specialized medical care and no reliable mortality information systems, the accuracy and integrity of the data cannot be guaranteed. Besides, it can’t be ignored that misdiagnosed patients might have a potential impact on the incident cases. Especially, in the 1990’s, because of the poor diagnostic techniques of molecular biology, the incidence rate of CML may be underestimated. However, the misdiagnosis didn’t affect the decline trend of CML incidence rate. What’s more, the GBD study is based on countries and regions; therefore, the influence of race could not be analyzed.

## Conclusion

In conclusion, based on the analysis of GHDx data, the overall incidence rates, deaths, and DALYs related to CML were stable between 1990 and 2017; however, the ASRs of CML showed a downward trend worldwide. Most of the CML burden was observed in males, especially among the older population. The low-SDI quintile had the heaviest burden of CML, especially in the regions of Andean Latin America, Central Sub-Saharan Africa, and Southeast Asia. Our analysis of the distribution, burden and trends of CML in different regions and countries may provide a direction to allocate limited resources and formulate more rational policies.

## Data Availability Statement

The original contributions presented in the study are included in the article/**Supplementary Materials**. Further inquiries can be directed to the corresponding authors.

## Author Contributions

JJ, HZ, and LY designed the study. LY and QL collected and analyzed data. LY wrote the main manuscript. QL, LY, LS, LM, QH, and LZ performed the figures and tables. JJ and HZ reviewed the manuscript. All authors contributed to the article and approved the submitted version.

## Funding

The research was supported by National Natural Science Foundation of China (No. 81873451).

## Conflict of Interest

The authors declare that the research was conducted in the absence of any commercial or financial relationships that could be construed as a potential conflict of interest.
