# Supplementary material for: Global, Regional, and National Burden of Chronic Myeloid Leukemia, 1990–2017: A Systematic Analysis for the Global Burden of Disease Study 2017
Source: Front Oncol. 2020 Dec 15;10:580759. doi: 10.3389/fonc.2020.580759 (PMC7770240; doi:10.3389/fonc.2020.580759)
Supplement: Supplementary file 1 [file Table_1.doc]

**Supplementary table S1. Age-standardized incidence rate by gender in different SDI quintiles from 1990 to 2017.**

| Year | Gender | Age-standardized incidence rate  per 100,000  No.(95% UI) | | | | | |
| --- | --- | --- | --- | --- | --- | --- | --- |
|  |  | Global | High SDI | High-middle SDI | Middle SDI | Low-middle SDI | Low SDI |
| 1990 | both | 0·75 (0·71-0·8) | 1·34 (1·29-1·38) | 0·49 (0·43-0·54) | 0·33 (0·3-0·39) | 0·48 (0·42-0·61) | 0·81 (0·67-1·01) |
| 1991 | both | 0·75 (0·7-0·79) | 1·33 (1·29-1·38) | 0·49 (0·43-0·54) | 0·33 (0·3-0·39) | 0·49 (0·43-0·61) | 0·81 (0·67-0·99) |
| 1992 | both | 0·74 (0·7-0·79) | 1·32 (1·28-1·36) | 0·49 (0·44-0·54) | 0·34 (0·31-0·39) | 0·49 (0·43-0·61) | 0·81 (0·67-0·99) |
| 1993 | both | 0·74 (0·7-0·79) | 1·32 (1·28-1·36) | 0·51 (0·45-0·55) | 0·34 (0·3-0·39) | 0·49 (0·43-0·61) | 0·81 (0·67-0·98) |
| 1994 | both | 0·74 (0·7-0·78) | 1·3 (1·26-1·34) | 0·51 (0·46-0·55) | 0·34 (0·3-0·39) | 0·48 (0·43-0·6) | 0·8 (0·66-0·97) |
| 1995 | both | 0·73 (0·69-0·77) | 1·28 (1·25-1·32) | 0·51 (0·45-0·55) | 0·34 (0·31-0·39) | 0·48 (0·43-0·6) | 0·8 (0·66-0·96) |
| 1996 | both | 0·71 (0·67-0·75) | 1·24 (1·21-1·28) | 0·49 (0·44-0·54) | 0·34 (0·31-0·39) | 0·48 (0·43-0·6) | 0·79 (0·66-0·95) |
| 1997 | both | 0·7 (0·65-0·74) | 1·2 (1·17-1·24) | 0·48 (0·43-0·52) | 0·34 (0·3-0·39) | 0·49 (0·43-0·61) | 0·79 (0·66-0·95) |
| 1998 | both | 0·68 (0·63-0·72) | 1·16 (1·13-1·2) | 0·47 (0·42-0·51) | 0·34 (0·3-0·39) | 0·5 (0·44-0·61) | 0·78 (0·65-0·93) |
| 1999 | both | 0·66 (0·62-0·7) | 1·1 (1·07-1·14) | 0·48 (0·42-0·51) | 0·34 (0·3-0·38) | 0·5 (0·44-0·6) | 0·77 (0·64-0·92) |
| 2000 | both | 0·64 (0·6-0·68) | 1·04 (1·01-1·08) | 0·47 (0·42-0·5) | 0·34 (0·3-0·38) | 0·5 (0·44-0·6) | 0·76 (0·63-0·91) |
| 2001 | both | 0·62 (0·58-0·66) | 0·98 (0·95-1·02) | 0·46 (0·41-0·49) | 0·34 (0·3-0·37) | 0·5 (0·43-0·59) | 0·75 (0·62-0·89) |
| 2002 | both | 0·6 (0·56-0·63) | 0·92 (0·89-0·96) | 0·45 (0·41-0·47) | 0·34 (0·3-0·37) | 0·49 (0·43-0·58) | 0·75 (0·61-0·88) |
| 2003 | both | 0·58 (0·54-0·62) | 0·88 (0·85-0·91) | 0·44 (0·4-0·46) | 0·33 (0·29-0·36) | 0·48 (0·42-0·57) | 0·74 (0·61-0·87) |
| 2004 | both | 0·56 (0·52-0·59) | 0·82 (0·79-0·85) | 0·43 (0·39-0·45) | 0·32 (0·29-0·36) | 0·47 (0·41-0·56) | 0·72 (0·59-0·85) |
| 2005 | both | 0·54 (0·5-0·57) | 0·78 (0·75-0·81) | 0·42 (0·39-0·45) | 0·32 (0·28-0·35) | 0·47 (0·41-0·55) | 0·71 (0·59-0·84) |
| 2006 | both | 0·52 (0·49-0·56) | 0·74 (0·72-0·77) | 0·4 (0·37-0·43) | 0·32 (0·28-0·35) | 0·47 (0·41-0·55) | 0·71 (0·58-0·83) |
| 2007 | both | 0·51 (0·48-0·54) | 0·72 (0·69-0·75) | 0·39 (0·36-0·42) | 0·31 (0·28-0·34) | 0·47 (0·42-0·54) | 0·71 (0·58-0·82) |
| 2008 | both | 0·51 (0·47-0·54) | 0·7 (0·67-0·72) | 0·39 (0·36-0·41) | 0·31 (0·27-0·34) | 0·47 (0·41-0·54) | 0·71 (0·59-0·82) |
| 2009 | both | 0·5 (0·46-0·53) | 0·68 (0·65-0·7) | 0·38 (0·35-0·41) | 0·31 (0·27-0·34) | 0·47 (0·41-0·54) | 0·7 (0·58-0·81) |
| 2010 | both | 0·49 (0·45-0·52) | 0·65 (0·63-0·68) | 0·38 (0·35-0·4) | 0·31 (0·27-0·34) | 0·47 (0·41-0·54) | 0·7 (0·58-0·8) |
| 2011 | both | 0·47 (0·43-0·5) | 0·62 (0·59-0·64) | 0·36 (0·33-0·38) | 0·31 (0·27-0·33) | 0·46 (0·4-0·53) | 0·68 (0·57-0·78) |
| 2012 | both | 0·46 (0·42-0·48) | 0·59 (0·56-0·61) | 0·35 (0·32-0·37) | 0·3 (0·26-0·33) | 0·45 (0·39-0·52) | 0·67 (0·56-0·76) |
| 2013 | both | 0·45 (0·41-0·47) | 0·56 (0·54-0·58) | 0·34 (0·32-0·37) | 0·3 (0·26-0·32) | 0·44 (0·39-0·51) | 0·66 (0·55-0·75) |
| 2014 | both | 0·44 (0·4-0·47) | 0·54 (0·52-0·57) | 0·34 (0·31-0·36) | 0·3 (0·26-0·32) | 0·44 (0·38-0·51) | 0·66 (0·55-0·75) |
| 2015 | both | 0·44 (0·4-0·47) | 0·54 (0·52-0·56) | 0·34 (0·31-0·37) | 0·3 (0·26-0·32) | 0·44 (0·38-0·5) | 0·66 (0·56-0·75) |
| 2016 | both | 0·44 (0·4-0·47) | 0·54 (0·51-0·56) | 0·34 (0·31-0·36) | 0·3 (0·26-0·32) | 0·43 (0·38-0·5) | 0·65 (0·55-0·74) |
| 2017 | both | 0·43 (0·4-0·46) | 0·53 (0·51-0·56) | 0·33 (0·3-0·36) | 0·3 (0·26-0·32) | 0·43 (0·37-0·5) | 0·65 (0·55-0·74) |
| 1990 | female | 0·67 (0·61-0·75) | 1·08 (1·01-1·14) | 0·42 (0·36-0·5) | 0·3 (0·25-0·4) | 0·52 (0·43-0·75) | 0·93 (0·71-1·32) |
| 1991 | female | 0·67 (0·6-0·75) | 1·07 (1·01-1·13) | 0·42 (0·35-0·5) | 0·3 (0·25-0·39) | 0·53 (0·43-0·75) | 0·92 (0·71-1·3) |
| 1992 | female | 0·66 (0·6-0·74) | 1·05 (1-1·11) | 0·42 (0·36-0·5) | 0·3 (0·25-0·39) | 0·53 (0·43-0·75) | 0·93 (0·71-1·3) |
| 1993 | female | 0·66 (0·59-0·74) | 1·05 (1-1·1) | 0·44 (0·37-0·5) | 0·3 (0·25-0·39) | 0·53 (0·43-0·74) | 0·92 (0·71-1·28) |
| 1994 | female | 0·65 (0·58-0·73) | 1·03 (0·98-1·08) | 0·44 (0·37-0·5) | 0·3 (0·25-0·38) | 0·52 (0·42-0·73) | 0·91 (0·71-1·27) |
| 1995 | female | 0·65 (0·58-0·72) | 1·02 (0·97-1·07) | 0·43 (0·37-0·49) | 0·3 (0·25-0·38) | 0·52 (0·42-0·73) | 0·9 (0·7-1·25) |
| 1996 | female | 0·63 (0·56-0·7) | 0·98 (0·93-1·02) | 0·42 (0·35-0·48) | 0·3 (0·24-0·38) | 0·52 (0·42-0·73) | 0·88 (0·68-1·23) |
| 1997 | female | 0·62 (0·54-0·69) | 0·95 (0·9-0·99) | 0·41 (0·34-0·46) | 0·3 (0·24-0·37) | 0·53 (0·43-0·74) | 0·88 (0·68-1·22) |
| 1998 | female | 0·6 (0·53-0·67) | 0·91 (0·86-0·95) | 0·4 (0·33-0·45) | 0·3 (0·24-0·37) | 0·54 (0·43-0·74) | 0·87 (0·68-1·2) |
| 1999 | female | 0·58 (0·51-0·66) | 0·86 (0·82-0·91) | 0·39 (0·34-0·44) | 0·3 (0·24-0·36) | 0·54 (0·43-0·74) | 0·86 (0·67-1·17) |
| 2000 | female | 0·56 (0·5-0·64) | 0·81 (0·77-0·85) | 0·39 (0·33-0·42) | 0·29 (0·24-0·35) | 0·54 (0·42-0·73) | 0·85 (0·65-1·15) |
| 2001 | female | 0·54 (0·48-0·61) | 0·76 (0·72-0·8) | 0·38 (0·33-0·41) | 0·29 (0·24-0·35) | 0·53 (0·42-0·72) | 0·83 (0·64-1·13) |
| 2002 | female | 0·52 (0·46-0·59) | 0·71 (0·67-0·75) | 0·37 (0·32-0·4) | 0·28 (0·23-0·34) | 0·52 (0·41-0·7) | 0·82 (0·63-1·1) |
| 2003 | female | 0·5 (0·45-0·56) | 0·67 (0·63-0·71) | 0·36 (0·32-0·39) | 0·28 (0·23-0·33) | 0·51 (0·41-0·67) | 0·8 (0·62-1·07) |
| 2004 | female | 0·48 (0·43-0·54) | 0·62 (0·58-0·66) | 0·35 (0·3-0·38) | 0·27 (0·23-0·32) | 0·5 (0·39-0·65) | 0·78 (0·61-1·03) |
| 2005 | female | 0·46 (0·41-0·53) | 0·58 (0·55-0·62) | 0·34 (0·3-0·37) | 0·27 (0·22-0·31) | 0·49 (0·39-0·65) | 0·76 (0·6-1) |
| 2006 | female | 0·45 (0·4-0·51) | 0·55 (0·52-0·58) | 0·32 (0·29-0·35) | 0·26 (0·22-0·3) | 0·49 (0·39-0·63) | 0·75 (0·59-0·99) |
| 2007 | female | 0·44 (0·39-0·49) | 0·53 (0·5-0·56) | 0·32 (0·28-0·34) | 0·25 (0·21-0·29) | 0·48 (0·38-0·61) | 0·74 (0·58-0·97) |
| 2008 | female | 0·43 (0·38-0·48) | 0·51 (0·48-0·54) | 0·31 (0·28-0·34) | 0·25 (0·21-0·29) | 0·47 (0·38-0·6) | 0·74 (0·57-0·96) |
| 2009 | female | 0·42 (0·37-0·47) | 0·49 (0·46-0·52) | 0·3 (0·27-0·33) | 0·25 (0·21-0·28) | 0·47 (0·37-0·59) | 0·73 (0·57-0·94) |
| 2010 | female | 0·41 (0·36-0·46) | 0·47 (0·44-0·5) | 0·29 (0·26-0·32) | 0·25 (0·2-0·28) | 0·47 (0·37-0·58) | 0·72 (0·56-0·93) |
| 2011 | female | 0·39 (0·34-0·44) | 0·44 (0·42-0·47) | 0·28 (0·25-0·3) | 0·24 (0·2-0·28) | 0·45 (0·36-0·57) | 0·7 (0·55-0·9) |
| 2012 | female | 0·38 (0·33-0·43) | 0·42 (0·4-0·45) | 0·27 (0·24-0·3) | 0·24 (0·2-0·27) | 0·44 (0·35-0·55) | 0·68 (0·54-0·87) |
| 2013 | female | 0·37 (0·33-0·42) | 0·4 (0·38-0·43) | 0·26 (0·24-0·29) | 0·24 (0·19-0·27) | 0·44 (0·35-0·54) | 0·68 (0·53-0·86) |
| 2014 | female | 0·37 (0·32-0·42) | 0·39 (0·37-0·42) | 0·26 (0·23-0·29) | 0·23 (0·19-0·27) | 0·44 (0·35-0·54) | 0·68 (0·53-0·86) |
| 2015 | female | 0·37 (0·32-0·42) | 0·39 (0·36-0·42) | 0·26 (0·23-0·29) | 0·23 (0·19-0·27) | 0·44 (0·34-0·54) | 0·69 (0·53-0·87) |
| 2016 | female | 0·36 (0·32-0·41) | 0·38 (0·36-0·41) | 0·25 (0·23-0·28) | 0·24 (0·19-0·27) | 0·44 (0·34-0·54) | 0·68 (0·52-0·86) |
| 2017 | female | 0·36 (0·31-0·41) | 0·38 (0·35-0·41) | 0·24 (0·22-0·28) | 0·23 (0·19-0·27) | 0·43 (0·34-0·53) | 0·68 (0·52-0·85) |
| 1990 | male | 0·86 (0·8-0·92) | 1·69 (1·62-1·77) | 0·58 (0·51-0·64) | 0·38 (0·33-0·44) | 0·45 (0·37-0·56) | 0·71 (0·53-0·88) |
| 1991 | male | 0·86 (0·8-0·91) | 1·69 (1·62-1·77) | 0·58 (0·51-0·63) | 0·38 (0·33-0·44) | 0·45 (0·37-0·56) | 0·7 (0·53-0·89) |
| 1992 | male | 0·86 (0·8-0·91) | 1·68 (1·62-1·76) | 0·59 (0·52-0·64) | 0·38 (0·34-0·44) | 0·45 (0·37-0·56) | 0·7 (0·53-0·89) |
| 1993 | male | 0·86 (0·8-0·91) | 1·68 (1·61-1·76) | 0·6 (0·54-0·65) | 0·38 (0·34-0·44) | 0·45 (0·37-0·56) | 0·7 (0·52-0·88) |
| 1994 | male | 0·85 (0·8-0·9) | 1·66 (1·6-1·73) | 0·6 (0·54-0·66) | 0·39 (0·34-0·44) | 0·45 (0·38-0·56) | 0·7 (0·52-0·88) |
| 1995 | male | 0·84 (0·79-0·89) | 1·64 (1·58-1·71) | 0·6 (0·54-0·66) | 0·39 (0·35-0·44) | 0·45 (0·37-0·55) | 0·7 (0·52-0·87) |
| 1996 | male | 0·83 (0·77-0·88) | 1·59 (1·54-1·66) | 0·59 (0·53-0·64) | 0·39 (0·35-0·45) | 0·45 (0·38-0·55) | 0·69 (0·51-0·87) |
| 1997 | male | 0·81 (0·75-0·86) | 1·55 (1·49-1·61) | 0·58 (0·52-0·63) | 0·39 (0·35-0·45) | 0·46 (0·39-0·56) | 0·7 (0·51-0·88) |
| 1998 | male | 0·79 (0·74-0·84) | 1·49 (1·44-1·56) | 0·58 (0·51-0·62) | 0·4 (0·35-0·45) | 0·46 (0·39-0·56) | 0·69 (0·51-0·87) |
| 1999 | male | 0·77 (0·72-0·82) | 1·43 (1·37-1·49) | 0·58 (0·51-0·62) | 0·4 (0·35-0·45) | 0·46 (0·39-0·55) | 0·69 (0·5-0·87) |
| 2000 | male | 0·75 (0·69-0·8) | 1·35 (1·3-1·41) | 0·57 (0·51-0·62) | 0·4 (0·35-0·45) | 0·47 (0·39-0·56) | 0·69 (0·5-0·88) |
| 2001 | male | 0·72 (0·67-0·77) | 1·28 (1·22-1·33) | 0·56 (0·5-0·6) | 0·39 (0·34-0·44) | 0·47 (0·39-0·56) | 0·68 (0·49-0·87) |
| 2002 | male | 0·7 (0·65-0·75) | 1·21 (1·15-1·26) | 0·55 (0·5-0·59) | 0·39 (0·34-0·44) | 0·46 (0·38-0·55) | 0·68 (0·49-0·87) |
| 2003 | male | 0·68 (0·63-0·73) | 1·15 (1·1-1·21) | 0·54 (0·49-0·58) | 0·39 (0·34-0·44) | 0·46 (0·38-0·55) | 0·68 (0·49-0·86) |
| 2004 | male | 0·65 (0·61-0·7) | 1·08 (1·03-1·13) | 0·53 (0·48-0·57) | 0·39 (0·34-0·43) | 0·45 (0·38-0·54) | 0·67 (0·48-0·85) |
| 2005 | male | 0·64 (0·59-0·69) | 1·04 (0·99-1·09) | 0·52 (0·48-0·57) | 0·38 (0·33-0·43) | 0·46 (0·38-0·55) | 0·67 (0·48-0·85) |
| 2006 | male | 0·62 (0·57-0·67) | 0·99 (0·94-1·04) | 0·5 (0·45-0·54) | 0·38 (0·33-0·43) | 0·46 (0·38-0·54) | 0·67 (0·49-0·85) |
| 2007 | male | 0·61 (0·56-0·66) | 0·97 (0·92-1·01) | 0·49 (0·44-0·53) | 0·38 (0·33-0·42) | 0·46 (0·38-0·55) | 0·68 (0·5-0·86) |
| 2008 | male | 0·61 (0·56-0·65) | 0·94 (0·89-0·99) | 0·49 (0·43-0·53) | 0·38 (0·33-0·42) | 0·47 (0·39-0·55) | 0·68 (0·5-0·85) |
| 2009 | male | 0·6 (0·55-0·65) | 0·92 (0·87-0·97) | 0·48 (0·43-0·52) | 0·38 (0·32-0·43) | 0·47 (0·39-0·55) | 0·68 (0·51-0·85) |
| 2010 | male | 0·59 (0·54-0·64) | 0·89 (0·85-0·94) | 0·48 (0·42-0·52) | 0·39 (0·32-0·43) | 0·47 (0·39-0·55) | 0·68 (0·52-0·84) |
| 2011 | male | 0·57 (0·52-0·62) | 0·84 (0·79-0·88) | 0·46 (0·4-0·5) | 0·38 (0·32-0·42) | 0·47 (0·39-0·55) | 0·66 (0·5-0·82) |
| 2012 | male | 0·55 (0·5-0·6) | 0·79 (0·75-0·84) | 0·44 (0·39-0·49) | 0·38 (0·32-0·42) | 0·46 (0·39-0·55) | 0·65 (0·5-0·8) |
| 2013 | male | 0·54 (0·49-0·58) | 0·76 (0·72-0·8) | 0·43 (0·39-0·48) | 0·37 (0·32-0·41) | 0·45 (0·38-0·54) | 0·64 (0·49-0·78) |
| 2014 | male | 0·53 (0·48-0·57) | 0·73 (0·69-0·78) | 0·43 (0·38-0·48) | 0·37 (0·31-0·41) | 0·45 (0·37-0·53) | 0·63 (0·49-0·77) |
| 2015 | male | 0·53 (0·48-0·57) | 0·73 (0·69-0·77) | 0·44 (0·39-0·48) | 0·37 (0·32-0·41) | 0·44 (0·37-0·52) | 0·63 (0·49-0·76) |
| 2016 | male | 0·52 (0·48-0·57) | 0·72 (0·68-0·77) | 0·43 (0·38-0·48) | 0·38 (0·32-0·41) | 0·44 (0·37-0·52) | 0·63 (0·5-0·76) |
| 2017 | male | 0·52 (0·47-0·57) | 0·72 (0·67-0·76) | 0·43 (0·37-0·48) | 0·38 (0·32-0·42) | 0·44 (0·37-0·52) | 0·63 (0·5-0·76) |

SDI, socio-demographic index.

**Supplementary table S2. Age-standardized death rate by gender in different SDI quintiles from 1990 to 2017.**

| Year | Gender | Age-standardized death rate  per 100,000  No.(95% UI) | | | | | |
| --- | --- | --- | --- | --- | --- | --- | --- |
|  |  | Global | High SDI | High-middle SDI | Middle SDI | Low-middle SDI | Low SDI |
| 1990 | both | 0·59 (0·56-0·63) | 0·92 (0·9-0·95) | 0·4 (0·36-0·44) | 0·29 (0·26-0·33) | 0·44 (0·39-0·56) | 0·77 (0·64-0·95) |
| 1991 | both | 0·59 (0·55-0·63) | 0·91 (0·89-0·93) | 0·4 (0·36-0·44) | 0·28 (0·26-0·33) | 0·44 (0·39-0·56) | 0·77 (0·64-0·94) |
| 1992 | both | 0·58 (0·55-0·62) | 0·89 (0·87-0·91) | 0·4 (0·36-0·44) | 0·29 (0·26-0·33) | 0·44 (0·39-0·56) | 0·77 (0·64-0·94) |
| 1993 | both | 0·58 (0·55-0·61) | 0·88 (0·86-0·89) | 0·41 (0·37-0·44) | 0·28 (0·26-0·32) | 0·44 (0·39-0·55) | 0·76 (0·64-0·93) |
| 1994 | both | 0·57 (0·54-0·6) | 0·86 (0·84-0·87) | 0·41 (0·38-0·44) | 0·28 (0·26-0·32) | 0·44 (0·39-0·54) | 0·76 (0·64-0·91) |
| 1995 | both | 0·56 (0·53-0·59) | 0·84 (0·83-0·86) | 0·4 (0·37-0·43) | 0·28 (0·26-0·32) | 0·44 (0·39-0·54) | 0·75 (0·64-0·91) |
| 1996 | both | 0·55 (0·52-0·58) | 0·82 (0·8-0·83) | 0·39 (0·36-0·42) | 0·28 (0·25-0·32) | 0·44 (0·39-0·54) | 0·74 (0·63-0·89) |
| 1997 | both | 0·54 (0·51-0·57) | 0·79 (0·78-0·8) | 0·38 (0·34-0·4) | 0·28 (0·25-0·32) | 0·44 (0·4-0·55) | 0·74 (0·62-0·89) |
| 1998 | both | 0·52 (0·49-0·55) | 0·76 (0·75-0·77) | 0·37 (0·33-0·39) | 0·28 (0·25-0·31) | 0·45 (0·4-0·55) | 0·73 (0·61-0·87) |
| 1999 | both | 0·51 (0·48-0·54) | 0·72 (0·71-0·73) | 0·36 (0·33-0·38) | 0·27 (0·24-0·31) | 0·45 (0·39-0·54) | 0·72 (0·59-0·85) |
| 2000 | both | 0·49 (0·46-0·52) | 0·68 (0·67-0·69) | 0·36 (0·33-0·37) | 0·27 (0·24-0·3) | 0·45 (0·39-0·53) | 0·71 (0·59-0·85) |
| 2001 | both | 0·47 (0·44-0·5) | 0·63 (0·62-0·64) | 0·35 (0·32-0·36) | 0·27 (0·24-0·3) | 0·44 (0·38-0·53) | 0·7 (0·58-0·83) |
| 2002 | both | 0·46 (0·43-0·49) | 0·59 (0·58-0·6) | 0·34 (0·31-0·35) | 0·26 (0·23-0·29) | 0·43 (0·38-0·52) | 0·69 (0·57-0·83) |
| 2003 | both | 0·44 (0·41-0·47) | 0·56 (0·55-0·57) | 0·33 (0·31-0·34) | 0·26 (0·23-0·29) | 0·43 (0·37-0·51) | 0·69 (0·57-0·81) |
| 2004 | both | 0·42 (0·39-0·45) | 0·52 (0·51-0·53) | 0·31 (0·29-0·32) | 0·25 (0·22-0·28) | 0·42 (0·37-0·49) | 0·67 (0·55-0·79) |
| 2005 | both | 0·41 (0·38-0·43) | 0·49 (0·48-0·5) | 0·3 (0·28-0·32) | 0·25 (0·22-0·27) | 0·41 (0·36-0·48) | 0·66 (0·55-0·78) |
| 2006 | both | 0·39 (0·37-0·42) | 0·47 (0·46-0·48) | 0·28 (0·26-0·3) | 0·24 (0·21-0·26) | 0·41 (0·36-0·48) | 0·66 (0·54-0·77) |
| 2007 | both | 0·38 (0·35-0·41) | 0·45 (0·44-0·45) | 0·27 (0·25-0·29) | 0·24 (0·21-0·26) | 0·41 (0·36-0·47) | 0·66 (0·54-0·77) |
| 2008 | both | 0·38 (0·35-0·4) | 0·43 (0·42-0·44) | 0·26 (0·25-0·28) | 0·24 (0·21-0·26) | 0·41 (0·36-0·47) | 0·65 (0·54-0·76) |
| 2009 | both | 0·37 (0·34-0·39) | 0·41 (0·41-0·42) | 0·25 (0·24-0·27) | 0·23 (0·2-0·25) | 0·41 (0·36-0·47) | 0·65 (0·54-0·75) |
| 2010 | both | 0·36 (0·33-0·38) | 0·39 (0·39-0·4) | 0·25 (0·23-0·26) | 0·23 (0·2-0·25) | 0·41 (0·35-0·47) | 0·65 (0·53-0·75) |
| 2011 | both | 0·34 (0·32-0·37) | 0·37 (0·36-0·38) | 0·23 (0·22-0·25) | 0·23 (0·2-0·24) | 0·4 (0·35-0·46) | 0·63 (0·52-0·73) |
| 2012 | both | 0·33 (0·31-0·36) | 0·35 (0·34-0·36) | 0·22 (0·21-0·24) | 0·22 (0·19-0·24) | 0·39 (0·34-0·45) | 0·62 (0·51-0·71) |
| 2013 | both | 0·32 (0·3-0·35) | 0·33 (0·33-0·34) | 0·21 (0·2-0·23) | 0·22 (0·19-0·23) | 0·38 (0·34-0·44) | 0·61 (0·51-0·7) |
| 2014 | both | 0·32 (0·29-0·34) | 0·32 (0·31-0·33) | 0·21 (0·19-0·22) | 0·21 (0·19-0·23) | 0·38 (0·33-0·44) | 0·61 (0·51-0·7) |
| 2015 | both | 0·31 (0·29-0·34) | 0·32 (0·31-0·33) | 0·21 (0·19-0·22) | 0·21 (0·19-0·23) | 0·38 (0·33-0·43) | 0·61 (0·51-0·69) |
| 2016 | both | 0·31 (0·29-0·34) | 0·31 (0·31-0·32) | 0·2 (0·19-0·22) | 0·21 (0·19-0·23) | 0·37 (0·33-0·43) | 0·6 (0·51-0·69) |
| 2017 | both | 0·31 (0·28-0·33) | 0·31 (0·3-0·32) | 0·2 (0·18-0·21) | 0·21 (0·18-0·23) | 0·37 (0·32-0·43) | 0·6 (0·5-0·68) |
| 1990 | female | 0·53 (0·48-0·6) | 0·73 (0·69-0·76) | 0·34 (0·3-0·39) | 0·25 (0·21-0·33) | 0·47 (0·38-0·68) | 0·86 (0·66-1·22) |
| 1991 | female | 0·52 (0·47-0·59) | 0·72 (0·69-0·75) | 0·34 (0·29-0·39) | 0·25 (0·21-0·33) | 0·47 (0·39-0·68) | 0·86 (0·67-1·21) |
| 1992 | female | 0·51 (0·46-0·58) | 0·7 (0·67-0·72) | 0·34 (0·29-0·39) | 0·25 (0·21-0·33) | 0·47 (0·39-0·68) | 0·86 (0·67-1·21) |
| 1993 | female | 0·51 (0·46-0·58) | 0·69 (0·66-0·71) | 0·34 (0·3-0·39) | 0·25 (0·21-0·32) | 0·47 (0·38-0·67) | 0·85 (0·67-1·19) |
| 1994 | female | 0·5 (0·45-0·57) | 0·67 (0·64-0·69) | 0·35 (0·3-0·38) | 0·24 (0·21-0·31) | 0·46 (0·38-0·66) | 0·84 (0·66-1·18) |
| 1995 | female | 0·5 (0·44-0·56) | 0·66 (0·63-0·68) | 0·34 (0·3-0·38) | 0·24 (0·2-0·31) | 0·46 (0·38-0·65) | 0·83 (0·65-1·15) |
| 1996 | female | 0·48 (0·43-0·54) | 0·63 (0·6-0·65) | 0·32 (0·28-0·36) | 0·24 (0·2-0·3) | 0·46 (0·38-0·65) | 0·81 (0·64-1·12) |
| 1997 | female | 0·47 (0·42-0·53) | 0·61 (0·58-0·62) | 0·31 (0·27-0·35) | 0·24 (0·2-0·3) | 0·47 (0·38-0·66) | 0·81 (0·64-1·12) |
| 1998 | female | 0·46 (0·41-0·52) | 0·58 (0·56-0·6) | 0·3 (0·26-0·33) | 0·23 (0·2-0·29) | 0·47 (0·38-0·66) | 0·8 (0·63-1·1) |
| 1999 | female | 0·45 (0·4-0·5) | 0·55 (0·53-0·56) | 0·3 (0·26-0·32) | 0·23 (0·19-0·29) | 0·47 (0·38-0·65) | 0·78 (0·62-1·07) |
| 2000 | female | 0·43 (0·38-0·48) | 0·52 (0·5-0·53) | 0·29 (0·26-0·31) | 0·23 (0·19-0·28) | 0·47 (0·38-0·64) | 0·77 (0·6-1·05) |
| 2001 | female | 0·41 (0·37-0·47) | 0·49 (0·47-0·5) | 0·28 (0·25-0·3) | 0·22 (0·19-0·27) | 0·46 (0·37-0·62) | 0·76 (0·59-1·01) |
| 2002 | female | 0·4 (0·35-0·45) | 0·45 (0·44-0·46) | 0·27 (0·25-0·29) | 0·22 (0·18-0·26) | 0·45 (0·36-0·6) | 0·75 (0·59-1) |
| 2003 | female | 0·38 (0·34-0·44) | 0·43 (0·41-0·44) | 0·26 (0·24-0·28) | 0·21 (0·18-0·25) | 0·44 (0·36-0·59) | 0·73 (0·57-0·97) |
| 2004 | female | 0·36 (0·32-0·42) | 0·39 (0·38-0·4) | 0·25 (0·23-0·27) | 0·21 (0·18-0·25) | 0·43 (0·35-0·57) | 0·71 (0·56-0·94) |
| 2005 | female | 0·35 (0·31-0·4) | 0·37 (0·36-0·38) | 0·24 (0·22-0·26) | 0·2 (0·17-0·24) | 0·42 (0·34-0·56) | 0·7 (0·55-0·92) |
| 2006 | female | 0·34 (0·3-0·39) | 0·35 (0·34-0·36) | 0·23 (0·21-0·24) | 0·2 (0·17-0·23) | 0·42 (0·34-0·55) | 0·69 (0·54-0·9) |
| 2007 | female | 0·33 (0·29-0·38) | 0·33 (0·32-0·34) | 0·22 (0·2-0·23) | 0·19 (0·16-0·22) | 0·41 (0·33-0·53) | 0·68 (0·54-0·88) |
| 2008 | female | 0·32 (0·28-0·37) | 0·31 (0·31-0·33) | 0·21 (0·19-0·22) | 0·19 (0·16-0·22) | 0·41 (0·33-0·52) | 0·67 (0·53-0·88) |
| 2009 | female | 0·31 (0·28-0·36) | 0·3 (0·29-0·31) | 0·2 (0·19-0·22) | 0·19 (0·16-0·22) | 0·4 (0·32-0·51) | 0·67 (0·53-0·86) |
| 2010 | female | 0·31 (0·27-0·35) | 0·29 (0·28-0·3) | 0·2 (0·18-0·21) | 0·19 (0·15-0·22) | 0·4 (0·32-0·5) | 0·66 (0·52-0·85) |
| 2011 | female | 0·29 (0·26-0·34) | 0·27 (0·26-0·28) | 0·18 (0·17-0·2) | 0·18 (0·15-0·21) | 0·39 (0·31-0·49) | 0·65 (0·51-0·83) |
| 2012 | female | 0·28 (0·25-0·32) | 0·25 (0·25-0·26) | 0·17 (0·16-0·19) | 0·18 (0·14-0·2) | 0·38 (0·3-0·48) | 0·63 (0·49-0·8) |
| 2013 | female | 0·28 (0·24-0·31) | 0·24 (0·24-0·25) | 0·17 (0·16-0·18) | 0·17 (0·14-0·2) | 0·37 (0·3-0·47) | 0·63 (0·49-0·79) |
| 2014 | female | 0·27 (0·24-0·31) | 0·23 (0·23-0·25) | 0·16 (0·15-0·18) | 0·17 (0·14-0·2) | 0·38 (0·3-0·47) | 0·63 (0·48-0·79) |
| 2015 | female | 0·27 (0·23-0·31) | 0·23 (0·22-0·24) | 0·16 (0·15-0·18) | 0·17 (0·14-0·2) | 0·38 (0·3-0·47) | 0·63 (0·49-0·8) |
| 2016 | female | 0·27 (0·23-0·31) | 0·23 (0·22-0·24) | 0·15 (0·14-0·17) | 0·17 (0·14-0·2) | 0·37 (0·29-0·46) | 0·63 (0·48-0·78) |
| 2017 | female | 0·26 (0·23-0·3) | 0·23 (0·21-0·24) | 0·15 (0·14-0·17) | 0·17 (0·14-0·19) | 0·37 (0·29-0·46) | 0·62 (0·48-0·77) |
| 1990 | male | 0·69 (0·65-0·74) | 1·2 (1·17-1·23) | 0·49 (0·45-0·54) | 0·33 (0·29-0·39) | 0·42 (0·34-0·53) | 0·69 (0·52-0·86) |
| 1991 | male | 0·68 (0·64-0·73) | 1·18 (1·15-1·21) | 0·49 (0·44-0·53) | 0·33 (0·29-0·38) | 0·42 (0·34-0·53) | 0·68 (0·51-0·86) |
| 1992 | male | 0·67 (0·63-0·72) | 1·16 (1·13-1·19) | 0·49 (0·44-0·53) | 0·33 (0·29-0·39) | 0·42 (0·34-0·53) | 0·68 (0·51-0·85) |
| 1993 | male | 0·67 (0·63-0·72) | 1·14 (1·12-1·17) | 0·5 (0·46-0·54) | 0·33 (0·29-0·38) | 0·42 (0·34-0·53) | 0·68 (0·51-0·85) |
| 1994 | male | 0·66 (0·62-0·71) | 1·12 (1·1-1·15) | 0·5 (0·46-0·54) | 0·33 (0·29-0·38) | 0·42 (0·34-0·52) | 0·68 (0·5-0·85) |
| 1995 | male | 0·66 (0·62-0·7) | 1·11 (1·08-1·13) | 0·49 (0·45-0·53) | 0·33 (0·29-0·38) | 0·42 (0·34-0·51) | 0·68 (0·5-0·85) |
| 1996 | male | 0·64 (0·6-0·69) | 1·08 (1·05-1·1) | 0·48 (0·44-0·51) | 0·33 (0·29-0·38) | 0·42 (0·34-0·51) | 0·67 (0·49-0·84) |
| 1997 | male | 0·63 (0·59-0·67) | 1·04 (1·02-1·07) | 0·47 (0·43-0·5) | 0·33 (0·29-0·37) | 0·43 (0·36-0·52) | 0·67 (0·49-0·84) |
| 1998 | male | 0·62 (0·57-0·66) | 1 (0·99-1·02) | 0·46 (0·42-0·48) | 0·33 (0·29-0·37) | 0·43 (0·35-0·52) | 0·66 (0·49-0·84) |
| 1999 | male | 0·6 (0·55-0·64) | 0·95 (0·93-0·97) | 0·46 (0·42-0·48) | 0·32 (0·28-0·37) | 0·43 (0·35-0·51) | 0·65 (0·48-0·83) |
| 2000 | male | 0·58 (0·54-0·62) | 0·9 (0·88-0·91) | 0·45 (0·41-0·47) | 0·32 (0·28-0·36) | 0·43 (0·35-0·51) | 0·66 (0·48-0·84) |
| 2001 | male | 0·56 (0·52-0·6) | 0·84 (0·83-0·86) | 0·43 (0·4-0·46) | 0·32 (0·28-0·36) | 0·43 (0·35-0·51) | 0·65 (0·47-0·83) |
| 2002 | male | 0·54 (0·5-0·58) | 0·79 (0·77-0·8) | 0·42 (0·39-0·45) | 0·32 (0·28-0·35) | 0·42 (0·35-0·5) | 0·65 (0·46-0·83) |
| 2003 | male | 0·52 (0·48-0·56) | 0·75 (0·73-0·76) | 0·41 (0·38-0·44) | 0·31 (0·27-0·35) | 0·42 (0·34-0·5) | 0·64 (0·46-0·82) |
| 2004 | male | 0·5 (0·46-0·53) | 0·7 (0·68-0·71) | 0·39 (0·36-0·42) | 0·3 (0·26-0·34) | 0·41 (0·34-0·49) | 0·63 (0·45-0·81) |
| 2005 | male | 0·48 (0·45-0·52) | 0·66 (0·65-0·68) | 0·38 (0·35-0·41) | 0·3 (0·26-0·33) | 0·41 (0·34-0·49) | 0·63 (0·45-0·8) |
| 2006 | male | 0·47 (0·43-0·51) | 0·63 (0·62-0·65) | 0·36 (0·33-0·38) | 0·29 (0·25-0·33) | 0·41 (0·34-0·49) | 0·63 (0·45-0·81) |
| 2007 | male | 0·46 (0·42-0·49) | 0·61 (0·6-0·62) | 0·34 (0·31-0·37) | 0·29 (0·25-0·32) | 0·41 (0·34-0·49) | 0·64 (0·46-0·81) |
| 2008 | male | 0·45 (0·41-0·48) | 0·59 (0·57-0·6) | 0·33 (0·3-0·36) | 0·29 (0·25-0·32) | 0·42 (0·34-0·49) | 0·64 (0·47-0·81) |
| 2009 | male | 0·44 (0·4-0·47) | 0·56 (0·55-0·58) | 0·32 (0·29-0·35) | 0·29 (0·24-0·32) | 0·42 (0·34-0·49) | 0·63 (0·47-0·8) |
| 2010 | male | 0·43 (0·39-0·47) | 0·54 (0·53-0·55) | 0·31 (0·29-0·34) | 0·29 (0·24-0·32) | 0·42 (0·34-0·49) | 0·63 (0·47-0·8) |
| 2011 | male | 0·41 (0·37-0·45) | 0·5 (0·49-0·52) | 0·3 (0·27-0·32) | 0·28 (0·24-0·31) | 0·41 (0·33-0·48) | 0·62 (0·47-0·76) |
| 2012 | male | 0·4 (0·36-0·43) | 0·48 (0·47-0·49) | 0·28 (0·26-0·31) | 0·27 (0·23-0·3) | 0·41 (0·33-0·48) | 0·6 (0·46-0·74) |
| 2013 | male | 0·38 (0·35-0·42) | 0·45 (0·44-0·47) | 0·27 (0·25-0·3) | 0·27 (0·23-0·3) | 0·4 (0·33-0·47) | 0·59 (0·45-0·72) |
| 2014 | male | 0·37 (0·34-0·41) | 0·44 (0·42-0·45) | 0·27 (0·24-0·3) | 0·26 (0·22-0·29) | 0·39 (0·32-0·47) | 0·59 (0·46-0·72) |
| 2015 | male | 0·37 (0·34-0·4) | 0·43 (0·42-0·44) | 0·26 (0·24-0·3) | 0·26 (0·22-0·29) | 0·38 (0·32-0·45) | 0·58 (0·45-0·71) |
| 2016 | male | 0·37 (0·33-0·4) | 0·43 (0·41-0·44) | 0·26 (0·23-0·29) | 0·26 (0·22-0·29) | 0·38 (0·32-0·45) | 0·58 (0·46-0·71) |
| 2017 | male | 0·36 (0·33-0·39) | 0·42 (0·4-0·44) | 0·25 (0·23-0·28) | 0·26 (0·22-0·29) | 0·38 (0·31-0·45) | 0·58 (0·46-0·7) |

SDI, socio-demographic index.

**Supplementary table S3. Age-standardized DALY rate by gender in different SDI quintiles from 1990 to 2017.**

| year | gender | Age-standardized DALY rate  per 100,000  No.(95% UI) | | | | | |
| --- | --- | --- | --- | --- | --- | --- | --- |
|  |  | Global | High SDI | High-middle SDI | Middle SDI | Low-middle SDI | Low SDI |
| 1990 | both | 15·96 (14·6-17·44) | 24·23 (23·53-24·96) | 12·17 (10·67-13·57) | 8·68 (7·68-10·09) | 13·25 (11·49-16·8) | 23·14 (18·45-29·19) |
| 1991 | both | 15·8 (14·46-17·18) | 23·8 (23·2-24·43) | 12·02 (10·49-13·42) | 8·66 (7·69-10) | 13·33 (11·57-16·74) | 23·05 (18·44-28·69) |
| 1992 | both | 15·64 (14·31-16·99) | 23·11 (22·55-23·73) | 12·1 (10·57-13·46) | 8·68 (7·72-10·05) | 13·35 (11·55-16·83) | 23·09 (18·46-28·57) |
| 1993 | both | 15·53 (14·27-16·75) | 22·65 (22·13-23·15) | 12·37 (10·92-13·56) | 8·64 (7·67-9·97) | 13·23 (11·44-16·6) | 22·95 (18·62-27·93) |
| 1994 | both | 15·33 (14·08-16·49) | 22·02 (21·54-22·51) | 12·42 (11·02-13·5) | 8·6 (7·63-9·86) | 13·14 (11·39-16·44) | 22·78 (18·46-27·66) |
| 1995 | both | 15·12 (13·89-16·26) | 21·55 (21·07-21·98) | 12·21 (10·8-13·28) | 8·57 (7·58-9·77) | 13·05 (11·34-16·2) | 22·64 (18·39-27·45) |
| 1996 | both | 14·75 (13·54-15·87) | 20·79 (20·37-21·2) | 11·73 (10·33-12·74) | 8·52 (7·48-9·7) | 13·03 (11·27-16·15) | 22·32 (18·17-27·05) |
| 1997 | both | 14·45 (13·2-15·55) | 20·05 (19·64-20·45) | 11·24 (9·88-12·22) | 8·47 (7·38-9·59) | 13·27 (11·49-16·43) | 22·22 (18·09-26·87) |
| 1998 | both | 14·1 (12·84-15·18) | 19·13 (18·74-19·52) | 10·88 (9·57-11·74) | 8·4 (7·31-9·45) | 13·34 (11·56-16·36) | 21·95 (17·91-26·37) |
| 1999 | both | 13·72 (12·47-14·77) | 18·06 (17·71-18·41) | 10·74 (9·53-11·48) | 8·3 (7·19-9·3) | 13·31 (11·52-16·17) | 21·61 (17·68-25·78) |
| 2000 | both | 13·29 (12·06-14·3) | 16·88 (16·55-17·21) | 10·45 (9·35-11·06) | 8·22 (7·1-9·14) | 13·27 (11·45-15·95) | 21·35 (17·38-25·21) |
| 2001 | both | 12·73 (11·57-13·67) | 15·66 (15·36-15·98) | 10·03 (9·06-10·55) | 7·95 (6·94-8·83) | 13·04 (11·27-15·65) | 20·93 (16·97-24·82) |
| 2002 | both | 12·22 (11·14-13·08) | 14·41 (14·13-14·72) | 9·65 (8·83-10·05) | 7·8 (6·86-8·63) | 12·79 (11·13-15·26) | 20·54 (16·71-24·26) |
| 2003 | both | 11·73 (10·72-12·59) | 13·43 (13·18-13·71) | 9·22 (8·5-9·66) | 7·59 (6·7-8·38) | 12·51 (10·91-14·92) | 20·14 (16·46-23·84) |
| 2004 | both | 11·18 (10·23-12·04) | 12·33 (12·11-12·58) | 8·75 (8·07-9·21) | 7·34 (6·49-8·13) | 12·16 (10·51-14·41) | 19·62 (16·1-23·23) |
| 2005 | both | 10·81 (9·89-11·66) | 11·55 (11·35-11·76) | 8·44 (7·83-8·91) | 7·16 (6·34-7·89) | 12·03 (10·46-14·1) | 19·26 (15·86-22·74) |
| 2006 | both | 10·45 (9·53-11·29) | 10·85 (10·66-11·06) | 7·86 (7·26-8·27) | 7 (6·2-7·7) | 11·97 (10·45-13·94) | 19·12 (15·71-22·3) |
| 2007 | both | 10·16 (9·25-10·95) | 10·28 (10·1-10·49) | 7·48 (6·91-7·88) | 6·85 (6·05-7·54) | 11·84 (10·4-13·74) | 19·01 (15·68-22·06) |
| 2008 | both | 9·95 (9·04-10·73) | 9·77 (9·6-9·96) | 7·23 (6·69-7·63) | 6·8 (5·94-7·44) | 11·79 (10·34-13·66) | 18·89 (15·54-21·86) |
| 2009 | both | 9·73 (8·82-10·48) | 9·3 (9·13-9·51) | 6·9 (6·36-7·34) | 6·75 (5·88-7·38) | 11·73 (10·29-13·58) | 18·7 (15·41-21·52) |
| 2010 | both | 9·53 (8·63-10·29) | 8·85 (8·68-9·04) | 6·67 (6·17-7·12) | 6·69 (5·77-7·28) | 11·65 (10·2-13·45) | 18·53 (15·32-21·29) |
| 2011 | both | 9·13 (8·28-9·84) | 8·22 (8·06-8·4) | 6·24 (5·78-6·68) | 6·5 (5·62-7·06) | 11·35 (9·95-13·15) | 17·98 (14·92-20·51) |
| 2012 | both | 8·8 (7·98-9·51) | 7·69 (7·54-7·87) | 5·95 (5·51-6·38) | 6·34 (5·54-6·88) | 11·06 (9·67-12·85) | 17·51 (14·61-19·94) |
| 2013 | both | 8·53 (7·76-9·27) | 7·29 (7·14-7·47) | 5·72 (5·34-6·16) | 6·21 (5·4-6·7) | 10·81 (9·44-12·64) | 17·18 (14·42-19·63) |
| 2014 | both | 8·37 (7·61-9·13) | 6·98 (6·82-7·17) | 5·54 (5·16-6·01) | 6·11 (5·29-6·59) | 10·71 (9·32-12·51) | 17·11 (14·36-19·46) |
| 2015 | both | 8·3 (7·54-9·04) | 6·88 (6·7-7·08) | 5·52 (5·14-5·99) | 6·07 (5·27-6·52) | 10·54 (9·15-12·24) | 17·02 (14·22-19·28) |
| 2016 | both | 8·25 (7·48-8·97) | 6·85 (6·66-7·1) | 5·39 (5·02-5·86) | 6·08 (5·25-6·51) | 10·47 (9·09-12·17) | 16·89 (14·13-19·03) |
| 2017 | both | 8·17 (7·42-8·88) | 6·81 (6·57-7·08) | 5·26 (4·9-5·76) | 6·03 (5·25-6·47) | 10·33 (8·95-12·07) | 16·71 (14·03-18·84) |
| 1990 | female | 14·78 (12·72-17·36) | 19·06 (18·16-19·93) | 10·58 (8·85-12·56) | 7·97 (6·46-10·66) | 14·89 (11·88-21·19) | 27·17 (19·96-39·45) |
| 1991 | female | 14·63 (12·56-17·05) | 18·72 (17·92-19·46) | 10·43 (8·65-12·44) | 7·92 (6·44-10·4) | 14·99 (11·98-21·05) | 27·01 (20-38·8) |
| 1992 | female | 14·48 (12·34-16·93) | 18·01 (17·29-18·74) | 10·44 (8·63-12·45) | 7·93 (6·42-10·29) | 15·08 (11·91-21·15) | 27·12 (20·03-39·16) |
| 1993 | female | 14·36 (12·29-16·8) | 17·69 (16·98-18·29) | 10·67 (8·92-12·33) | 7·83 (6·35-9·99) | 14·87 (11·73-20·79) | 26·83 (19·95-38·43) |
| 1994 | female | 14·13 (12·08-16·53) | 17·08 (16·42-17·64) | 10·7 (9·02-12·15) | 7·73 (6·25-9·82) | 14·69 (11·51-20·5) | 26·53 (19·65-37·73) |
| 1995 | female | 13·92 (11·88-16·26) | 16·71 (15·94-17·24) | 10·47 (8·76-11·9) | 7·65 (6·17-9·67) | 14·62 (11·41-20·26) | 26·27 (19·52-37·43) |
| 1996 | female | 13·55 (11·52-15·84) | 16·04 (15·29-16·55) | 9·95 (8·29-11·38) | 7·55 (6·07-9·49) | 14·63 (11·46-20·16) | 25·79 (19·29-36·44) |
| 1997 | female | 13·25 (11·23-15·56) | 15·38 (14·72-15·86) | 9·46 (7·87-10·84) | 7·47 (5·99-9·41) | 14·85 (11·63-20·43) | 25·58 (19·06-36·13) |
| 1998 | female | 12·9 (10·88-15·12) | 14·61 (14·03-15·04) | 9·07 (7·61-10·32) | 7·37 (5·94-9·19) | 14·91 (11·64-20·38) | 25·19 (18·94-35·29) |
| 1999 | female | 12·58 (10·62-14·78) | 13·76 (13·27-14·14) | 8·91 (7·57-9·92) | 7·27 (5·86-9·01) | 14·87 (11·57-20·1) | 24·78 (18·75-34·69) |
| 2000 | female | 12·18 (10·28-14·35) | 12·82 (12·37-13·18) | 8·59 (7·4-9·42) | 7·14 (5·77-8·81) | 14·77 (11·5-19·82) | 24·36 (18·35-34·11) |
| 2001 | female | 11·7 (9·94-13·77) | 11·87 (11·46-12·23) | 8·28 (7·21-8·96) | 6·9 (5·62-8·48) | 14·48 (11·37-19·31) | 23·77 (17·95-32·96) |
| 2002 | female | 11·22 (9·59-13·18) | 10·9 (10·54-11·23) | 7·97 (7·06-8·53) | 6·71 (5·55-8·15) | 14·13 (11·11-18·69) | 23·23 (17·65-31·93) |
| 2003 | female | 10·76 (9·25-12·57) | 10·12 (9·79-10·44) | 7·6 (6·83-8·15) | 6·49 (5·41-7·75) | 13·79 (10·9-18·07) | 22·65 (17·4-30·74) |
| 2004 | female | 10·25 (8·86-11·94) | 9·25 (8·98-9·55) | 7·17 (6·46-7·71) | 6·25 (5·23-7·42) | 13·36 (10·58-17·35) | 22 (16·92-29·64) |
| 2005 | female | 9·89 (8·55-11·54) | 8·61 (8·37-8·92) | 6·9 (6·23-7·43) | 6·05 (5·06-7·18) | 13·11 (10·37-17·01) | 21·44 (16·52-28·78) |
| 2006 | female | 9·54 (8·21-11·14) | 8·03 (7·8-8·32) | 6·4 (5·77-6·88) | 5·89 (4·92-6·93) | 12·96 (10·22-16·67) | 21·12 (16·1-28·33) |
| 2007 | female | 9·24 (7·94-10·78) | 7·52 (7·32-7·82) | 6·1 (5·5-6·59) | 5·71 (4·77-6·67) | 12·72 (10·02-16·19) | 20·81 (15·81-27·7) |
| 2008 | female | 9·03 (7·74-10·49) | 7·12 (6·94-7·41) | 5·88 (5·33-6·39) | 5·64 (4·69-6·51) | 12·56 (9·89-15·83) | 20·55 (15·59-27·16) |
| 2009 | female | 8·81 (7·51-10·2) | 6·76 (6·57-7·04) | 5·6 (5·06-6·08) | 5·58 (4·64-6·44) | 12·4 (9·79-15·44) | 20·25 (15·46-26·59) |
| 2010 | female | 8·61 (7·31-9·91) | 6·35 (6·17-6·6) | 5·38 (4·87-5·86) | 5·53 (4·56-6·39) | 12·27 (9·63-15·23) | 19·99 (15·37-26·16) |
| 2011 | female | 8·28 (7·01-9·55) | 5·95 (5·79-6·19) | 5·01 (4·57-5·48) | 5·33 (4·41-6·21) | 11·92 (9·39-14·92) | 19·42 (14·93-25·18) |
| 2012 | female | 7·96 (6·72-9·18) | 5·56 (5·38-5·82) | 4·74 (4·34-5·22) | 5·18 (4·27-6) | 11·51 (9·12-14·26) | 18·85 (14·52-24·3) |
| 2013 | female | 7·77 (6·55-8·98) | 5·29 (5·1-5·54) | 4·54 (4·18-5·07) | 5·1 (4·18-5·9) | 11·3 (8·9-14·08) | 18·59 (14·25-23·79) |
| 2014 | female | 7·67 (6·44-8·88) | 5·07 (4·87-5·34) | 4·36 (4·02-4·92) | 5·02 (4·11-5·8) | 11·33 (8·82-14·02) | 18·63 (14·12-23·62) |
| 2015 | female | 7·62 (6·38-8·81) | 4·99 (4·8-5·27) | 4·31 (3·98-4·88) | 4·97 (4·11-5·73) | 11·23 (8·72-13·84) | 18·65 (14·02-23·59) |
| 2016 | female | 7·57 (6·34-8·79) | 4·98 (4·75-5·29) | 4·19 (3·86-4·76) | 4·96 (4·13-5·67) | 11·12 (8·56-13·73) | 18·46 (13·94-23·32) |
| 2017 | female | 7·48 (6·27-8·68) | 4·94 (4·66-5·29) | 4·06 (3·74-4·63) | 4·89 (4·06-5·6) | 10·96 (8·45-13·41) | 18·21 (13·73-22·98) |
| 1990 | male | 17·48 (16·08-18·92) | 30·39 (29·55-31·34) | 14·1 (12·34-15·47) | 9·47 (8·25-10·96) | 11·7 (9·68-14·76) | 19·31 (14·51-24·12) |
| 1991 | male | 17·28 (15·91-18·66) | 29·85 (29·07-30·71) | 13·94 (12·21-15·28) | 9·47 (8·31-10·97) | 11·76 (9·75-14·83) | 19·28 (14·49-24·14) |
| 1992 | male | 17·11 (15·75-18·51) | 29·15 (28·41-29·93) | 14·09 (12·37-15·32) | 9·51 (8·36-10·99) | 11·72 (9·78-14·7) | 19·25 (14·52-24) |
| 1993 | male | 17·01 (15·72-18·32) | 28·54 (27·87-29·28) | 14·39 (12·79-15·62) | 9·52 (8·41-10·97) | 11·68 (9·75-14·54) | 19·24 (14·37-24·06) |
| 1994 | male | 16·84 (15·55-18·09) | 27·87 (27·22-28·55) | 14·45 (12·89-15·84) | 9·55 (8·46-10·9) | 11·67 (9·78-14·39) | 19·22 (14·41-24·03) |
| 1995 | male | 16·61 (15·32-17·82) | 27·28 (26·68-27·94) | 14·25 (12·69-15·69) | 9·56 (8·41-10·91) | 11·56 (9·72-14·2) | 19·18 (14·41-24·03) |
| 1996 | male | 16·25 (14·94-17·44) | 26·41 (25·86-27·03) | 13·79 (12·25-15·07) | 9·57 (8·38-10·94) | 11·52 (9·67-14·13) | 19·01 (14·23-23·9) |
| 1997 | male | 15·94 (14·57-17·13) | 25·56 (25·03-26·12) | 13·31 (11·75-14·42) | 9·54 (8·3-10·91) | 11·77 (9·91-14·34) | 19 (14·17-23·78) |
| 1998 | male | 15·57 (14·21-16·76) | 24·46 (23·98-24·99) | 12·97 (11·46-13·94) | 9·5 (8·27-10·87) | 11·84 (9·95-14·31) | 18·82 (13·98-23·72) |
| 1999 | male | 15·12 (13·77-16·27) | 23·1 (22·65-23·59) | 12·84 (11·42-13·76) | 9·41 (8·15-10·73) | 11·82 (9·9-14·25) | 18·56 (13·72-23·52) |
| 2000 | male | 14·66 (13·31-15·81) | 21·63 (21·21-22·09) | 12·58 (11·26-13·48) | 9·37 (8·09-10·61) | 11·84 (9·8-14·21) | 18·45 (13·56-23·37) |
| 2001 | male | 14·02 (12·76-15·17) | 20·09 (19·69-20·52) | 12·04 (10·85-12·76) | 9·07 (7·86-10·24) | 11·68 (9·74-14·03) | 18·18 (13·32-23·1) |
| 2002 | male | 13·46 (12·25-14·61) | 18·51 (18·16-18·93) | 11·59 (10·53-12·25) | 8·97 (7·8-10·09) | 11·52 (9·6-13·86) | 17·93 (13·1-22·85) |
| 2003 | male | 12·94 (11·77-14·09) | 17·31 (16·95-17·73) | 11·09 (10·16-11·75) | 8·77 (7·62-9·84) | 11·31 (9·37-13·64) | 17·71 (12·98-22·61) |
| 2004 | male | 12·33 (11·18-13·49) | 15·94 (15·63-16·31) | 10·56 (9·67-11·28) | 8·51 (7·39-9·56) | 11·01 (9·11-13·39) | 17·32 (12·66-22·18) |
| 2005 | male | 11·96 (10·83-13·08) | 14·98 (14·68-15·34) | 10·21 (9·37-10·93) | 8·34 (7·22-9·39) | 11·03 (9·2-13·26) | 17·15 (12·58-21·88) |
| 2006 | male | 11·57 (10·42-12·71) | 14·16 (13·86-14·47) | 9·52 (8·68-10·18) | 8·18 (7·06-9·19) | 11·03 (9·23-13·13) | 17·19 (12·7-21·99) |
| 2007 | male | 11·28 (10·1-12·4) | 13·5 (13·2-13·8) | 9·06 (8·22-9·68) | 8·06 (6·9-9·03) | 11·01 (9·21-13·16) | 17·26 (12·79-21·89) |
| 2008 | male | 11·08 (9·9-12·17) | 12·86 (12·57-13·15) | 8·76 (7·94-9·38) | 8·05 (6·85-8·96) | 11·07 (9·26-13·25) | 17·28 (12·9-21·63) |
| 2009 | male | 10·84 (9·64-11·9) | 12·27 (12·01-12·55) | 8·39 (7·55-9·01) | 8·01 (6·78-8·86) | 11·12 (9·21-13·24) | 17·2 (12·99-21·42) |
| 2010 | male | 10·63 (9·46-11·66) | 11·75 (11·49-12·02) | 8·14 (7·34-8·8) | 7·94 (6·69-8·77) | 11·1 (9·15-13·18) | 17·11 (12·97-21·12) |
| 2011 | male | 10·16 (9·05-11·14) | 10·84 (10·59-11·11) | 7·65 (6·88-8·26) | 7·75 (6·53-8·55) | 10·85 (9·02-12·94) | 16·58 (12·74-20·37) |
| 2012 | male | 9·81 (8·74-10·78) | 10·17 (9·93-10·43) | 7·31 (6·58-7·94) | 7·59 (6·42-8·36) | 10·68 (8·92-12·82) | 16·2 (12·6-19·8) |
| 2013 | male | 9·47 (8·45-10·4) | 9·59 (9·36-9·86) | 7·04 (6·36-7·72) | 7·4 (6·26-8·19) | 10·39 (8·78-12·54) | 15·81 (12·41-19·29) |
| 2014 | male | 9·23 (8·23-10·16) | 9·17 (8·94-9·46) | 6·85 (6·19-7·53) | 7·29 (6·13-8) | 10·17 (8·62-12·25) | 15·62 (12·32-19) |
| 2015 | male | 9·13 (8·15-10·03) | 9·04 (8·79-9·33) | 6·86 (6·18-7·55) | 7·24 (6·11-7·94) | 9·93 (8·39-11·94) | 15·43 (12·23-18·76) |
| 2016 | male | 9·08 (8·09-9·98) | 8·99 (8·69-9·31) | 6·72 (6·04-7·4) | 7·27 (6·15-7·97) | 9·88 (8·35-11·87) | 15·35 (12·1-18·63) |
| 2017 | male | 9 (8·01-9·9) | 8·93 (8·56-9·31) | 6·59 (5·9-7·25) | 7·24 (6·16-7·96) | 9·77 (8·28-11·79) | 15·24 (12·15-18·43) |

DALY, disability-adjusted life year; SDI, socio-demographic index.

**Supplementary table S4. Incident cases by gender and the ratio of male to female by age groups in different SDI quintiles in 2017.**

|  | Global | | ratio | High SDI | | ratio | High-middle SDI | | ratio | Middle SDI | | ratio | Low-middle SDI | | ratio | Low SDI | | ratio |
| --- | --- | --- | --- | --- | --- | --- | --- | --- | --- | --- | --- | --- | --- | --- | --- | --- | --- | --- |
|  | female | male |  | female | male |  | female | male |  | female | male |  | female | male |  | female | male |  |
| ＜5 | 0 | 0 | / | 0 | 0 | / | 0 | 0 | / | 0 | 0 | / | 0 | 0 | / | 0 | 0 | / |
| 5 to 9 | 0 | 0 | / | 0 | 0 | / | 0 | 0 | / | 0 | 0 | / | 0 | 0 | / | 0 | 0 | / |
| 10 to 14 | 0 | 0 | / | 0 | 0 | / | 0 | 0 | / | 0 | 0 | / | 0 | 0 | / | 0 | 0 | / |
| 15 to 19 | 306·68 | 285·95 | 0·93 | 22·52 | 31·99 | 1·42 | 36·08 | 53·63 | 1·49 | 63·07 | 81·35 | 1·29 | 93·08 | 49·51 | 0·53 | 91·31 | 68·55 | 0·75 |
| 20 to 24 | 462·29 | 482·99 | 1·04 | 32·67 | 60·13 | 1·84 | 41·24 | 74·94 | 1·82 | 89·72 | 132·86 | 1·48 | 152·39 | 94·9 | 0·62 | 145·51 | 118·98 | 0·82 |
| 25 to 29 | 775 | 1000·71 | 1·29 | 74·25 | 137·89 | 1·86 | 100·14 | 169·75 | 1·7 | 155·48 | 238·52 | 1·53 | 225·83 | 224·97 | 1 | 217·28 | 226·83 | 1·04 |
| 30 to 34 | 885·11 | 1154·27 | 1·3 | 99·32 | 195·06 | 1·96 | 129·31 | 250·23 | 1·94 | 180·32 | 292·04 | 1·62 | 239·82 | 210·42 | 0·88 | 234·15 | 203·23 | 0·87 |
| 35 to 39 | 873·02 | 1121·47 | 1·28 | 127·69 | 220·84 | 1·73 | 123·83 | 227·28 | 1·84 | 176 | 303·26 | 1·72 | 217·6 | 196·12 | 0·9 | 226·19 | 171·18 | 0·76 |
| 40 to 44 | 928·45 | 1075·32 | 1·16 | 138·81 | 228·47 | 1·65 | 134·59 | 235·45 | 1·75 | 213·18 | 280·38 | 1·32 | 227·89 | 174·62 | 0·77 | 212·27 | 153·38 | 0·72 |
| 45 to 49 | 1018·01 | 1311·71 | 1·29 | 203·79 | 298·27 | 1·46 | 190·38 | 312·07 | 1·64 | 226·11 | 351·18 | 1·55 | 197·62 | 185·31 | 0·94 | 197·16 | 159·89 | 0·81 |
| 50 to 54 | 1371·18 | 1497·85 | 1·09 | 237·77 | 373·01 | 1·57 | 220·4 | 350·16 | 1·59 | 292·75 | 396·22 | 1·35 | 328·24 | 206·95 | 0·63 | 288·62 | 164·98 | 0·57 |
| 55 to 59 | 1186·21 | 1476·1 | 1·24 | 225·97 | 398·09 | 1·76 | 198·05 | 285·69 | 1·44 | 258·73 | 367·14 | 1·42 | 260·83 | 228·4 | 0·88 | 240·59 | 192·6 | 0·8 |
| 60 to 64 | 1180·67 | 1546·26 | 1·31 | 247·11 | 414·06 | 1·68 | 205·88 | 348·17 | 1·69 | 224·29 | 344·53 | 1·54 | 248·25 | 232·1 | 0·93 | 252·87 | 202·94 | 0·8 |
| 65 to 69 | 1271·18 | 1718·64 | 1·35 | 337·06 | 520·35 | 1·54 | 241·19 | 373·26 | 1·55 | 224·32 | 362·46 | 1·62 | 235·81 | 237·07 | 1·01 | 230·36 | 220·15 | 0·96 |
| 70 to 74 | 1119·34 | 1505·45 | 1·34 | 353·58 | 586·66 | 1·66 | 147·29 | 242·51 | 1·65 | 196·83 | 274·97 | 1·4 | 212·73 | 201·33 | 0·95 | 207·3 | 196·74 | 0·95 |
| 75 to 79 | 1154·37 | 1913·89 | 1·66 | 504·38 | 926·97 | 1·84 | 189·98 | 297·16 | 1·56 | 157·85 | 307·41 | 1·95 | 154·87 | 203·15 | 1·31 | 145·71 | 175·43 | 1·2 |
| 80 to 84 | 1056·6 | 1545·98 | 1·46 | 568·29 | 889·4 | 1·57 | 135·91 | 172·55 | 1·27 | 133·76 | 191·56 | 1·43 | 118·69 | 159·73 | 1·35 | 98·58 | 130·29 | 1·32 |
| 85 to 89 | 828·19 | 1006·75 | 1·22 | 562·24 | 707·86 | 1·26 | 81·72 | 88·59 | 1·08 | 83·61 | 100·34 | 1·2 | 57·46 | 59·22 | 1·03 | 42·16 | 49·37 | 1·17 |
| 90 to 94 | 456·18 | 411·55 | 0·9 | 372·61 | 323·88 | 0·87 | 35·36 | 28·86 | 0·82 | 26·94 | 31·75 | 1·18 | 12·76 | 15·73 | 1·23 | 7·96 | 10·76 | 1·35 |
| ＞95 | 161 | 90·38 | 0·56 | 142·59 | 76·84 | 0·54 | 8·46 | 5·24 | 0·62 | 6·59 | 5·64 | 0·86 | 2·2 | 1·72 | 0·78 | 0·99 | 0·82 | 0·83 |

SDI, socio-demographic index.

**Supplementary table S5. Incident cases by age groups and region in 1990.**

|  | under5 | 5 to 9 | 10 to 14 | 15 to 19 | 20 to 24 | 25 to 29 | 30 to 34 | 35 to 39 | 40 to 44 | 45 to 49 | 50 to 54 | 55 to 59 | 60 to 64 | 65 to 69 | 70 to 74 | 75 to 79 | 80 to 84 | 85 to 89 | 90 to 94 | 95 plus |
| --- | --- | --- | --- | --- | --- | --- | --- | --- | --- | --- | --- | --- | --- | --- | --- | --- | --- | --- | --- | --- |
| global | 0 | 0 | 0 | 731·08 | 1183·68 | 1932·16 | 2033·06 | 2031·47 | 1962·39 | 1964·99 | 2357·54 | 2535·19 | 2786·51 | 2922·74 | 2501·96 | 2966·13 | 2186·54 | 1143·17 | 416·8 | 97·01 |
| SDI |  |  |  |  |  |  |  |  |  |  |  |  |  |  |  |  |  |  |  |  |
| high SDI | 0 | 0 | 0 | 147·42 | 266·04 | 538·5 | 713·08 | 806·08 | 896·84 | 939·5 | 1084·66 | 1258·83 | 1377·48 | 1644·06 | 1494·96 | 2099·19 | 1685·64 | 936·79 | 364·51 | 88·76 |
| high-middle SDI | 0 | 0 | 0 | 141·54 | 224·12 | 374·34 | 400·54 | 367·09 | 316·25 | 304·23 | 422·27 | 425·57 | 535·91 | 470·19 | 340·1 | 353·9 | 179·44 | 75·76 | 22·07 | 4·19 |
| middle SDI | 0 | 0 | 0 | 159·09 | 267·15 | 383·84 | 338·37 | 328·24 | 284·64 | 268·05 | 299·29 | 313·04 | 291·36 | 279·18 | 234·63 | 193·39 | 115·62 | 57·63 | 15·42 | 2·45 |
| low-middle SDI | 0 | 0 | 0 | 139·21 | 206·46 | 316·28 | 285·09 | 257·94 | 234·44 | 221·61 | 277·78 | 270·49 | 279·94 | 254·82 | 207·07 | 162·83 | 114·86 | 40·93 | 8·97 | 1·09 |
| low SDI | 0 | 0 | 0 | 141·31 | 215·29 | 312·18 | 290·24 | 267·07 | 226·41 | 227·81 | 269·48 | 262·89 | 297·53 | 269·93 | 221·33 | 153·08 | 88·56 | 30·83 | 5·4 | 0·43 |
| region |  |  |  |  |  |  |  |  |  |  |  |  |  |  |  |  |  |  |  |  |
| Andean Latin America | 0 | 0 | 0 | 4·98 | 6·74 | 9·68 | 5·37 | 8·31 | 6·57 | 5·56 | 5·55 | 5·95 | 6·05 | 6·85 | 4·91 | 4·72 | 3·01 | 1·77 | 0·79 | 0·15 |
| Australasia | 0 | 0 | 0 | 3·24 | 7·21 | 14·87 | 15·25 | 17·99 | 18·29 | 15·12 | 18·27 | 18·78 | 26·45 | 43·69 | 48·75 | 58·84 | 42·77 | 26·77 | 8·75 | 1·66 |
| Caribbean | 0 | 0 | 0 | 4·39 | 12·53 | 18·85 | 16·5 | 17·13 | 15·43 | 17·08 | 21·11 | 20·39 | 23·33 | 22·52 | 23·03 | 23·9 | 15·33 | 8·98 | 3·27 | 0·63 |
| Central Asia | 0 | 0 | 0 | 13·94 | 8·25 | 17·58 | 24·47 | 23·33 | 12·58 | 16·03 | 28·2 | 31·1 | 33·33 | 18·95 | 8·11 | 8·49 | 4·89 | 1·9 | 0·66 | 0·1 |
| Central Europe | 0 | 0 | 0 | 15·46 | 22·77 | 41·58 | 69·98 | 75·53 | 73·68 | 62·29 | 91·07 | 110·36 | 112·34 | 116·64 | 74·19 | 132·79 | 76·75 | 28·07 | 6·84 | 0·96 |
| Central Latin America | 0 | 0 | 0 | 22·71 | 42·92 | 56·67 | 60·49 | 61 | 57·64 | 55·31 | 56·43 | 55·5 | 50·26 | 46·31 | 44·75 | 38·79 | 25·92 | 16·96 | 5·23 | 1·11 |
| Central Sub Saharan Africa | 0 | 0 | 0 | 5·48 | 9·74 | 15·03 | 12·74 | 12·23 | 8·51 | 10·53 | 13·48 | 14·16 | 14·61 | 13·47 | 9·91 | 5·73 | 2·3 | 0·76 | 0·18 | 0·02 |
| East Asia | 0 | 0 | 0 | 130·8 | 228·24 | 333·36 | 267·87 | 217·51 | 154·84 | 138·68 | 135·51 | 146·93 | 132·6 | 138·54 | 108·56 | 77·26 | 35·7 | 13·29 | 3·52 | 0·32 |
| Eastern Europe | 0 | 0 | 0 | 28·31 | 39·48 | 79·75 | 120·82 | 105·65 | 107·37 | 102·39 | 184·07 | 160·81 | 263·45 | 214·09 | 143·78 | 162·09 | 65·39 | 18·38 | 4·76 | 0·89 |
| Eastern Sub Saharan Africa | 0 | 0 | 0 | 49·69 | 81·03 | 111·96 | 96·42 | 92·23 | 76·57 | 78·86 | 85·75 | 91·26 | 98·64 | 93·34 | 80·78 | 48·39 | 18·76 | 5·72 | 0·94 | 0·1 |
| High income Asia Pacific | 0 | 0 | 0 | 38·84 | 70·82 | 101·67 | 124·87 | 132·11 | 145·98 | 149·59 | 152·54 | 151·77 | 132·09 | 107·07 | 116·03 | 111·85 | 93·28 | 52·71 | 21·97 | 5·27 |
| High income North America | 0 | 0 | 0 | 36·35 | 70·23 | 172·77 | 243·64 | 256·67 | 278·62 | 269·58 | 246·27 | 224·82 | 259·76 | 275·46 | 277·25 | 315·67 | 252·06 | 153·57 | 60·74 | 16·95 |
| North Africa and Middle East | 0 | 0 | 0 | 46·93 | 68·24 | 108·06 | 91·43 | 90·62 | 73·11 | 77·79 | 92·79 | 106·87 | 114·1 | 106·83 | 79·46 | 61·72 | 39·8 | 20·23 | 5·97 | 0·97 |
| Oceania | 0 | 0 | 0 | 0·32 | 1·7 | 5·43 | 3·5 | 4·1 | 1·27 | 3·78 | 3·49 | 2·73 | 2·69 | 1·97 | 1·66 | 0·3 | 0·19 | 0·07 | 0 | 0 |
| South Asia | 0 | 0 | 0 | 212·43 | 298·96 | 394·25 | 377·72 | 354·77 | 313·03 | 301·45 | 336·93 | 353·04 | 421·77 | 361·76 | 305·75 | 240·65 | 154·06 | 59·01 | 10·63 | 0·83 |
| Southeast Asia | 0 | 0 | 0 | 38·66 | 71·28 | 110·42 | 106·15 | 109 | 105·22 | 86·73 | 115·58 | 126·04 | 95·17 | 95·97 | 71·25 | 55·62 | 33·1 | 16·54 | 3·67 | 0·42 |
| Southern Latin America | 0 | 0 | 0 | 4·65 | 8·54 | 19·65 | 17·92 | 23·9 | 24·72 | 27·63 | 33·42 | 31·68 | 38·63 | 36·52 | 35·69 | 34·68 | 22·7 | 12·06 | 3·63 | 1·08 |
| Southern Sub Saharan Africa | 0 | 0 | 0 | 1·14 | 1·4 | 2·64 | 2·79 | 2·68 | 3·52 | 3·15 | 3·09 | 2·76 | 2·77 | 2·61 | 2·04 | 1·3 | 0·71 | 0·33 | 0·11 | 0·02 |
| Tropical Latin America | 0 | 0 | 0 | 18·51 | 35·06 | 58·31 | 62·02 | 66·68 | 59·3 | 59·92 | 63·62 | 63·81 | 59·78 | 51·34 | 46·71 | 36·74 | 20·93 | 12·54 | 3·75 | 0·66 |
| Western Europe | 0 | 0 | 0 | 51·91 | 91·55 | 191·24 | 246·37 | 325·66 | 391·72 | 456·58 | 601·31 | 778·15 | 875·81 | 1135·63 | 1002·04 | 1529·03 | 1248·41 | 689·33 | 270·42 | 64·78 |

SDI, socio-demographic index.

**Supplementary table S6. Death cases by age groups and region in 1990.**

|  | under5 | 5 to 9 | 10 to 14 | 15 to 19 | 20 to 24 | 25 to 29 | 30 to 34 | 35 to 39 | 40 to 44 | 45 to 49 | 50 to 54 | 55 to 59 | 60 to 64 | 65 to 69 | 70 to 74 | 75 to 79 | 80 to 84 | 85 to 89 | 90 to 94 | 95 plus |
| --- | --- | --- | --- | --- | --- | --- | --- | --- | --- | --- | --- | --- | --- | --- | --- | --- | --- | --- | --- | --- |
| global | 0 | 0 | 0 | 485·61 | 695·07 | 953·38 | 1080·55 | 1196·9 | 1259·05 | 1313·26 | 1683·78 | 1962·18 | 2359·21 | 2545·03 | 2258·63 | 2655·65 | 2138·38 | 1116·72 | 401·35 | 93·23 |
| SDI |  |  |  |  |  |  |  |  |  |  |  |  |  |  |  |  |  |  |  |  |
| high SDI | 0 | 0 | 0 | 76·47 | 128·07 | 213·45 | 297·45 | 375·05 | 457·54 | 511·97 | 642·12 | 844·36 | 1028·74 | 1280 | 1215·97 | 1721·98 | 1514·01 | 859·83 | 336·81 | 83·15 |
| high-middle SDI | 0 | 0 | 0 | 85·74 | 124·45 | 178·23 | 206·87 | 217·95 | 211·4 | 214·12 | 325·33 | 367·85 | 505·55 | 455·19 | 344·78 | 362·05 | 212·98 | 90·72 | 26·48 | 5·03 |
| middle SDI | 0 | 0 | 0 | 108·94 | 160·39 | 197·23 | 195·99 | 215·28 | 209·51 | 204·06 | 236·56 | 265·5 | 267·66 | 272·53 | 238·11 | 209 | 143·74 | 71·4 | 18·99 | 3·01 |
| low-middle SDI | 0 | 0 | 0 | 101·89 | 131·39 | 174·93 | 181·48 | 185·32 | 188·02 | 183·31 | 237·09 | 240·26 | 265·58 | 256·26 | 218·04 | 183·38 | 148·76 | 52·91 | 11·53 | 1·39 |
| low SDI | 0 | 0 | 0 | 111·01 | 148·14 | 186·12 | 195·82 | 200·37 | 190·15 | 197·24 | 239·81 | 240·88 | 288·12 | 277·11 | 238·26 | 175·8 | 116·46 | 40·62 | 7·1 | 0·57 |
| region |  |  |  |  |  |  |  |  |  |  |  |  |  |  |  |  |  |  |  |  |
| Andean Latin America | 0 | 0 | 0 | 3·54 | 4·14 | 5·04 | 3·21 | 5·65 | 4·99 | 4·39 | 4·5 | 5·13 | 5·59 | 6·79 | 5·02 | 5·16 | 3·8 | 2·24 | 0·99 | 0·19 |
| Australasia | 0 | 0 | 0 | 1·58 | 3·39 | 5·94 | 6·83 | 9·11 | 10·35 | 9·19 | 11·74 | 15·97 | 23·76 | 31·82 | 42·28 | 45·17 | 36·64 | 23·44 | 7·77 | 1·52 |
| Caribbean | 0 | 0 | 0 | 2·82 | 7·26 | 9·51 | 9·23 | 10·89 | 10·88 | 12·28 | 16·38 | 17·62 | 22·06 | 22·37 | 23·34 | 25·13 | 18·13 | 10·54 | 3·86 | 0·74 |
| Central Asia | 0 | 0 | 0 | 9·08 | 4·72 | 9·06 | 13·85 | 15·29 | 9·21 | 12·58 | 23·08 | 27·53 | 31·12 | 19·14 | 8·26 | 9·03 | 6·02 | 2·31 | 0·8 | 0·12 |
| Central Europe | 0 | 0 | 0 | 8·55 | 11·93 | 18·83 | 36·26 | 46·78 | 49·45 | 46·75 | 71·58 | 99·61 | 111·11 | 120·8 | 77·07 | 141·21 | 92·49 | 33·88 | 8·41 | 1·2 |
| Central Latin America | 0 | 0 | 0 | 14·94 | 24·8 | 27·76 | 34·51 | 39·49 | 42·12 | 41·82 | 44·38 | 47·16 | 46·39 | 45·49 | 45·49 | 41·81 | 32·22 | 21·06 | 6·47 | 1·38 |
| Central Sub Saharan Africa | 0 | 0 | 0 | 4·5 | 6·89 | 9·1 | 8·62 | 9·17 | 7·2 | 9·01 | 11·87 | 12·9 | 14·09 | 13·69 | 10·56 | 6·54 | 3·02 | 0·99 | 0·23 | 0·03 |
| East Asia | 0 | 0 | 0 | 80·25 | 128·49 | 160·01 | 133·03 | 122·92 | 97·76 | 92·68 | 93·53 | 112·33 | 111·32 | 123·83 | 101·19 | 76·91 | 40·09 | 14·8 | 3·86 | 0·34 |
| Eastern Europe | 0 | 0 | 0 | 14·97 | 20·36 | 35·58 | 58·02 | 58·43 | 67·66 | 67·23 | 142·94 | 146·25 | 256·5 | 204·67 | 148·62 | 158·79 | 72·99 | 20·37 | 5·3 | 1 |
| Eastern Sub Saharan Africa | 0 | 0 | 0 | 41·05 | 57·55 | 67·65 | 66·43 | 70·41 | 65·75 | 69·76 | 78·32 | 85·55 | 97·02 | 97·17 | 87·98 | 56·08 | 24·81 | 7·56 | 1·24 | 0·13 |
| High income Asia Pacific | 0 | 0 | 0 | 18·61 | 31·82 | 39·94 | 52·82 | 66·18 | 79·75 | 80·95 | 86·74 | 95·9 | 100·47 | 84·1 | 84·07 | 80·68 | 69·98 | 39·71 | 16·81 | 4·07 |
| High income North America | 0 | 0 | 0 | 17·59 | 30·34 | 61·43 | 92·55 | 114·21 | 130·49 | 143·58 | 153·9 | 196·99 | 274·31 | 329·65 | 347·27 | 363·57 | 300·68 | 186·01 | 75·26 | 21·47 |
| North Africa and Middle East | 0 | 0 | 0 | 33·07 | 41·61 | 56·08 | 53·83 | 60·36 | 54·28 | 60·14 | 74·5 | 91·14 | 104·66 | 104·66 | 80·78 | 66·94 | 49·94 | 25·3 | 7·42 | 1·21 |
| Oceania | 0 | 0 | 0 | 0·24 | 1·14 | 2·98 | 2·12 | 2·91 | 1·01 | 3·13 | 2·99 | 2·47 | 2·6 | 2 | 1·76 | 0·33 | 0·24 | 0·08 | 0·01 | 0 |
| South Asia | 0 | 0 | 0 | 158·2 | 195·32 | 223·19 | 245·65 | 259·59 | 255·67 | 255·02 | 292·86 | 316·82 | 402·72 | 367·17 | 324·94 | 273·8 | 201·72 | 77·29 | 13·91 | 1·09 |
| Southeast Asia | 0 | 0 | 0 | 27·61 | 43·98 | 58·01 | 62·77 | 73·05 | 78·83 | 67·12 | 92·87 | 107·78 | 87·24 | 93·62 | 72·33 | 60·24 | 41·02 | 20·44 | 4·49 | 0·5 |
| Southern Latin America | 0 | 0 | 0 | 2·97 | 4·76 | 9·52 | 9·96 | 15·13 | 17·39 | 20·18 | 25·67 | 26·21 | 34·8 | 35·15 | 35·36 | 36·49 | 27·49 | 14·65 | 4·41 | 1·33 |
| Southern Sub Saharan Africa | 0 | 0 | 0 | 0·83 | 0·87 | 1·38 | 1·65 | 1·79 | 2·7 | 2·45 | 2·47 | 2·35 | 2·54 | 2·55 | 2·07 | 1·41 | 0·89 | 0·41 | 0·14 | 0·02 |
| Tropical Latin America | 0 | 0 | 0 | 12·56 | 20·89 | 29·71 | 36·32 | 44·28 | 44·07 | 46·25 | 50·98 | 54·56 | 55·46 | 50·52 | 47·53 | 39·76 | 26·13 | 15·67 | 4·68 | 0·82 |
| Western Europe | 0 | 0 | 0 | 30·74 | 49·99 | 81·94 | 108·06 | 144·98 | 200·34 | 245·7 | 342·01 | 463·53 | 553·36 | 756·64 | 694·41 | 1146·93 | 1050·96 | 594·65 | 234·06 | 55·96 |

SDI, socio-demographic index.

**Supplementary table S7. Incident cases by age groups and region in 2017.**

|  | under5 | 5 to 9 | 10 to 14 | 15 to 19 | 20 to 24 | 25 to 29 | 30 to 34 | 35 to 39 | 40 to 44 | 45 to 49 | 50 to 54 | 55 to 59 | 60 to 64 | 65 to 69 | 70 to 74 | 75 to 79 | 80 to 84 | 85 to 89 | 90 to 94 | 95 plus |
| --- | --- | --- | --- | --- | --- | --- | --- | --- | --- | --- | --- | --- | --- | --- | --- | --- | --- | --- | --- | --- |
| global | 0 | 0 | 0 | 592·64 | 945·28 | 1775·7 | 2039·38 | 1994·49 | 2003·77 | 2329·71 | 2869·03 | 2662·31 | 2726·93 | 2989·81 | 2624·79 | 3068·27 | 2602·58 | 1834·94 | 867·73 | 251·37 |
| SDI |  |  |  |  |  |  |  |  |  |  |  |  |  |  |  |  |  |  |  |  |
| high SDI | 0 | 0 | 0 | 54·52 | 92·8 | 212·14 | 294·38 | 348·53 | 367·28 | 502·06 | 610·79 | 624·06 | 661·18 | 857·42 | 940·24 | 1431·35 | 1457·7 | 1270·11 | 696·49 | 219·43 |
| high-middle SDI | 0 | 0 | 0 | 89·71 | 116·18 | 269·89 | 379·54 | 351·11 | 370·03 | 502·45 | 570·56 | 483·75 | 554·05 | 614·45 | 389·8 | 487·15 | 308·46 | 170·32 | 64·21 | 13·7 |
| middle SDI | 0 | 0 | 0 | 144·42 | 222·58 | 394 | 472·36 | 479·26 | 493·55 | 577·29 | 688·97 | 625·88 | 568·83 | 586·79 | 471·8 | 465·26 | 325·32 | 183·95 | 58·69 | 12·23 |
| low-middle SDI | 0 | 0 | 0 | 142·59 | 247·29 | 450·8 | 450·24 | 413·72 | 402·51 | 382·93 | 535·19 | 489·23 | 480·35 | 472·88 | 414·06 | 358·02 | 278·42 | 116·68 | 28·49 | 3·92 |
| low SDI | 0 | 0 | 0 | 159·86 | 264·49 | 444·11 | 437·38 | 397·38 | 365·65 | 357·04 | 453·6 | 433·2 | 455·81 | 450·51 | 404·04 | 321·14 | 228·87 | 91·53 | 18·72 | 1·81 |
| region |  |  |  |  |  |  |  |  |  |  |  |  |  |  |  |  |  |  |  |  |
| Andean Latin America | 0 | 0 | 0 | 7·8 | 11·21 | 18·43 | 11·55 | 18·55 | 16·14 | 14·29 | 15·35 | 14·43 | 14·01 | 16·98 | 12·55 | 13·71 | 10·75 | 6·9 | 3·87 | 1·06 |
| Australasia | 0 | 0 | 0 | 2·39 | 5 | 10·04 | 13·44 | 16·88 | 17·71 | 17·32 | 19·11 | 19·59 | 20·76 | 30·45 | 33·97 | 41·53 | 41·16 | 41·17 | 21·96 | 5·5 |
| Caribbean | 0 | 0 | 0 | 2·94 | 7·54 | 14·77 | 16·43 | 16·21 | 16·59 | 23·52 | 31·51 | 28·73 | 31·07 | 28·89 | 26·12 | 27·58 | 18·06 | 9·64 | 5·69 | 2·16 |
| Central Asia | 0 | 0 | 0 | 11·88 | 7·17 | 16·39 | 22·11 | 23·63 | 14·99 | 27·1 | 29·05 | 36·58 | 29·42 | 23·83 | 7·99 | 12·9 | 7·49 | 2·45 | 0·84 | 0·08 |
| Central Europe | 0 | 0 | 0 | 7·41 | 10·05 | 20·68 | 29·02 | 26·3 | 28·2 | 30·68 | 41·24 | 50·86 | 58·11 | 69·09 | 57·83 | 106·62 | 80·82 | 47·76 | 14·7 | 2·69 |
| Central Latin America | 0 | 0 | 0 | 23·8 | 46·31 | 69·1 | 79·2 | 89·86 | 92·76 | 100·16 | 108·1 | 105·88 | 88·83 | 86·47 | 90·3 | 90 | 63·41 | 42·29 | 14·98 | 4·59 |
| Central Sub Saharan Africa | 0 | 0 | 0 | 12·41 | 20·06 | 30·9 | 28·32 | 27·71 | 24·29 | 29·55 | 36·3 | 35·97 | 34·51 | 28·88 | 21·36 | 15·91 | 9·46 | 3·9 | 0·91 | 0·12 |
| East Asia | 0 | 0 | 0 | 76·39 | 76·73 | 204·26 | 250·78 | 180·27 | 211·44 | 374·3 | 470·7 | 248·69 | 280·41 | 345·1 | 172·27 | 181·93 | 113·3 | 57·28 | 23·57 | 3·11 |
| Eastern Europe | 0 | 0 | 0 | 12·82 | 20·24 | 55·93 | 118·61 | 105·22 | 119·7 | 140·77 | 134·52 | 173·3 | 219·83 | 238·02 | 139·16 | 210·04 | 103·75 | 41·81 | 15·52 | 2·1 |
| Eastern Sub Saharan Africa | 0 | 0 | 0 | 73·71 | 114·49 | 158·29 | 146·84 | 140·15 | 114·73 | 109·19 | 113·91 | 124·42 | 134·62 | 125·66 | 113·93 | 92·86 | 51·52 | 20·13 | 4·16 | 0·42 |
| High income Asia Pacific | 0 | 0 | 0 | 12·39 | 20·38 | 28·65 | 44·9 | 60·24 | 65·51 | 92·61 | 92·03 | 85·67 | 89·54 | 113·14 | 102·23 | 119·43 | 129·79 | 112·65 | 76·99 | 34·69 |
| High income North America | 0 | 0 | 0 | 13·14 | 30·14 | 79·65 | 100·82 | 110·61 | 112·13 | 148·53 | 167·54 | 154·65 | 143·7 | 154·1 | 168·21 | 188·83 | 192·28 | 166·87 | 92·73 | 30·88 |
| North Africa and Middle East | 0 | 0 | 0 | 51·25 | 77·31 | 141·71 | 146·5 | 161·64 | 142·5 | 158·45 | 162·55 | 139·61 | 146·77 | 143·51 | 107·25 | 105·17 | 82·61 | 48·14 | 14·54 | 2·55 |
| Oceania | 0 | 0 | 0 | 0·47 | 2·6 | 8·45 | 5·98 | 7·14 | 2·14 | 6·73 | 6·54 | 4·73 | 4·07 | 2·88 | 2·7 | 0·5 | 0·39 | 0·16 | 0·01 | 0 |
| South Asia | 0 | 0 | 0 | 192·44 | 341·55 | 524·01 | 585·62 | 567·8 | 542·96 | 520 | 663·6 | 682·54 | 743·63 | 732·86 | 702·35 | 579·34 | 423·73 | 196·9 | 40·84 | 3·79 |
| Southeast Asia | 0 | 0 | 0 | 52·01 | 86·08 | 139·5 | 162·41 | 187·34 | 212·14 | 210·93 | 259·92 | 288·56 | 217·84 | 221·25 | 156·43 | 150·38 | 97·71 | 55·95 | 16·45 | 2·4 |
| Southern Latin America | 0 | 0 | 0 | 3·56 | 6·48 | 13·81 | 12·31 | 15·85 | 15·86 | 18·64 | 23·83 | 20·85 | 24·53 | 28·16 | 29·14 | 29·28 | 25·65 | 22·09 | 7·65 | 2·35 |
| Southern Sub Saharan Africa | 0 | 0 | 0 | 1·75 | 1·98 | 2·96 | 3·07 | 3·21 | 4·1 | 4·28 | 4·71 | 4·81 | 4·99 | 4·66 | 3·41 | 2·41 | 1·29 | 0·6 | 0·22 | 0·03 |
| Tropical Latin America | 0 | 0 | 0 | 12·74 | 22·87 | 36·84 | 45·46 | 52·94 | 44·73 | 50·91 | 63·06 | 66·01 | 63·34 | 65·63 | 67·8 | 65·44 | 48·46 | 38·11 | 16·27 | 4·77 |
| Western Europe | 0 | 0 | 0 | 16·48 | 23·86 | 57·03 | 78·42 | 116·19 | 130·3 | 193·61 | 273·51 | 297·51 | 335·52 | 476·11 | 583·7 | 998·55 | 1029·73 | 910·15 | 493·26 | 147·79 |

SDI, socio-demographic index.

**Supplementary table S8. Death cases by age groups and region in 2017.**

|  | under5 | 5 to 9 | 10 to 14 | 15 to 19 | 20 to 24 | 25 to 29 | 30 to 34 | 35 to 39 | 40 to 44 | 45 to 49 | 50 to 54 | 55 to 59 | 60 to 64 | 65 to 69 | 70 to 74 | 75 to 79 | 80 to 84 | 85 to 89 | 90 to 94 | 95 plus |
| --- | --- | --- | --- | --- | --- | --- | --- | --- | --- | --- | --- | --- | --- | --- | --- | --- | --- | --- | --- | --- |
| global | 0 | 0 | 0 | 306·29 | 464·6 | 730·14 | 880·29 | 959·35 | 1056·21 | 1196·65 | 1619·88 | 1819·27 | 2078·21 | 2408·49 | 2359·56 | 2703·54 | 2597·22 | 1790·72 | 838·55 | 245·28 |
| SDI |  |  |  |  |  |  |  |  |  |  |  |  |  |  |  |  |  |  |  |  |
| high SDI | 0 | 0 | 0 | 15·6 | 28·26 | 52·06 | 70·81 | 91·06 | 104·96 | 159·67 | 234·07 | 337·81 | 423·33 | 579·54 | 725·32 | 1055·34 | 1228·63 | 1115·6 | 637·21 | 207·01 |
| high-middle SDI | 0 | 0 | 0 | 25·93 | 38·99 | 75·71 | 103·97 | 117·6 | 134·25 | 180·89 | 232·18 | 293·54 | 379·45 | 442·72 | 339·29 | 425·74 | 327·83 | 186·67 | 71·01 | 16·17 |
| middle SDI | 0 | 0 | 0 | 62·8 | 97·45 | 143·58 | 186·81 | 222·18 | 256·23 | 300·08 | 380·41 | 426·69 | 432·69 | 486·14 | 443·62 | 460·92 | 379·77 | 216·52 | 68·5 | 14·47 |
| low-middle SDI | 0 | 0 | 0 | 87·94 | 133·65 | 215·89 | 246·28 | 253·14 | 278·3 | 270·99 | 400·04 | 389·19 | 419·54 | 449·48 | 422·56 | 394·39 | 358·54 | 150·29 | 36·44 | 5 |
| low SDI | 0 | 0 | 0 | 113·6 | 165·55 | 241·49 | 270·98 | 273·93 | 280·96 | 282·47 | 369·77 | 368·9 | 419·34 | 446·07 | 425·09 | 363·18 | 299·17 | 119·61 | 24·37 | 2·35 |
| region |  |  |  |  |  |  |  |  |  |  |  |  |  |  |  |  |  |  |  |  |
| Andean Latin America | 0 | 0 | 0 | 3·8 | 4·95 | 6·87 | 4·9 | 9·12 | 8·99 | 8·32 | 9·24 | 10·03 | 10·94 | 14·93 | 11·87 | 14·17 | 13·17 | 8·49 | 4·73 | 1·28 |
| Australasia | 0 | 0 | 0 | 0·48 | 1·3 | 2·33 | 2·94 | 3·95 | 4·53 | 5·17 | 6·81 | 10·74 | 12·68 | 16·5 | 23·18 | 29·12 | 33·65 | 35·23 | 19·52 | 5·28 |
| Caribbean | 0 | 0 | 0 | 1·46 | 3·72 | 6·26 | 7·43 | 8·37 | 9·57 | 13·44 | 20·34 | 22·17 | 26·6 | 27·33 | 26·46 | 28·98 | 21·76 | 11·55 | 6·9 | 2·59 |
| Central Asia | 0 | 0 | 0 | 5·38 | 3·07 | 6·65 | 9·43 | 12·17 | 8·66 | 17·31 | 20·07 | 30·3 | 25·46 | 23·87 | 8·16 | 13·55 | 9·36 | 3·03 | 1·04 | 0·09 |
| Central Europe | 0 | 0 | 0 | 1·79 | 3·05 | 5·64 | 8·7 | 11·55 | 14·9 | 18·74 | 27·04 | 46·06 | 55·69 | 75·15 | 64·35 | 115·23 | 105·49 | 64·15 | 20·55 | 3·98 |
| Central Latin America | 0 | 0 | 0 | 10·4 | 18·81 | 23·82 | 31·48 | 41·41 | 49·23 | 55·69 | 64·8 | 74·39 | 71 | 77·4 | 87·79 | 93·93 | 78·5 | 52·3 | 18·5 | 5·63 |
| Central Sub Saharan Africa | 0 | 0 | 0 | 9·39 | 13·29 | 17·69 | 18·15 | 19·92 | 19·3 | 23·84 | 29·93 | 31 | 31·92 | 28·68 | 22·51 | 18·03 | 12·39 | 5·11 | 1·19 | 0·15 |
| East Asia | 0 | 0 | 0 | 15·99 | 21·59 | 46·74 | 47·86 | 37·26 | 47·11 | 88·3 | 118·76 | 94·01 | 128·85 | 162·72 | 110·97 | 113·75 | 83·71 | 41·93 | 17·2 | 2·21 |
| Eastern Europe | 0 | 0 | 0 | 3·63 | 7·11 | 17·37 | 34·5 | 36·72 | 46·15 | 57·87 | 76·02 | 121·23 | 169·4 | 187·82 | 128·59 | 181·68 | 107·92 | 43·13 | 15·83 | 2·17 |
| Eastern Sub Saharan Africa | 0 | 0 | 0 | 54·54 | 74·43 | 88·33 | 94·53 | 100·82 | 92·94 | 91·7 | 98·65 | 112 | 128·94 | 129·14 | 123·34 | 107·32 | 68·25 | 26·63 | 5·48 | 0·55 |
| High income Asia Pacific | 0 | 0 | 0 | 2·35 | 4·31 | 5·22 | 8·01 | 10·98 | 14·52 | 19·31 | 22·66 | 28·09 | 36·41 | 51·91 | 59·8 | 73·33 | 94·28 | 83·83 | 58·98 | 27 |
| High income North America | 0 | 0 | 0 | 4·6 | 9·66 | 19·25 | 24·83 | 29·63 | 30·75 | 44·86 | 60·68 | 90·81 | 116·16 | 147·37 | 186·21 | 207·68 | 225·62 | 203·42 | 117·98 | 40·79 |
| North Africa and Middle East | 0 | 0 | 0 | 25·43 | 35·64 | 55·14 | 60·18 | 75·63 | 76·04 | 88·96 | 96·43 | 95·24 | 113·94 | 125·58 | 102·75 | 105·51 | 98·01 | 57·04 | 17·26 | 3·03 |
| Oceania | 0 | 0 | 0 | 0·31 | 1·56 | 4·29 | 3·3 | 4·69 | 1·53 | 5·19 | 5·13 | 3·99 | 3·8 | 2·85 | 2·86 | 0·54 | 0·49 | 0·2 | 0·01 | 0 |
| South Asia | 0 | 0 | 0 | 122·75 | 192·02 | 251·37 | 327·43 | 356·92 | 384·46 | 379·86 | 504·32 | 549·81 | 657·12 | 704·78 | 722·21 | 644·13 | 550·07 | 255·1 | 52·71 | 4·89 |
| Southeast Asia | 0 | 0 | 0 | 25·27 | 38·11 | 51·78 | 61·1 | 83·35 | 110·69 | 115·73 | 155·33 | 196·53 | 162·77 | 183·02 | 140·86 | 142·45 | 104·76 | 59·99 | 17·05 | 2·37 |
| Southern Latin America | 0 | 0 | 0 | 1·54 | 2·56 | 4·65 | 4·72 | 6·85 | 7·65 | 9·42 | 12·96 | 13·51 | 17·93 | 23·38 | 26·86 | 28·89 | 29·81 | 26·01 | 9·15 | 2·86 |
| Southern Sub Saharan Africa | 0 | 0 | 0 | 1·1 | 1·09 | 1·39 | 1·63 | 1·95 | 2·88 | 2·98 | 3·3 | 3·68 | 4·21 | 4·33 | 3·4 | 2·59 | 1·64 | 0·76 | 0·28 | 0·04 |
| Tropical Latin America | 0 | 0 | 0 | 5·92 | 9·87 | 13·62 | 19·21 | 25·65 | 24·56 | 29·58 | 39·35 | 47·61 | 51·41 | 59·43 | 66·48 | 69·24 | 60·45 | 47·79 | 20·44 | 6·06 |
| Western Europe | 0 | 0 | 0 | 6·63 | 10·18 | 17·91 | 23·73 | 35·43 | 44·08 | 75·13 | 123·02 | 172·1 | 214·81 | 310·04 | 413·66 | 674·07 | 807·1 | 752·38 | 430·48 | 133·91 |

SDI, socio-demographic index.

**Supplementary table S9. The incidence, death and DALY rates by gender and age groups in 1990.**

| Age groups | Age-standardized incidence rate  per 100,000  No.(95% UI) | | | Age-standardized death rate  per 100,000  No.(95% UI) | | | Age-standardized DALY rate  per 100,000  No.(95% UI) | |
| --- | --- | --- | --- | --- | --- | --- | --- | --- |
|  | Female | Male |  | Female | Male |  | Female | Male |
| ＜5 | 0 | 0 |  | 0 | 0 |  | 0 | 0 |
| 5 to 9 | 0 | 0 |  | 0 | 0 |  | 0 | 0 |
| 10 to 14 | 0 | 0 |  | 0 | 0 |  | 0 | 0 |
| 15 to 19 | 0·15 (0·12-0·2) | 0·13 (0·11-0·15) |  | 0·1 (0·08-0·14) | 0·08 (0·07-0·1) |  | 7·31 (5·46-9·66) | 5·79 (4·81-6·82) |
| 20 to 24 | 0·24 (0·18-0·33) | 0·23 (0·2-0·26) |  | 0·15 (0·11-0·21) | 0·13 (0·11-0·15) |  | 9·69 (6·99-13·65) | 8·81 (7·27-10·24) |
| 25 to 29 | 0·42 (0·3-0·54) | 0·45 (0·38-0·51) |  | 0·21 (0·15-0·28) | 0·22 (0·18-0·25) |  | 12·82 (9·07-16·92) | 13·4 (11·34-15·33) |
| 30 to 34 | 0·49 (0·38-0·61) | 0·55 (0·48-0·61) |  | 0·27 (0·2-0·34) | 0·29 (0·25-0·32) |  | 15·02 (11·1-19·15) | 16·27 (13·97-18·26) |
| 35 to 39 | 0·53 (0·43-0·64) | 0·61 (0·54-0·68) |  | 0·32 (0·25-0·39) | 0·36 (0·31-0·4) |  | 16·24 (12·88-20·15) | 18·27 (15·9-20·35) |
| 40 to 44 | 0·64 (0·55-0·75) | 0·71 (0·64-0·79) |  | 0·42 (0·35-0·5) | 0·45 (0·41-0·5) |  | 19·32 (16·19-23·34) | 21·02 (18·86-23·19) |
| 45 to 49 | 0·8 (0·69-0·92) | 0·88 (0·8-0·98) |  | 0·53 (0·45-0·63) | 0·59 (0·53-0·66) |  | 22·02 (18·69-26·19) | 24·47 (21·95-27·21) |
| 50 to 54 | 1·07 (0·94-1·25) | 1·13 (1·02-1·25) |  | 0·76 (0·66-0·92) | 0·81 (0·73-0·9) |  | 28·06 (24·04-33·86) | 29·6 (26·87-32·89) |
| 55 to 59 | 1·23 (1·09-1·46) | 1·48 (1·34-1·66) |  | 0·98 (0·87-1·18) | 1·12 (1·03-1·24) |  | 31·14 (27·64-37·65) | 35·9 (32·76-39·58) |
| 60 to 64 | 1·62 (1·43-1·9) | 1·83 (1·66-2·03) |  | 1·35 (1·22-1·59) | 1·58 (1·45-1·73) |  | 36·68 (33·18-43·38) | 43·01 (39·38-47·22) |
| 65 to 69 | 2·15 (1·91-2·52) | 2·59 (2·34-2·87) |  | 1·79 (1·64-2·1) | 2·35 (2·18-2·6) |  | 40·8 (37·36-47·77) | 53·46 (49·6-59·11) |
| 70 to 74 | 2·49 (2·25-2·89) | 3·5 (3·18-3·85) |  | 2·25 (2·05-2·62) | 3·16 (2·94-3·46) |  | 41·55 (37·86-48·42) | 58·49 (54·31-63·88) |
| 75 to 79 | 3·6 (3·23-4·09) | 6·52 (5·92-7·27) |  | 3·33 (3·07-3·68) | 5·69 (5·25-6·16) |  | 47·92 (44·2-52·83) | 82·23 (76·02-88·91) |
| 80 to 84 | 4·85 (4·29-5·5) | 8·15 (7·41-9·03) |  | 4·76 (4·36-5·25) | 7·95 (7·38-8·65) |  | 51·24 (46·93-56·57) | 86·05 (79·89-93·65) |
| 85 to 89 | 6·17 (5·48-7·08) | 9·91 (9·08-10·94) |  | 6·07 (5·6-6·63) | 9·6 (8·95-10·34) |  | 47·11 (43·35-51·56) | 74·95 (70·06-80·55) |
| 90 to 94 | 8·4 (7·46-9·58) | 12·35 (11·25-13·77) |  | 8·12 (7·48-8·87) | 11·82 (11-12·71) |  | 43·7 (40·17-47·68) | 64·06 (59·46-68·81) |
| 95 plus | 10·67 (9·49-12·1) | 13·51 (12·16-15·11) |  | 10·41 (9·58-11·24) | 12·52 (11·64-13·55) |  | 32·99 (30·22-35·83) | 40·96 (37·86-44·27) |

**Supplementary table S10. The incidence, death and DALY rates by gender and age groups in 2017.**

| Age groups | Age-standardized incidence rate  per 100,000  No.(95% UI) | | | Age-standardized death rate  per 100,000  No.(95% UI) | | | Age-standardized DALY rate  per 100,000  No.(95% UI) | |
| --- | --- | --- | --- | --- | --- | --- | --- | --- |
|  | Female | Male |  | Female | Male |  | Female | Male |
| ＜5 | 0 | 0 |  | 0 | 0 |  | 0 | 0 |
| 5 to 9 | 0 | 0 |  | 0 | 0 |  | 0 | 0 |
| 10 to 14 | 0 | 0 |  | 0 | 0 |  | 0 | 0 |
| 15 to 19 | 0·1 (0·08-0·13) | 0·09 (0·08-0·11) |  | 0·06 (0·04-0·07) | 0·04 (0·04-0·05) |  | 3·99 (3·17-5·3) | 3·1 (2·55-3·69) |
| 20 to 24 | 0·15 (0·12-0·2) | 0·16 (0·14-0·18) |  | 0·08 (0·06-0·11) | 0·07 (0·06-0·08) |  | 5·34 (4·19-7·01) | 4·86 (4·11-5·63) |
| 25 to 29 | 0·25 (0·2-0·31) | 0·32 (0·28-0·38) |  | 0·11 (0·08-0·13) | 0·13 (0·11-0·15) |  | 6·57 (5·09-8·21) | 8 (6·84-9·51) |
| 30 to 34 | 0·3 (0·24-0·36) | 0·39 (0·34-0·44) |  | 0·14 (0·11-0·17) | 0·16 (0·14-0·18) |  | 8·04 (6·26-9·69) | 8·92 (7·73-10·12) |
| 35 to 39 | 0·34 (0·27-0·4) | 0·42 (0·37-0·48) |  | 0·17 (0·14-0·21) | 0·19 (0·16-0·22) |  | 8·94 (6·97-10·79) | 9·87 (8·37-11·19) |
| 40 to 44 | 0·38 (0·32-0·44) | 0·43 (0·38-0·49) |  | 0·22 (0·18-0·26) | 0·21 (0·18-0·24) |  | 10·1 (8·12-11·96) | 9·82 (8·59-11·26) |
| 45 to 49 | 0·44 (0·36-0·51) | 0·55 (0·48-0·63) |  | 0·24 (0·19-0·28) | 0·27 (0·23-0·31) |  | 9·89 (7·75-11·56) | 11·3 (9·65-12·83) |
| 50 to 54 | 0·64 (0·52-0·77) | 0·71 (0·62-0·8) |  | 0·4 (0·32-0·47) | 0·37 (0·32-0·42) |  | 14·6 (11·59-17·22) | 13·58 (11·76-15·32) |
| 55 to 59 | 0·67 (0·57-0·78) | 0·86 (0·75-0·97) |  | 0·48 (0·41-0·57) | 0·56 (0·49-0·63) |  | 15·51 (13-18·35) | 18·1 (15·92-20·15) |
| 60 to 64 | 0·75 (0·65-0·92) | 1·04 (0·9-1·15) |  | 0·62 (0·53-0·74) | 0·74 (0·65-0·83) |  | 16·86 (14·62-20·21) | 20·39 (17·71-22·6) |
| 65 to 69 | 1·01 (0·88-1·18) | 1·49 (1·31-1·65) |  | 0·83 (0·72-0·99) | 1·18 (1·05-1·31) |  | 18·98 (16·36-22·56) | 26·99 (23·94-30·03) |
| 70 to 74 | 1·26 (1·1-1·47) | 1·91 (1·69-2·12) |  | 1·19 (1·04-1·41) | 1·65 (1·47-1·81) |  | 21·88 (19·21-26·1) | 30·53 (27·15-33·56) |
| 75 to 79 | 1·69 (1·48-1·96) | 3·47 (3·05-3·94) |  | 1·58 (1·42-1·84) | 2·94 (2·66-3·2) |  | 22·7 (20·38-26·54) | 42·41 (38·27-46·38) |
| 80 to 84 | 2·26 (1·96-2·57) | 4·62 (4·11-5·18) |  | 2·38 (2·11-2·69) | 4·44 (4·01-4·84) |  | 25·44 (22·56-28·7) | 47·84 (43·23-52·17) |
| 85 to 89 | 3·19 (2·78-3·67) | 6·39 (5·68-7·21) |  | 3·31 (3·02-3·66) | 5·93 (5·51-6·41) |  | 25·51 (23·33-28·36) | 46·12 (42·9-49·73) |
| 90 to 94 | 4·38 (3·74-5·05) | 8·27 (7·26-9·53) |  | 4·44 (4·06-4·84) | 7·55 (7·05-8·18) |  | 23·67 (21·53-25·8) | 40·66 (37·81-44·25) |
| 95 plus | 5·85 (4·99-6·89) | 8·84 (7·58-10·27) |  | 5·94 (5·39-6·55) | 8 (7·31-8·7) |  | 18·13 (16·29-20·02) | 25·34 (23·22-27·7) |

**Supplementary table S11. Incident cases and ASIR by countries in 1990 and 2017 and its change trends from 1990 to 2017.**

| Nation | Sex | Incident cases  No.×10^2^  (95% UI) | | Change (%) | ASIR  per 100,000  No.(95% UI) | | EAPC |
| --- | --- | --- | --- | --- | --- | --- | --- |
|  |  | 1990 | 2017 |  | 1990 | 2017 |  |
| Afghanistan | both | 66·77(26·66-126·87) | 145·54(71·54-260·07) | 117·96 | 0·94(0·39-1·79) | 0·87(0·41-1·42) | -0·4(-4·92-4·35) |
| Albania | both | 11·72(8·24-18·52) | 13·06(8·15-22·25) | 11·48 | 0·44(0·31-0·73) | 0·38(0·24-0·6) | -0·72(-7·47-6·53) |
| Algeria | both | 104·24(82·66-126·29) | 210·61(160·86-252·57) | 102·04 | 0·69(0·55-0·85) | 0·58(0·45-0·68) | -0·44(-5·92-5·37) |
| American Samoa | both | 0·14(0·11-0·17) | 0·16(0·13-0·2) | 13·48 | 0·48(0·36-0·58) | 0·33(0·27-0·41) | -1·66(-8·45-5·63) |
| Andorra | both | 0·66(0·5-0·85) | 0·78(0·59-1·02) | 18·05 | 1·11(0·84-1·44) | 0·59(0·45-0·78) | -2·73(-7·56-2·35) |
| Angola | both | 31·5(18·59-44·09) | 79·88(52·35-108·3) | 153·58 | 0·58(0·38-0·78) | 0·58(0·38-0·79) | 0·09(-5·62-6·15) |
| Antigua and Barbuda | both | 0·42(0·36-0·48) | 0·46(0·39-0·54) | 9·13 | 0·78(0·67-0·88) | 0·45(0·39-0·53) | -2·35(-7·98-3·63) |
| Argentina | both | 246·43(217·67-276·78) | 174·37(149·16-202·44) | -29·24 | 0·76(0·67-0·85) | 0·34(0·29-0·4) | -3·51(-9·3-2·65) |
| Armenia | both | 22·17(19·11-26·11) | 17·97(15·11-21) | -18·94 | 0·7(0·6-0·81) | 0·45(0·38-0·52) | -1·63(-7·37-4·45) |
| Australia | both | 330·52(280·4-386·17) | 317·23(254·74-390·64) | -4·02 | 1·68(1·44-1·96) | 0·87(0·69-1·06) | -3·18(-7·03-0·83) |
| Austria | both | 158·21(137·85-183·4) | 147·23(121·62-175·94) | -6·93 | 1·44(1·25-1·67) | 0·93(0·77-1·12) | -1·85(-5·93-2·41) |
| Azerbaijan | both | 17·71(13·63-24·35) | 31·94(23·91-40·82) | 80·36 | 0·29(0·23-0·41) | 0·3(0·23-0·38) | -0·34(-7·97-7·93) |
| Bahrain | both | 1·47(1·05-2·02) | 3·07(2·32-4·26) | 109·41 | 0·61(0·39-0·78) | 0·28(0·21-0·35) | -4·32(-10·62-2·42) |
| Bangladesh | both | 473·16(363·48-621·67) | 566·7(437·74-738·03) | 19·77 | 0·79(0·62-1·06) | 0·43(0·34-0·55) | -2·22(-7·98-3·91) |
| Barbados | both | 2·29(1·98-2·6) | 2·74(2·31-3·22) | 19·56 | 0·8(0·69-0·91) | 0·65(0·55-0·77) | -0·87(-5·99-4·52) |
| Belarus | both | 90·12(74·85-107·56) | 89·87(72·82-112·04) | -0·28 | 0·72(0·61-0·85) | 0·64(0·52-0·79) | -0·18(-5·32-5·23) |
| Belgium | both | 221·12(184·42-262·48) | 161·35(126·65-196·77) | -27·03 | 1·45(1·22-1·7) | 0·7(0·57-0·84) | -2·88(-7·07-1·51) |
| Belize | both | 0·28(0·22-0·34) | 0·82(0·69-0·98) | 192·79 | 0·25(0·2-0·29) | 0·25(0·21-0·3) | -0·12(-8·48-9·01) |
| Benin | both | 13·26(10·43-16·23) | 30·47(22·59-39·52) | 129·76 | 0·53(0·42-0·65) | 0·49(0·37-0·63) | -0·31(-6·43-6·22) |
| Bermuda | both | 0·69(0·57-0·84) | 0·66(0·54-0·82) | -3·97 | 1·04(0·87-1·28) | 0·64(0·52-0·8) | -1·69(-6·76-3·66) |
| Bhutan | both | 2·22(1·75-2·86) | 3·07(2·11-4·1) | 38·3 | 0·69(0·54-0·9) | 0·43(0·3-0·56) | -1·86(-7·81-4·47) |
| Bolivia | both | 21·88(15·83-29·46) | 42·77(29·41-55·57) | 95·51 | 0·55(0·4-0·71) | 0·46(0·32-0·59) | -0·7(-6·81-5·81) |
| Bosnia and Herzegovina | both | 25·54(18·66-37·37) | 23·45(17·98-30·85) | -8·16 | 0·57(0·41-0·84) | 0·45(0·34-0·59) | -1·18(-7·32-5·37) |
| Botswana | both | 0·81(0·48-1·15) | 1·62(0·89-2·14) | 99·46 | 0·12(0·07-0·16) | 0·1(0·06-0·13) | 0·07(-12·89-14·96) |
| Brazil | both | 711·35(680·17-746·04) | 742·26(703·25-791·11) | 4·35 | 0·66(0·63-0·69) | 0·33(0·31-0·35) | -2·83(-8·91-3·66) |
| Brunei | both | 2·9(2·33-3·66) | 6·91(5·61-8·53) | 138·36 | 1·71(1·41-2·33) | 1·72(1·43-2·07) | 0·43(-3·16-4·15) |
| Bulgaria | both | 72·29(63-83·3) | 48·11(40·4-56·51) | -33·45 | 0·61(0·53-0·7) | 0·37(0·3-0·44) | -1·8(-7·48-4·24) |
| Burkina Faso | both | 33·05(25·13-42·65) | 65·53(49·83-84·37) | 98·28 | 0·63(0·48-0·83) | 0·56(0·44-0·71) | -0·5(-6·34-5·7) |
| Burundi | both | 16·77(11·86-25·52) | 23·61(17·58-30·4) | 40·84 | 0·6(0·44-0·91) | 0·46(0·35-0·57) | -1·19(-7·31-5·32) |
| Cambodia | both | 27·41(18·92-54·83) | 50·56(38·28-76·7) | 84·44 | 0·49(0·34-0·97) | 0·4(0·31-0·6) | -0·72(-7·31-6·34) |
| Cameroon | both | 32·16(25·06-39·87) | 80·9(57·31-107·41) | 151·51 | 0·56(0·44-0·69) | 0·53(0·38-0·68) | -0·23(-6·04-5·95) |
| Canada | both | 372·62(322·98-432·38) | 260·83(208·86-320·72) | -30 | 1·15(1-1·33) | 0·45(0·36-0·56) | -4·32(-9·39-1·03) |
| Cape Verde | both | 0·81(0·55-1·27) | 2·4(1·79-3·24) | 195·37 | 0·34(0·23-0·53) | 0·44(0·33-0·6) | 0·84(-6·01-8·19) |
| Central African Republic | both | 9·67(5·94-13·24) | 19·83(12·88-25·93) | 105·03 | 0·65(0·44-0·85) | 0·75(0·5-0·95) | 0·62(-4·64-6·18) |
| Chad | both | 15·71(11·64-20·29) | 36·77(27·36-46·19) | 134·09 | 0·47(0·35-0·61) | 0·52(0·39-0·66) | 0·52(-5·74-7·19) |
| Chile | both | 88·85(80·18-98·03) | 99·67(83·86-117·34) | 12·18 | 0·79(0·72-0·87) | 0·45(0·38-0·53) | -2·13(-7·61-3·68) |
| China | both | 2099·73(1603·68-2638·44) | 2999·47(2533·92-3483·58) | 42·85 | 0·19(0·15-0·23) | 0·16(0·14-0·19) | -1·07(-11·29-10·33) |
| Colombia | both | 184·11(169·35-204·1) | 287·51(242·7-340·51) | 56·16 | 0·84(0·79-0·92) | 0·54(0·45-0·64) | -1·96(-7·13-3·49) |
| Comoros | both | 1·45(1·11-2·03) | 2·53(1·84-3·24) | 74·46 | 0·58(0·45-0·82) | 0·5(0·36-0·64) | -0·55(-6·65-5·94) |
| Congo | both | 8·49(4·94-10·97) | 19·58(12·39-26·77) | 130·61 | 0·64(0·39-0·81) | 0·64(0·42-0·85) | -0·01(-5·54-5·86) |
| Costa Rica | both | 25·57(23·12-28·44) | 51·17(43·66-59·32) | 100·16 | 1·32(1·19-1·45) | 1·04(0·89-1·2) | -0·78(-4·92-3·54) |
| Cote d'Ivoire | both | 29·61(22·3-36·94) | 68·32(52·15-87·85) | 130·76 | 0·49(0·36-0·6) | 0·48(0·37-0·62) | -0·01(-6·24-6·63) |
| Croatia | both | 76·47(63·86-91·52) | 40·71(33·02-50·75) | -46·76 | 1·23(1·03-1·47) | 0·54(0·45-0·67) | -3·06(-7·82-1·94) |
| Cuba | both | 143·83(126·83-160·86) | 139·01(117·09-163·48) | -3·35 | 1·33(1·18-1·49) | 0·82(0·69-0·97) | -1·95(-6·3-2·62) |
| Cyprus | both | 3·3(2·69-4·24) | 4·51(3·57-5·8) | 36·79 | 0·39(0·32-0·5) | 0·25(0·2-0·33) | -1·53(-8·84-6·37) |
| Czech Republic | both | 94·31(82·84-105·16) | 60·76(50·55-72·48) | -35·58 | 0·74(0·65-0·84) | 0·38(0·31-0·46) | -2·62(-8·15-3·25) |
| Democratic Republic of the Congo | both | 94·83(62·51-125·56) | 232·84(164·45-305·97) | 145·53 | 0·48(0·32-0·62) | 0·56(0·39-0·73) | 0·81(-5·39-7·41) |
| Denmark | both | 171·41(148·4-199·2) | 105·26(86·4-127·25) | -38·59 | 2·24(1·92-2·61) | 1·04(0·86-1·25) | -3·14(-6·46-0·29) |
| Djibouti | both | 1·11(0·74-1·53) | 3·21(2·1-4·63) | 189·77 | 0·5(0·35-0·68) | 0·46(0·31-0·65) | -0·41(-6·68-6·29) |
| Dominica | both | 0·5(0·42-0·59) | 0·65(0·55-0·76) | 30·02 | 0·72(0·61-0·85) | 0·77(0·64-0·91) | 0·17(-5·02-5·64) |
| Dominican Republic | both | 8·82(7·09-11·51) | 25·4(19·49-33·38) | 188·08 | 0·19(0·15-0·25) | 0·26(0·2-0·34) | 1·27(-8·39-11·96) |
| Ecuador | both | 20·17(17·31-23·39) | 72·21(61·06-85·46) | 258·04 | 0·3(0·26-0·35) | 0·46(0·39-0·55) | 2·43(-4·97-10·4) |
| Egypt | both | 73·36(49·52-112·45) | 148·37(102·31-239·81) | 102·25 | 0·2(0·13-0·32) | 0·19(0·13-0·33) | -0·08(-9·86-10·77) |
| El Salvador | both | 7·82(6·41-10·26) | 23·52(16·57-30·37) | 200·78 | 0·24(0·19-0·3) | 0·41(0·29-0·52) | 2·65(-5·23-11·18) |
| Equatorial Guinea | both | 1·46(0·89-2·06) | 2·49(1·22-4·07) | 70·68 | 0·62(0·4-0·84) | 0·39(0·2-0·64) | -1·97(-8·33-4·82) |
| Eritrea | both | 9·72(6·39-13·89) | 22·35(16·15-29·64) | 129·93 | 0·75(0·54-1·09) | 0·72(0·54-0·95) | -0·32(-5·32-4·94) |
| Estonia | both | 18·78(15·57-22·47) | 16·72(13·13-21·3) | -10·98 | 0·97(0·8-1·15) | 0·78(0·61-1) | -1·01(-5·58-3·78) |
| Ethiopia | both | 794·33(541·95-1150·55) | 1041·62(740·74-1342·71) | 31·13 | 3·05(2·23-4·16) | 1·98(1·39-2·55) | -1·8(-4·6-1·08) |
| Federated States of Micronesia | both | 0·42(0·31-0·57) | 0·4(0·27-0·55) | -6·28 | 0·7(0·52-0·92) | 0·47(0·33-0·64) | -1·67(-7·49-4·52) |
| Fiji | both | 2·49(1·4-3·26) | 2·5(1·5-3·28) | 0·12 | 0·51(0·29-0·66) | 0·3(0·18-0·39) | -2·41(-9·51-5·24) |
| Finland | both | 101·41(87·3-117·92) | 65·82(54·1-79·12) | -35·09 | 1·48(1·28-1·73) | 0·66(0·55-0·8) | -3·47(-7·65-0·89) |
| France | both | 1250·32(1101·73-1438·31) | 958·89(758·11-1208·85) | -23·31 | 1·55(1·38-1·76) | 0·68(0·56-0·83) | -3·33(-7·37-0·88) |
| Gabon | both | 2·95(1·77-3·95) | 5·95(3·9-8·18) | 101·71 | 0·47(0·28-0·64) | 0·5(0·33-0·69) | 0·46(-5·79-7·12) |
| Georgia | both | 16·16(12·52-19·62) | 18·68(15·64-22·09) | 15·62 | 0·26(0·2-0·32) | 0·39(0·32-0·47) | 1·7(-6·84-11·02) |
| Germany | both | 3032·8(2662·34-3401·92) | 1435·16(1148·15-1761·59) | -52·68 | 2·43(2·14-2·73) | 0·73(0·6-0·87) | -5·3(-9·08--1·36) |
| Ghana | both | 54·52(39·25-69·25) | 94·49(70·81-133·98) | 73·29 | 0·59(0·46-0·73) | 0·47(0·36-0·65) | -1·11(-7·17-5·35) |
| Greece | both | 167·12(142·9-200·19) | 182·29(147·17-230·49) | 9·07 | 1·11(0·96-1·31) | 0·75(0·62-0·92) | -1·89(-6·26-2·69) |
| Greenland | both | 0·52(0·39-0·64) | 0·26(0·2-0·34) | -49·31 | 1·18(0·86-1·43) | 0·41(0·32-0·53) | -4·34(-9·45-1·07) |
| Grenada | both | 0·95(0·8-1·09) | 1·24(1·07-1·42) | 29·8 | 1·35(1·14-1·54) | 0·93(0·8-1·06) | -1·42(-5·85-3·22) |
| Guam | both | 0·48(0·37-0·6) | 0·88(0·71-1·18) | 84·02 | 0·48(0·39-0·6) | 0·49(0·39-0·66) | -0·41(-6·28-5·84) |
| Guatemala | both | 7·48(6·26-9·47) | 34(29·01-39·7) | 354·54 | 0·18(0·15-0·22) | 0·28(0·24-0·33) | 1·27(-7·53-10·91) |
| Guinea | both | 18·51(13·44-22·87) | 35·5(26·74-49·98) | 91·76 | 0·49(0·36-0·61) | 0·54(0·41-0·76) | 0·49(-5·75-7·15) |
| Guinea-Bissau | both | 3·83(2·8-5·36) | 6·51(4·66-8·93) | 70·19 | 0·72(0·54-0·99) | 0·62(0·44-0·82) | -0·49(-5·92-5·26) |
| Guyana | both | 4·21(3·76-4·64) | 5·24(4·42-6·11) | 24·4 | 0·87(0·78-0·95) | 0·78(0·66-0·9) | 0·12(-4·84-5·33) |
| Haiti | both | 40·8(29·12-62·94) | 64·32(45·03-90·31) | 57·65 | 1·04(0·76-1·53) | 0·81(0·58-1·12) | -0·97(-5·59-3·87) |
| Honduras | both | 35·55(27·95-45·48) | 84·96(62·73-115·56) | 138·98 | 1·32(1·03-1·7) | 1·23(0·89-1·65) | -0·36(-4·18-3·61) |
| Hungary | both | 169·46(156·33-183·48) | 56·62(49·01-65·51) | -66·59 | 1·24(1·14-1·35) | 0·36(0·31-0·44) | -5·02(-10·2-0·45) |
| Iceland | both | 4·53(3·74-5·44) | 4·61(3·54-5·69) | 1·77 | 1·56(1·3-1·86) | 0·92(0·71-1·14) | -1·27(-5·08-2·7) |
| India | both | 3473·01(2895·87-4394·58) | 6403·43(5413·41-7369·36) | 84·38 | 0·62(0·52-0·78) | 0·56(0·47-0·64) | -0·47(-6·22-5·64) |
| Indonesia | both | 392·07(307·77-654·84) | 784·88(595·42-1095·85) | 100·19 | 0·31(0·25-0·53) | 0·33(0·26-0·47) | 0·34(-7·21-8·52) |
| Iran | both | 242·65(128·58-304·44) | 321·21(246·69-382·85) | 32·38 | 0·71(0·38-0·88) | 0·4(0·31-0·48) | -1·79(-7·73-4·53) |
| Iraq | both | 50·48(36·31-74·21) | 65·58(50·33-91·43) | 29·92 | 0·51(0·38-0·75) | 0·22(0·17-0·3) | -3·23(-10·11-4·18) |
| Ireland | both | 51·88(45·65-60·05) | 36·73(29·71-45·46) | -29·22 | 1·29(1·13-1·47) | 0·53(0·43-0·66) | -3·07(-7·64-1·72) |
| Israel | both | 55·79(48·93-64·07) | 43·67(36·18-54·35) | -21·72 | 1·17(1·03-1·34) | 0·39(0·32-0·47) | -4·49(-9·42-0·72) |
| Italy | both | 1380·99(1191·07-1599·16) | 1016·86(801·85-1267·01) | -26·37 | 1·65(1·44-1·89) | 0·69(0·57-0·84) | -3·38(-7·51-0·94) |
| Jamaica | both | 6·19(5·33-7·07) | 19·43(14·77-25·29) | 214 | 0·34(0·29-0·39) | 0·66(0·51-0·86) | 2·89(-3·66-9·88) |
| Japan | both | 1456·42(1321·68-1600·02) | 986·35(851·04-1161·87) | -32·28 | 0·94(0·85-1·04) | 0·4(0·34-0·47) | -3·54(-8·96-2·21) |
| Jordan | both | 3·65(2·52-4·96) | 9·09(6·67-12·13) | 149·02 | 0·18(0·12-0·24) | 0·12(0·09-0·15) | -2·13(-13·12-10·25) |
| Kazakhstan | both | 97·03(76·92-126·94) | 71·44(54-96·23) | -26·38 | 0·65(0·52-0·84) | 0·39(0·29-0·51) | -1·85(-7·55-4·2) |
| Kenya | both | 18·73(14·24-27·08) | 46·04(37·1-57·99) | 145·85 | 0·17(0·13-0·26) | 0·17(0·14-0·22) | -0·05(-10·23-11·28) |
| Kiribati | both | 0·33(0·25-0·42) | 0·52(0·38-0·67) | 55·05 | 0·69(0·51-0·86) | 0·61(0·44-0·8) | -0·35(-5·87-5·5) |
| Kuwait | both | 3·32(2·68-4·11) | 8·05(6·17-10·64) | 142·68 | 0·27(0·23-0·32) | 0·2(0·16-0·25) | -0·7(-9·71-9·2) |
| Kyrgyzstan | both | 13·3(11·37-15·55) | 10·93(9·11-13·01) | -17·83 | 0·39(0·33-0·45) | 0·21(0·18-0·24) | -1·99(-9·57-6·23) |
| Laos | both | 11·91(7·72-23·18) | 18·21(13·01-26·51) | 52·87 | 0·47(0·31-0·93) | 0·36(0·26-0·52) | -1(-7·72-6·22) |
| Latvia | both | 30·35(24·85-37·79) | 23·42(18·78-28·81) | -22·84 | 0·86(0·7-1·05) | 0·68(0·55-0·84) | -0·98(-5·89-4·19) |
| Lebanon | both | 10·5(6·77-14·82) | 31·06(21·24-43·97) | 195·73 | 0·4(0·25-0·55) | 0·42(0·29-0·58) | 0·32(-6·83-8·02) |
| Lesotho | both | 1·43(0·94-1·93) | 2·42(1·63-3·37) | 69·2 | 0·13(0·08-0·17) | 0·18(0·12-0·24) | 1·76(-8·91-13·68) |
| Liberia | both | 6·21(4·7-7·85) | 12·11(9·2-15·43) | 94·9 | 0·5(0·38-0·64) | 0·46(0·35-0·58) | -0·49(-6·87-6·31) |
| Libya | both | 7·59(5·23-10·36) | 22·67(16·27-30·81) | 198·67 | 0·32(0·22-0·44) | 0·38(0·27-0·49) | 0·73(-6·72-8·78) |
| Lithuania | both | 60·86(52·12-70·04) | 33·38(27·2-41·53) | -45·15 | 1·37(1·18-1·57) | 0·73(0·59-0·91) | -2·27(-6·37-2·02) |
| Luxembourg | both | 7·23(5·82-9·05) | 5·78(4·52-7·22) | -20·03 | 1·35(1·1-1·67) | 0·61(0·48-0·77) | -3·55(-8·01-1·12) |
| Macedonia | both | 10·24(7·93-12·69) | 10·5(8·13-13·67) | 2·6 | 0·51(0·41-0·64) | 0·35(0·27-0·45) | -1·75(-8·36-5·34) |
| Madagascar | both | 33·8(26·55-46·44) | 61·88(46·48-78·85) | 83·08 | 0·5(0·4-0·68) | 0·45(0·34-0·58) | -0·31(-6·78-6·61) |
| Malawi | both | 17·74(8·86-24·52) | 27·98(20·21-35·1) | 57·7 | 0·37(0·21-0·49) | 0·32(0·22-0·39) | -1·02(-8·27-6·8) |
| Malaysia | both | 77·41(61·77-97·72) | 193·91(137·1-279·35) | 150·51 | 0·69(0·55-0·95) | 0·7(0·5-1·05) | 0·06(-5·37-5·8) |
| Maldives | both | 1·16(0·62-2·33) | 2·29(1·74-3·07) | 96·49 | 0·93(0·53-1·74) | 0·6(0·47-0·76) | -1·85(-6·98-3·56) |
| Mali | both | 23(17·91-28·97) | 38·92(28·08-52·84) | 69·22 | 0·47(0·37-0·59) | 0·39(0·28-0·52) | -0·77(-7·6-6·56) |
| Malta | both | 5·17(4·49-6) | 5·22(4·24-6·44) | 0·89 | 1·23(1·07-1·42) | 0·63(0·52-0·78) | -2·97(-7·6-1·9) |
| Marshall Islands | both | 0·15(0·11-0·21) | 0·23(0·15-0·33) | 47 | 0·64(0·48-0·85) | 0·51(0·34-0·73) | -0·92(-6·63-5·14) |
| Mauritania | both | 6·72(5·22-8·53) | 11·62(8·29-15·06) | 73·08 | 0·55(0·42-0·69) | 0·49(0·36-0·63) | -0·3(-6·51-6·31) |
| Mauritius | both | 2·15(1·88-2·42) | 5·14(4·25-6·2) | 138·89 | 0·26(0·23-0·29) | 0·31(0·26-0·37) | 0·7(-8·02-10·25) |
| Mexico | both | 298·04(286·53-312·42) | 511·47(479·79-546·42) | 71·61 | 0·54(0·52-0·57) | 0·42(0·4-0·45) | -1·38(-7·44-5·07) |
| Moldova | both | 11·66(9·44-14·06) | 7·42(6·14-9·11) | -36·35 | 0·25(0·2-0·3) | 0·14(0·12-0·18) | -2(-11·6-8·65) |
| Mongolia | both | 4·82(3·81-6·26) | 7·98(6·08-11·36) | 65·66 | 0·38(0·31-0·49) | 0·29(0·23-0·39) | -1·39(-8·58-6·36) |
| Montenegro | both | 4·96(3·96-6·24) | 4·83(3·8-6·4) | -2·67 | 0·77(0·62-0·97) | 0·55(0·43-0·74) | -1·38(-6·63-4·17) |
| Morocco | both | 39·3(18·62-52·54) | 60·57(34·76-82·84) | 54·12 | 0·23(0·1-0·31) | 0·18(0·1-0·24) | -1·02(-10·45-9·41) |
| Mozambique | both | 47·25(33·89-79·63) | 76·47(55·31-135·18) | 61·86 | 0·66(0·49-1·05) | 0·58(0·44-0·92) | -0·5(-6·25-5·61) |
| Myanmar | both | 178·92(112·07-378·86) | 209·54(151·94-310·07) | 17·12 | 0·64(0·41-1·39) | 0·43(0·31-0·64) | -1·4(-7·29-4·88) |
| Namibia | both | 0·93(0·57-1·31) | 1·3(0·81-1·85) | 39·4 | 0·11(0·07-0·15) | 0·08(0·05-0·12) | -1·39(-14·14-13·26) |
| Nepal | both | 86·71(64·16-121·44) | 126·02(95·26-168·47) | 45·33 | 0·76(0·57-1·06) | 0·56(0·42-0·74) | -1·2(-6·8-4·74) |
| Netherlands | both | 284·81(237·95-333·93) | 200·07(155·96-252·34) | -29·75 | 1·44(1·22-1·69) | 0·63(0·5-0·78) | -2·98(-7·31-1·56) |
| New Zealand | both | 56·19(46·42-68·38) | 40·74(32·24-50·99) | -27·5 | 1·42(1·18-1·72) | 0·63(0·5-0·78) | -3·76(-8·24-0·92) |
| Nicaragua | both | 10·35(8·39-12·19) | 22·38(17·18-27·71) | 116·15 | 0·46(0·39-0·55) | 0·42(0·32-0·51) | -0·19(-6·62-6·68) |
| Niger | both | 19·95(14·77-25·68) | 46·76(33·61-61·12) | 134·37 | 0·5(0·38-0·65) | 0·48(0·35-0·62) | -0·31(-6·6-6·4) |
| Nigeria | both | 170·25(110·42-268·03) | 340·53(207·47-534·69) | 100·01 | 0·32(0·21-0·5) | 0·32(0·2-0·49) | -0·01(-7·76-8·4) |
| North Korea | both | 48·79(35·82-64·62) | 68·69(49·91-98·63) | 40·79 | 0·25(0·18-0·32) | 0·23(0·16-0·32) | -0·48(-9·24-9·13) |
| Northern Mariana Islands | both | 0·2(0·13-0·27) | 0·2(0·15-0·3) | 1·45 | 0·62(0·43-0·8) | 0·37(0·28-0·54) | -2·56(-8·52-3·79) |
| Norway | both | 68·71(60·13-78·32) | 54·25(46·87-62·5) | -21·05 | 1·11(0·97-1·27) | 0·63(0·55-0·74) | -2·24(-6·83-2·57) |
| Oman | both | 2·5(1·71-3·37) | 6·67(4·44-9·5) | 166·85 | 0·24(0·16-0·32) | 0·19(0·12-0·26) | -0·76(-9·91-9·32) |
| Pakistan | both | 461·94(334·57-609·83) | 944·72(637·69-1295·93) | 104·51 | 0·67(0·49-0·89) | 0·66(0·46-0·89) | -0·14(-5·39-5·4) |
| Palestine | both | 2·9(2·07-4·45) | 7·28(5·32-10·41) | 151·52 | 0·25(0·18-0·39) | 0·22(0·16-0·31) | -0·51(-9·36-9·2) |
| Panama | both | 11·98(10·47-13·51) | 17·97(15·39-20·98) | 49·98 | 0·7(0·62-0·78) | 0·45(0·39-0·53) | -1·58(-7·61-4·83) |
| Papua New Guinea | both | 24·01(17·03-33·92) | 43·34(29·79-63·72) | 80·52 | 0·88(0·65-1·19) | 0·65(0·47-0·91) | -1·11(-6·18-4·24) |
| Paraguay | both | 8·3(6·58-10·44) | 23·14(17·74-30·03) | 178·76 | 0·33(0·27-0·41) | 0·4(0·31-0·52) | 1·27(-5·81-8·88) |
| Peru | both | 44·92(35·19-55·58) | 92·58(65·31-117·85) | 106·08 | 0·29(0·23-0·36) | 0·29(0·2-0·36) | -0·14(-8·06-8·46) |
| Philippines | both | 111·02(96·13-129·63) | 364·99(298·1-438·89) | 228·75 | 0·26(0·23-0·3) | 0·42(0·35-0·5) | 2·05(-5·53-10·24) |
| Poland | both | 420·65(375·11-465·96) | 229·01(199·93-258·93) | -45·56 | 0·95(0·85-1·05) | 0·37(0·32-0·43) | -3·61(-8·88-1·97) |
| Portugal | both | 124·89(115·93-134·67) | 89·7(77·66-104·49) | -28·17 | 0·97(0·9-1·06) | 0·44(0·37-0·52) | -3·74(-8·8-1·61) |
| Puerto Rico | both | 31·51(27·85-35·41) | 19·15(16·14-23·36) | -39·21 | 0·86(0·76-0·96) | 0·34(0·29-0·42) | -4·34(-10·22-1·92) |
| Qatar | both | 1·19(0·69-1·71) | 6·06(3·57-12·26) | 407·68 | 0·64(0·36-0·9) | 0·38(0·27-0·54) | -2·58(-8·72-3·97) |
| Romania | both | 103·93(90·18-119·2) | 76·93(66·44-88·34) | -25·98 | 0·38(0·33-0·44) | 0·25(0·22-0·3) | -1·6(-8·96-6·36) |
| Russian Federation | both | 1020·98(887·09-1176·9) | 1200·18(1026·5-1394·07) | 17·55 | 0·57(0·49-0·65) | 0·57(0·49-0·67) | -0·02(-5·53-5·81) |
| Rwanda | both | 22·44(16·51-36·47) | 29·26(21·67-37·47) | 30·36 | 0·61(0·46-0·99) | 0·43(0·32-0·54) | -1·72(-7·72-4·66) |
| Saint Lucia | both | 1·1(0·98-1·23) | 1·37(1·19-1·59) | 24·51 | 1·12(1-1·26) | 0·67(0·57-0·77) | -2·34(-7·18-2·74) |
| Saint Vincent and the Grenadines | both | 1·44(1·28-1·59) | 0·93(0·81-1·07) | -34·94 | 1·79(1·6-1·98) | 0·72(0·62-0·83) | -3·92(-8·38-0·76) |
| Samoa | both | 0·43(0·32-0·6) | 0·48(0·34-0·67) | 10·68 | 0·42(0·32-0·59) | 0·32(0·23-0·45) | -1·2(-8·41-6·57) |
| Sao Tome and Principe | both | 0·29(0·21-0·38) | 0·6(0·41-0·85) | 108·9 | 0·38(0·29-0·51) | 0·43(0·3-0·62) | 0·35(-6·41-7·59) |
| Saudi Arabia | both | 36·11(24·35-48·81) | 122·21(88·83-164·05) | 238·44 | 0·5(0·33-0·68) | 0·55(0·42-0·69) | 0·47(-5·71-7·06) |
| Senegal | both | 21·88(17·35-27·17) | 47·38(36·36-60·75) | 116·59 | 0·54(0·44-0·68) | 0·55(0·42-0·69) | 0·1(-5·95-6·54) |
| Serbia | both | 34·56(26·87-43·05) | 47·03(32·98-59·41) | 36·1 | 0·33(0·25-0·41) | 0·34(0·24-0·43) | 0·94(-6·39-8·84) |
| Seychelles | both | 0·64(0·53-0·9) | 1·32(0·94-1·64) | 105·81 | 1·06(0·87-1·52) | 1·2(0·87-1·49) | 0·32(-3·77-4·59) |
| Sierra Leone | both | 12·4(9·67-16·22) | 24·51(18·63-31·68) | 97·7 | 0·53(0·42-0·69) | 0·54(0·42-0·69) | 0·24(-5·78-6·65) |
| Singapore | both | 20·1(17·38-22·81) | 22·29(17·67-27·71) | 10·87 | 0·68(0·6-0·76) | 0·32(0·25-0·4) | -2·75(-8·94-3·87) |
| Slovakia | both | 61·04(47·72-77·56) | 35·67(26·09-45·68) | -41·57 | 1·02(0·8-1·3) | 0·42(0·31-0·54) | -2·87(-8·06-2·61) |
| Slovenia | both | 26·15(23·31-29·79) | 35·38(29·58-42·44) | 35·27 | 1·09(0·97-1·24) | 0·96(0·81-1·17) | 0·05(-4·29-4·58) |
| Solomon Islands | both | 1·37(0·98-1·96) | 2·18(1·55-3·08) | 58·67 | 0·71(0·52-0·98) | 0·51(0·38-0·7) | -1·23(-6·89-4·77) |
| Somalia | both | 18·27(9·15-29·74) | 49·86(33·84-69·25) | 172·86 | 0·55(0·32-0·85) | 0·63(0·44-0·86) | 0·3(-5·4-6·35) |
| South Africa | both | 23·19(17·83-28·33) | 28·16(23·44-36·67) | 21·47 | 0·09(0·07-0·11) | 0·06(0·05-0·07) | -1·65(-16·23-15·47) |
| South Korea | both | 229·02(196·06-270·06) | 265·3(205·09-331·55) | 15·84 | 0·52(0·45-0·61) | 0·35(0·27-0·43) | -0·48(-6·88-6·36) |
| South Sudan | both | 12·65(6·77-20·17) | 22·73(15·3-32·49) | 79·65 | 0·43(0·26-0·64) | 0·48(0·32-0·67) | 0·28(-6·33-7·36) |
| Spain | both | 864·38(786·27-934) | 629·42(518·01-753·97) | -27·18 | 1·68(1·52-1·83) | 0·7(0·59-0·84) | -4(-7·99-0·16) |
| Sri Lanka | both | 57·75(46·98-73·53) | 87·4(64·14-115·83) | 51·35 | 0·42(0·34-0·54) | 0·36(0·27-0·48) | -0·52(-7·6-7·1) |
| Sudan | both | 58·76(30·65-91·11) | 87·27(48·64-120·29) | 48·51 | 0·5(0·25-0·73) | 0·38(0·2-0·51) | -1·15(-7·78-5·96) |
| Suriname | both | 1·42(1·22-1·62) | 2·22(1·87-2·59) | 56·2 | 0·48(0·41-0·54) | 0·37(0·32-0·44) | -1·07(-7·76-6·11) |
| Swaziland | both | 0·5(0·33-0·66) | 0·92(0·57-1·26) | 82·5 | 0·13(0·09-0·17) | 0·13(0·08-0·18) | 0·58(-10·47-12·98) |
| Sweden | both | 124·58(109·48-143·99) | 84·75(71·55-99·85) | -31·97 | 0·97(0·83-1·14) | 0·49(0·4-0·58) | -3·01(-8·36-2·66) |
| Switzerland | both | 149·72(126·5-179·5) | 113·52(93·99-134·91) | -24·18 | 1·45(1·22-1·73) | 0·7(0·58-0·84) | -3·25(-7·22-0·9) |
| Syria | both | 66·25(44·77-101·31) | 100·65(71·95-152·25) | 51·94 | 1·04(0·69-1·57) | 0·71(0·51-1·06) | -1·98(-6·63-2·89) |
| Tajikistan | both | 16·12(11·86-21·14) | 17·74(13·1-26·2) | 10 | 0·46(0·34-0·61) | 0·25(0·19-0·36) | -2·62(-10·19-5·59) |
| Tanzania | both | 61·36(41·6-83·79) | 123·82(85·84-157·62) | 101·77 | 0·46(0·33-0·62) | 0·43(0·3-0·54) | -0·52(-7·12-6·56) |
| Thailand | both | 294·47(210·14-356·99) | 596·01(380·1-764·54) | 102·4 | 0·66(0·48-0·8) | 0·65(0·41-0·83) | -0·5(-5·92-5·22) |
| The Bahamas | both | 1·93(1·68-2·19) | 3·27(2·75-3·86) | 69·12 | 1(0·88-1·12) | 0·82(0·7-0·97) | -0·62(-5·36-4·35) |
| The Gambia | both | 2·39(1·78-3·24) | 6·18(4·51-8·57) | 158·85 | 0·48(0·37-0·65) | 0·52(0·39-0·7) | 0·49(-5·76-7·16) |
| Timor-Leste | both | 1·49(0·96-2·51) | 3·09(2·01-4·48) | 107·87 | 0·35(0·24-0·61) | 0·36(0·24-0·51) | 0·13(-7·33-8·2) |
| Togo | both | 9·6(7·47-12) | 22·69(16·58-30·22) | 136·38 | 0·53(0·42-0·67) | 0·49(0·37-0·63) | -0·38(-6·45-6·09) |
| Tonga | both | 0·21(0·16-0·26) | 0·21(0·17-0·27) | 3·81 | 0·33(0·26-0·41) | 0·25(0·2-0·32) | -1·05(-9·2-7·82) |
| Trinidad and Tobago | both | 8·44(7·43-9·48) | 8·92(6·98-11·44) | 5·68 | 0·87(0·77-0·98) | 0·51(0·4-0·66) | -2·38(-7·73-3·27) |
| Tunisia | both | 16·07(11·44-23·48) | 30·88(22·26-42) | 92·19 | 0·29(0·2-0·42) | 0·25(0·18-0·34) | -0·69(-8·91-8·28) |
| Turkey | both | 352·18(268·12-443·61) | 321·08(262·37-428·6) | -8·83 | 0·87(0·66-1·08) | 0·37(0·3-0·49) | -3·33(-9·01-2·71) |
| Turkmenistan | both | 8·84(6·41-11·43) | 12·1(8·64-15·7) | 36·84 | 0·35(0·26-0·45) | 0·27(0·19-0·34) | -1·2(-8·93-7·19) |
| Uganda | both | 31·07(24·05-38·96) | 65·46(50·07-81·61) | 110·7 | 0·38(0·3-0·47) | 0·38(0·29-0·47) | -0·14(-7·06-7·29) |
| Ukraine | both | 568·75(471·14-698·77) | 480·35(400·58-579·37) | -15·54 | 0·86(0·71-1·05) | 0·75(0·62-0·9) | -1·56(-6·62-3·76) |
| United Arab Emirates | both | 3·71(1·64-5·57) | 31·53(17·22-47·1) | 749·21 | 0·51(0·18-0·8) | 0·47(0·2-0·79) | -0·36(-6·61-6·31) |
| United Kingdom | both | 1711·3(1605·64-1840·89) | 809·47(765·35-866·66) | -52·7 | 1·97(1·86-2·12) | 0·69(0·65-0·74) | -5·1(-9·17--0·84) |
| United States | both | 3037·19(2935·11-3151·91) | 1793·68(1700·65-1912·88) | -40·94 | 1·01(0·97-1·05) | 0·39(0·37-0·42) | -4·32(-9·73-1·42) |
| Uruguay | both | 41·82(37·94-46·28) | 25·97(22·11-30·9) | -37·91 | 1·16(1·05-1·28) | 0·55(0·46-0·66) | -3·05(-7·83-1·97) |
| Uzbekistan | both | 55·76(42·5-68·62) | 85·13(65·29-107·21) | 52·66 | 0·38(0·29-0·47) | 0·31(0·24-0·38) | -0·62(-7·83-7·16) |
| Vanuatu | both | 0·77(0·41-1·24) | 1·35(0·67-2·32) | 75·09 | 0·83(0·46-1·34) | 0·66(0·33-1·11) | -0·96(-6·03-4·38) |
| Venezuela | both | 117·11(102·99-132·57) | 163·04(131·41-202·49) | 39·23 | 0·95(0·85-1·07) | 0·55(0·45-0·68) | -2·31(-7·45-3·11) |
| Vietnam | both | 82·76(66·15-109·14) | 196·68(136·67-251·54) | 137·66 | 0·19(0·15-0·25) | 0·21(0·14-0·26) | 0·6(-9·34-11·64) |
| Virgin Islands, U.S. | both | 0·54(0·46-0·63) | 0·56(0·46-0·67) | 3·55 | 0·59(0·51-0·69) | 0·36(0·29-0·44) | -2·06(-8·74-5·1) |
| Yemen | both | 41·17(19·29-66·05) | 90·92(49·92-133·88) | 120·87 | 0·62(0·3-0·98) | 0·53(0·28-0·76) | -0·69(-6·45-5·42) |
| Zambia | both | 23·18(15·64-32·5) | 41·17(29·36-53·83) | 77·61 | 0·62(0·44-0·86) | 0·49(0·36-0·63) | -1·23(-7·08-4·99) |
| Zimbabwe | both | 6·21(3·6-10·07) | 14·08(7·98-18·82) | 126·65 | 0·12(0·08-0·2) | 0·17(0·11-0·23) | 1·93(-8·76-13·88) |

ASIR, age-standardized incidence rate; EAPC, estimated annual percentage change.

**Supplementary table S12. Death cases and ASDR by countries in 1990 and 2017 and its change trends from 1990 to 2017.**

| Nation | Sex | death cases  No.×10^2^  (95% UI) | | Change (%) | ASDR  per 100,000  No.(95% UI) | | EAPC |
| --- | --- | --- | --- | --- | --- | --- | --- |
|  |  | 1990 | 2017 |  | 1990 | 2017 |  |
| Afghanistan | both | 59(24·5-108·44) | 110·09(53·12-187·86) | 86·59 | 0·84(0·36-1·53) | 0·74(0·34-1·16) | -0·64(4·41--5·45) |
| Albania | both | 9·15(6·36-15·05) | 10·66(6·36-20·53) | 16·47 | 0·37(0·25-0·65) | 0·28(0·17-0·51) | -1·27(6·91--8·83) |
| Algeria | both | 86·39(69·11-105·45) | 153·95(118·71-180·15) | 78·21 | 0·62(0·5-0·78) | 0·45(0·35-0·53) | -0·95(5·37--6·88) |
| American Samoa | both | 0·12(0·09-0·14) | 0·11(0·09-0·14) | -0·99 | 0·42(0·32-0·5) | 0·24(0·2-0·3) | -2·44(5·77--10·01) |
| Andorra | both | 0·41(0·32-0·51) | 0·52(0·41-0·65) | 25·87 | 0·71(0·56-0·88) | 0·37(0·29-0·47) | -2·74(3·82--8·88) |
| Angola | both | 26·69(16·35-36·39) | 64·53(42·77-87·58) | 141·8 | 0·55(0·36-0·73) | 0·53(0·35-0·72) | 0·01(6·3--5·91) |
| Antigua and Barbuda | both | 0·34(0·29-0·38) | 0·31(0·27-0·35) | -7·44 | 0·63(0·55-0·72) | 0·31(0·27-0·36) | -2·96(3·96--9·42) |
| Argentina | both | 212·91(190·05-236·11) | 136·77(118·05-155·54) | -35·76 | 0·66(0·59-0·73) | 0·26(0·22-0·29) | -3·95(2·92--10·36) |
| Armenia | both | 18·3(15·64-21·14) | 15·33(13·43-17·43) | -16·22 | 0·59(0·51-0·68) | 0·37(0·32-0·42) | -1·75(4·99--8·06) |
| Australia | both | 243·92(232·54-257·02) | 186·05(161·25-215·08) | -23·73 | 1·23(1·18-1·3) | 0·45(0·39-0·52) | -4·51(0·69--9·45) |
| Austria | both | 118·51(110·56-126·05) | 94·29(84·13-105·44) | -20·44 | 1·01(0·95-1·07) | 0·51(0·46-0·57) | -2·55(2·83--7·64) |
| Azerbaijan | both | 13·77(10·63-19·02) | 21·41(16·47-26·76) | 55·46 | 0·24(0·19-0·34) | 0·22(0·16-0·27) | -0·93(8·53--9·56) |
| Bahrain | both | 1·17(0·82-1·55) | 1·89(1·48-2·49) | 61·26 | 0·58(0·35-0·74) | 0·21(0·16-0·28) | -5·07(2·2--11·82) |
| Bangladesh | both | 418·07(324·94-555·85) | 464·06(368·86-589·85) | 11 | 0·76(0·59-1·04) | 0·37(0·3-0·47) | -2·61(3·75--8·58) |
| Barbados | both | 1·97(1·7-2·24) | 2·14(1·84-2·5) | 8·87 | 0·67(0·58-0·75) | 0·47(0·41-0·55) | -1·37(4·86--7·22) |
| Belarus | both | 73·57(61·29-85·55) | 62·1(53·75-71·4) | -15·58 | 0·57(0·48-0·66) | 0·4(0·35-0·46) | -0·91(5·41--6·85) |
| Belgium | both | 161·15(147·93-173·87) | 113·84(101·63-126·86) | -29·36 | 1·03(0·95-1·1) | 0·46(0·41-0·5) | -3·19(2·1--8·21) |
| Belize | both | 0·22(0·17-0·26) | 0·53(0·46-0·6) | 142·06 | 0·2(0·16-0·24) | 0·18(0·15-0·2) | -0·71(9·85--10·25) |
| Benin | both | 11·54(9·23-14·26) | 24·93(18·63-31·84) | 116·08 | 0·49(0·39-0·61) | 0·44(0·33-0·57) | -0·43(6·42--6·84) |
| Bermuda | both | 0·57(0·47-0·68) | 0·52(0·44-0·6) | -8·51 | 0·87(0·73-1·05) | 0·45(0·38-0·52) | -2·39(3·75--8·18) |
| Bhutan | both | 1·91(1·5-2·47) | 2·45(1·72-3·23) | 28·53 | 0·66(0·51-0·85) | 0·38(0·27-0·5) | -2·1(4·46--8·26) |
| Bolivia | both | 18·35(13·35-24·22) | 33·41(23·59-43·88) | 82·04 | 0·5(0·36-0·63) | 0·38(0·27-0·5) | -1·04(5·98--7·59) |
| Bosnia and Herzegovina | both | 22·14(16·03-32·98) | 22·97(17·97-29·97) | 3·74 | 0·51(0·37-0·78) | 0·4(0·31-0·52) | -1·16(5·8--7·66) |
| Botswana | both | 0·7(0·43-0·97) | 1·21(0·69-1·55) | 73·6 | 0·11(0·07-0·14) | 0·08(0·05-0·11) | -0·34(15·7--14·16) |
| Brazil | both | 573·13(551·21-596·46) | 578·86(556·6-609·78) | 1 | 0·57(0·55-0·59) | 0·26(0·25-0·27) | -3·14(4·04--9·82) |
| Brunei | both | 2·07(1·67-2·68) | 3·88(3·25-4·64) | 86·95 | 1·4(1·14-1·94) | 1·09(0·93-1·31) | -0·7(3·69--4·89) |
| Bulgaria | both | 62·62(55·15-72·07) | 42·91(37·68-48·99) | -31·48 | 0·51(0·45-0·59) | 0·29(0·26-0·33) | -1·88(5·09--8·38) |
| Burkina Faso | both | 27·81(21·43-36·29) | 51·18(38·83-65·14) | 84·06 | 0·57(0·44-0·75) | 0·49(0·38-0·61) | -0·64(5·96--6·83) |
| Burundi | both | 14·88(10·55-22·85) | 20·31(15·44-26·18) | 36·52 | 0·58(0·43-0·89) | 0·44(0·34-0·56) | -1·22(5·41--7·43) |
| Cambodia | both | 23·21(15·98-46·29) | 39·67(30·49-61·42) | 70·91 | 0·44(0·31-0·88) | 0·33(0·26-0·52) | -1·02(6·5--8·01) |
| Cameroon | both | 27·32(21·29-33·8) | 65·51(46·72-85·52) | 139·74 | 0·52(0·41-0·64) | 0·48(0·34-0·62) | -0·27(6·21--6·36) |
| Canada | both | 262·31(246·88-277·43) | 170·4(150·87-192·44) | -35·04 | 0·8(0·76-0·85) | 0·26(0·23-0·29) | -5·14(1·7--11·51) |
| Cape Verde | both | 0·67(0·46-1·05) | 1·78(1·36-2·34) | 165·77 | 0·28(0·19-0·44) | 0·34(0·26-0·45) | 0·49(8·64--7·05) |
| Central African Republic | both | 8·38(5·38-11·27) | 17·43(11·49-22·69) | 108·04 | 0·61(0·43-0·8) | 0·71(0·49-0·92) | 0·68(6·41--4·74) |
| Chad | both | 13·84(10·34-18·02) | 30·92(23·02-38·88) | 123·37 | 0·44(0·33-0·57) | 0·48(0·36-0·61) | 0·49(7·45--6·02) |
| Chile | both | 71·51(65·8-77·41) | 70·22(60·57-80·61) | -1·8 | 0·67(0·61-0·72) | 0·31(0·27-0·36) | -2·83(3·78--9·02) |
| China | both | 1393·4(1101·4-1744·72) | 1069·83(895·86-1269·9) | -23·22 | 0·13(0·11-0·17) | 0·06(0·05-0·07) | -3·62(12·18--17·2) |
| Colombia | both | 147·95(138·37-157·34) | 216·9(187·85-246·68) | 46·6 | 0·74(0·69-0·79) | 0·4(0·35-0·46) | -2·52(3·54--8·22) |
| Comoros | both | 1·28(0·99-1·8) | 2·2(1·63-2·84) | 71·91 | 0·55(0·43-0·79) | 0·46(0·34-0·61) | -0·68(6·01--6·96) |
| Congo | both | 7·49(4·49-9·65) | 16·3(10·5-21·83) | 117·7 | 0·61(0·39-0·76) | 0·59(0·4-0·79) | -0·09(5·97--5·81) |
| Costa Rica | both | 21·58(19·57-23·65) | 39·68(34·37-45·07) | 83·91 | 1·18(1·07-1·29) | 0·82(0·71-0·93) | -1·25(3·45--5·73) |
| Cote d'Ivoire | both | 24·06(17·91-30·02) | 55·38(42·46-72·16) | 130·14 | 0·45(0·34-0·56) | 0·44(0·34-0·57) | -0·06(6·88--6·55) |
| Croatia | both | 56·89(50·39-64·18) | 28·81(25·51-33·12) | -49·35 | 0·89(0·79-1) | 0·33(0·3-0·37) | -3·66(2·59--9·53) |
| Cuba | both | 122·82(109·33-134·92) | 117·03(102·04-133·69) | -4·71 | 1·16(1·03-1·27) | 0·65(0·57-0·74) | -2·24(2·78--7·01) |
| Cyprus | both | 2·73(2·24-3·69) | 2·89(2·41-3·65) | 6·23 | 0·33(0·27-0·44) | 0·16(0·13-0·2) | -2·72(6·62--11·24) |
| Czech Republic | both | 80·58(74·03-86·55) | 57·53(51·48-64·38) | -28·6 | 0·61(0·56-0·65) | 0·3(0·27-0·33) | -2·86(3·68--8·98) |
| Democratic Republic of the Congo | both | 81·88(54·07-107·65) | 197·36(139·68-255·62) | 141·04 | 0·45(0·31-0·58) | 0·53(0·36-0·68) | 0·8(7·6--5·58) |
| Denmark | both | 121·67(113·37-130·03) | 70·29(62·6-78·37) | -42·23 | 1·48(1·39-1·58) | 0·62(0·56-0·69) | -3·42(0·92--7·57) |
| Djibouti | both | 0·94(0·64-1·3) | 2·67(1·76-3·82) | 183·88 | 0·48(0·34-0·65) | 0·43(0·29-0·61) | -0·54(6·32--6·95) |
| Dominica | both | 0·42(0·35-0·5) | 0·51(0·44-0·6) | 20·05 | 0·6(0·5-0·7) | 0·58(0·49-0·67) | -0·26(5·94--6·1) |
| Dominican Republic | both | 7·02(5·73-9·28) | 17·48(13·5-22·32) | 148·92 | 0·16(0·13-0·22) | 0·18(0·14-0·24) | 0·6(12·77--10·26) |
| Ecuador | both | 16·37(14·34-18·9) | 50·11(43·66-56·58) | 206·1 | 0·26(0·23-0·3) | 0·34(0·29-0·38) | 1·73(10·77--6·58) |
| Egypt | both | 55·83(37·33-88·59) | 87·91(61·72-147·78) | 57·45 | 0·16(0·1-0·28) | 0·13(0·09-0·22) | -0·94(11·66--12·12) |
| El Salvador | both | 6·58(5·43-8·39) | 17·11(11·43-21·55) | 159·99 | 0·21(0·17-0·26) | 0·3(0·2-0·38) | 2·02(11·61--6·75) |
| Equatorial Guinea | both | 1·3(0·81-1·81) | 1·89(0·95-3·12) | 45·21 | 0·59(0·39-0·79) | 0·35(0·18-0·57) | -2·29(4·78--8·88) |
| Eritrea | both | 8·62(5·73-12·17) | 18·53(13·61-24·13) | 114·86 | 0·73(0·54-1·08) | 0·68(0·5-0·89) | -0·36(5·07--5·51) |
| Estonia | both | 14·92(12·84-17·07) | 10·32(8·73-12·13) | -30·84 | 0·75(0·64-0·85) | 0·4(0·34-0·46) | -2·54(3·35--8·09) |
| Ethiopia | both | 694·38(491·48-981·87) | 892·84(628·58-1150·86) | 28·58 | 2·94(2·21-3·92) | 1·89(1·33-2·48) | -1·82(1·11--4·68) |
| Federated States of Micronesia | both | 0·36(0·27-0·48) | 0·31(0·21-0·42) | -14·87 | 0·63(0·47-0·83) | 0·39(0·28-0·52) | -2(4·63--8·21) |
| Fiji | both | 2·06(1·14-2·67) | 2·01(1·19-2·57) | -2·23 | 0·46(0·25-0·6) | 0·26(0·15-0·33) | -2·63(5·57--10·2) |
| Finland | both | 76·04(70·41-81·96) | 34·16(30·39-39) | -55·07 | 1·07(1-1·15) | 0·3(0·27-0·34) | -5·33(0·46--10·78) |
| France | both | 1003·42(951·42-1052·08) | 739·66(656-830·11) | -26·29 | 1·2(1·14-1·26) | 0·48(0·43-0·54) | -3·58(1·29--8·22) |
| Gabon | both | 2·67(1·63-3·56) | 4·98(3·31-6·93) | 86·34 | 0·45(0·28-0·61) | 0·45(0·3-0·64) | 0·26(7·16--6·19) |
| Georgia | both | 11·84(9·11-14·24) | 13·54(11·94-15·5) | 14·36 | 0·19(0·15-0·23) | 0·26(0·23-0·3) | 1·47(12·89--8·8) |
| Germany | both | 2001·46(1774·61-2203·51) | 1114·18(987·52-1257·97) | -44·33 | 1·57(1·4-1·72) | 0·56(0·5-0·63) | -4·26(0·39--8·7) |
| Ghana | both | 42·39(31·67-53·41) | 72·7(55·84-101·42) | 71·51 | 0·52(0·41-0·64) | 0·4(0·31-0·55) | -1·04(5·92--7·53) |
| Greece | both | 122·36(113·81-132·04) | 139·03(121·79-157) | 13·63 | 0·8(0·74-0·86) | 0·54(0·47-0·6) | -1·98(3·42--7·11) |
| Greenland | both | 0·44(0·34-0·53) | 0·26(0·2-0·33) | -40·38 | 1·12(0·8-1·37) | 0·41(0·32-0·52) | -4·24(1·28--9·45) |
| Grenada | both | 0·82(0·69-0·93) | 1·06(0·92-1·19) | 29·61 | 1·15(0·98-1·32) | 0·75(0·65-0·85) | -1·73(3·43--6·63) |
| Guam | both | 0·34(0·28-0·43) | 0·54(0·45-0·69) | 55·64 | 0·38(0·31-0·48) | 0·29(0·24-0·38) | -1·27(6·69--8·63) |
| Guatemala | both | 6·48(5·48-8·06) | 25·39(21·76-28·98) | 291·66 | 0·17(0·14-0·21) | 0·22(0·19-0·25) | 0·63(11·01--8·78) |
| Guinea | both | 16·5(12·14-20·36) | 30·23(22·63-42·22) | 83·27 | 0·46(0·35-0·57) | 0·49(0·37-0·69) | 0·38(7·32--6·1) |
| Guinea-Bissau | both | 3·18(2·36-4·44) | 5·1(3·64-6·93) | 60·33 | 0·65(0·5-0·87) | 0·55(0·39-0·72) | -0·59(5·5--6·32) |
| Guyana | both | 3·45(3·1-3·81) | 4(3·42-4·6) | 16·09 | 0·77(0·69-0·84) | 0·62(0·53-0·71) | -0·25(5·45--5·65) |
| Haiti | both | 35·16(25·21-52·84) | 53·08(37·1-72·94) | 50·97 | 0·96(0·7-1·38) | 0·73(0·52-0·99) | -1·07(3·99--5·89) |
| Honduras | both | 28·4(22·25-36·2) | 60·92(44·21-82·24) | 114·54 | 1·15(0·87-1·48) | 0·96(0·68-1·28) | -0·79(3·63--5·01) |
| Hungary | both | 162·18(151·67-171·98) | 57·04(51·08-63·2) | -64·83 | 1·13(1·07-1·2) | 0·31(0·28-0·35) | -5·43(0·48--11) |
| Iceland | both | 2·86(2·58-3·16) | 2·61(2·24-2·98) | -8·86 | 0·97(0·88-1·07) | 0·49(0·42-0·56) | -1·53(3·56--6·38) |
| India | both | 2983·86(2500·53-3759·54) | 5371·36(4511·87-6188·22) | 80·01 | 0·58(0·49-0·74) | 0·5(0·42-0·57) | -0·66(5·71--6·64) |
| Indonesia | both | 315·79(249·53-536·35) | 590·76(449·32-845·55) | 87·07 | 0·27(0·22-0·47) | 0·27(0·21-0·38) | 0·08(9--8·12) |
| Iran | both | 188·55(99·43-236·17) | 195·81(154·25-242·4) | 3·85 | 0·6(0·33-0·75) | 0·27(0·21-0·34) | -2·79(4·54--9·61) |
| Iraq | both | 40·07(29·47-59·03) | 44·36(34·76-61·58) | 10·71 | 0·44(0·32-0·64) | 0·16(0·13-0·23) | -3·66(4·65--11·31) |
| Ireland | both | 42·25(39·01-45·77) | 24·94(22·02-28·39) | -40·97 | 1·03(0·95-1·11) | 0·35(0·31-0·39) | -3·77(1·99--9·2) |
| Israel | both | 51·27(46·02-56·76) | 40·19(35·59-44·98) | -21·61 | 1·08(0·98-1·19) | 0·34(0·3-0·38) | -4·57(0·87--9·72) |
| Italy | both | 1006·23(958·07-1053·97) | 733·54(658·59-809·63) | -27·1 | 1·16(1·11-1·21) | 0·46(0·41-0·5) | -3·59(1·65--8·57) |
| Jamaica | both | 5·01(4·32-5·67) | 13·44(10·54-17·08) | 168·39 | 0·28(0·24-0·31) | 0·46(0·36-0·59) | 2·37(10·52--5·17) |
| Japan | both | 869·56(840·31-897·97) | 473·89(441·51-522·38) | -45·5 | 0·54(0·52-0·56) | 0·14(0·13-0·15) | -5·83(3·16--14·04) |
| Jordan | both | 2·5(1·74-3·39) | 4·69(3·61-6·18) | 87·42 | 0·14(0·09-0·18) | 0·07(0·05-0·09) | -2·91(12·31--16·07) |
| Kazakhstan | both | 79·42(63·57-103·21) | 53·62(41·79-70·64) | -32·49 | 0·55(0·44-0·71) | 0·3(0·23-0·39) | -2·19(4·57--8·51) |
| Kenya | both | 15·98(12·11-23·38) | 36·26(29·22-46·55) | 126·89 | 0·16(0·12-0·25) | 0·15(0·12-0·2) | -0·26(11·6--10·85) |
| Kiribati | both | 0·29(0·21-0·36) | 0·43(0·31-0·56) | 48·83 | 0·64(0·46-0·8) | 0·54(0·4-0·71) | -0·49(5·67--6·29) |
| Kuwait | both | 1·9(1·63-2·22) | 3·63(3-4·3) | 90·61 | 0·19(0·16-0·23) | 0·12(0·1-0·14) | -1·43(10·95--12·43) |
| Kyrgyzstan | both | 10·73(9·2-12·45) | 7·79(6·73-9·02) | -27·43 | 0·32(0·28-0·38) | 0·16(0·14-0·18) | -2·35(6·89--10·8) |
| Laos | both | 10·13(6·56-19·92) | 14(10·02-20·72) | 38·25 | 0·43(0·29-0·84) | 0·3(0·22-0·44) | -1·26(6·44--8·41) |
| Latvia | both | 27·3(22·92-32·64) | 16·49(14-19·5) | -39·61 | 0·75(0·63-0·89) | 0·42(0·36-0·5) | -2·49(3·42--8·07) |
| Lebanon | both | 7·59(4·91-10·76) | 12·33(8·62-16·28) | 62·42 | 0·31(0·2-0·43) | 0·19(0·13-0·25) | -2(7·88--10·97) |
| Lesotho | both | 1·28(0·84-1·72) | 2·02(1·37-2·78) | 58·11 | 0·12(0·08-0·16) | 0·16(0·11-0·22) | 1·63(14·27--9·62) |
| Liberia | both | 5·3(4·03-6·69) | 9·95(7·5-12·61) | 87·84 | 0·45(0·35-0·58) | 0·42(0·32-0·54) | -0·41(6·73--7·07) |
| Libya | both | 5·74(3·9-7·79) | 12·55(9·34-16·22) | 118·67 | 0·27(0·18-0·37) | 0·24(0·18-0·31) | -0·41(9·08--9·07) |
| Lithuania | both | 50·4(44·83-55·45) | 21·64(18·98-25·84) | -57·06 | 1·11(0·99-1·22) | 0·41(0·36-0·49) | -3·58(1·5--8·41) |
| Luxembourg | both | 5·05(4·38-5·81) | 3·61(3·07-4·26) | -28·51 | 0·92(0·81-1·06) | 0·36(0·31-0·43) | -3·96(1·97--9·54) |
| Macedonia | both | 8·65(6·93-10·91) | 9·22(7·27-12·08) | 6·53 | 0·45(0·36-0·58) | 0·29(0·23-0·37) | -1·99(5·77--9·17) |
| Madagascar | both | 29·35(23·39-40·14) | 51·82(39·16-66·45) | 76·56 | 0·47(0·38-0·65) | 0·42(0·32-0·54) | -0·33(6·82--7·01) |
| Malawi | both | 16·24(8·6-21·99) | 24·47(17·73-30·25) | 50·65 | 0·36(0·21-0·48) | 0·3(0·21-0·37) | -1·15(6·82--8·53) |
| Malaysia | both | 63·27(51·2-83) | 108·78(79·19-170·9) | 71·93 | 0·62(0·49-0·89) | 0·43(0·32-0·7) | -1·25(5·22--7·32) |
| Maldives | both | 0·93(0·5-1·79) | 1·15(0·92-1·44) | 23·52 | 0·8(0·46-1·5) | 0·36(0·28-0·45) | -3·32(2·91--9·17) |
| Mali | both | 19·71(15·38-24·91) | 31·86(23·14-43·27) | 61·68 | 0·43(0·34-0·54) | 0·35(0·25-0·46) | -0·86(6·82--7·98) |
| Malta | both | 4·31(3·88-4·75) | 3·83(3·34-4·33) | -11·28 | 1·03(0·93-1·12) | 0·44(0·39-0·49) | -3·68(1·83--8·88) |
| Marshall Islands | both | 0·12(0·09-0·16) | 0·16(0·11-0·23) | 37·06 | 0·55(0·42-0·72) | 0·41(0·28-0·56) | -1·22(5·45--7·46) |
| Mauritania | both | 5·53(4·26-6·99) | 9·23(6·64-11·89) | 67 | 0·48(0·37-0·61) | 0·43(0·31-0·55) | -0·34(6·75--6·95) |
| Mauritius | both | 1·59(1·42-1·77) | 2·95(2·51-3·41) | 85·33 | 0·2(0·18-0·23) | 0·18(0·15-0·2) | -0·45(11·29--10·95) |
| Mexico | both | 234·46(226·38-243·6) | 350·16(331·31-370·09) | 49·35 | 0·46(0·44-0·48) | 0·3(0·29-0·32) | -1·95(5·41--8·8) |
| Moldova | both | 9·24(7·49-11·08) | 5·13(4·47-5·97) | -44·52 | 0·2(0·16-0·24) | 0·09(0·08-0·11) | -2·74(9·81--13·85) |
| Mongolia | both | 4·23(3·39-5·34) | 5·96(4·71-8·08) | 41·09 | 0·35(0·28-0·44) | 0·23(0·19-0·3) | -1·94(6·42--9·65) |
| Montenegro | both | 4·34(3·43-5·58) | 4·71(3·74-6·16) | 8·56 | 0·68(0·54-0·87) | 0·49(0·39-0·64) | -1·4(4·53--7·01) |
| Morocco | both | 33·01(14·72-44·3) | 44·32(23·49-59·31) | 34·28 | 0·21(0·09-0·28) | 0·14(0·07-0·18) | -1·59(9·87--11·86) |
| Mozambique | both | 43(31·18-70·96) | 65·8(48·73-114·31) | 53·03 | 0·66(0·5-1·02) | 0·56(0·43-0·88) | -0·6(5·56--6·41) |
| Myanmar | both | 150·92(96·07-327·48) | 163·13(120·21-248·73) | 8·09 | 0·57(0·37-1·27) | 0·35(0·26-0·54) | -1·7(5·03--8) |
| Namibia | both | 0·84(0·52-1·17) | 1·08(0·68-1·53) | 27·59 | 0·11(0·07-0·15) | 0·07(0·05-0·1) | -1·61(13·8--14·94) |
| Nepal | both | 75·98(56·4-105·89) | 110·9(82·57-148·23) | 45·96 | 0·73(0·54-1·02) | 0·52(0·39-0·69) | -1·28(4·8--7·02) |
| Netherlands | both | 196·09(185·47-207·68) | 108·22(97·2-122·43) | -44·81 | 0·97(0·92-1·03) | 0·32(0·29-0·36) | -3·76(1·93--9·13) |
| New Zealand | both | 42·56(38·74-45·95) | 27·35(24·26-30·82) | -35·75 | 1·07(0·98-1·16) | 0·37(0·33-0·42) | -4·63(1·15--10·08) |
| Nicaragua | both | 7·92(6·63-9·3) | 15·24(11·94-18·35) | 92·38 | 0·39(0·33-0·47) | 0·31(0·24-0·37) | -0·77(7--7·98) |
| Niger | both | 16·74(12·33-21·53) | 37·8(26·95-49·46) | 125·78 | 0·46(0·35-0·6) | 0·44(0·32-0·57) | -0·39(6·66--6·97) |
| Nigeria | both | 149·96(98·11-234·03) | 275·97(169·08-427·18) | 84·03 | 0·3(0·2-0·47) | 0·29(0·19-0·44) | -0·16(8·61--8·23) |
| North Korea | both | 33·51(25·23-43·16) | 43·5(32·67-59·07) | 29·84 | 0·18(0·13-0·23) | 0·14(0·11-0·19) | -0·9(10·93--11·47) |
| Northern Mariana Islands | both | 0·13(0·09-0·17) | 0·12(0·09-0·16) | -7·78 | 0·5(0·35-0·63) | 0·22(0·18-0·31) | -3·8(4·32--11·28) |
| Norway | both | 45·91(44·38-47·54) | 24·7(23·52-26·01) | -46·21 | 0·68(0·66-0·71) | 0·26(0·25-0·28) | -3·68(3·23--10·12) |
| Oman | both | 1·76(1·22-2·34) | 3·03(2·08-3·99) | 71·97 | 0·2(0·13-0·26) | 0·12(0·08-0·16) | -1·75(10·28--12·47) |
| Pakistan | both | 385·13(282·7-521·1) | 711·17(498·32-977·31) | 84·66 | 0·6(0·45-0·83) | 0·57(0·41-0·78) | -0·3(5·61--5·88) |
| Palestine | both | 2·17(1·58-3·28) | 4·6(3·41-6·49) | 112·09 | 0·2(0·15-0·31) | 0·16(0·11-0·21) | -0·97(10·34--11·12) |
| Panama | both | 9·82(8·79-10·9) | 12·75(11·33-14·35) | 29·87 | 0·61(0·55-0·67) | 0·32(0·28-0·36) | -2·37(4·84--9·09) |
| Papua New Guinea | both | 18·62(13·69-25·5) | 31·51(22·42-45·25) | 69·22 | 0·74(0·56-0·98) | 0·52(0·39-0·72) | -1·27(4·64--6·85) |
| Paraguay | both | 7·05(5·7-8·88) | 17·8(13·74-22·61) | 152·32 | 0·3(0·24-0·37) | 0·33(0·25-0·42) | 1(9·31--6·69) |
| Peru | both | 35·64(28·06-43·96) | 62·3(45·15-75·84) | 74·78 | 0·25(0·2-0·31) | 0·2(0·14-0·24) | -1·03(8·79--9·97) |
| Philippines | both | 81·7(71·93-93·36) | 235·66(201·05-273·29) | 188·44 | 0·21(0·19-0·24) | 0·3(0·25-0·34) | 1·51(10·88--7·07) |
| Poland | both | 393·68(354·92-432·59) | 247·74(221·76-275·64) | -37·07 | 0·88(0·8-0·97) | 0·36(0·32-0·4) | -3·59(2·23--9·08) |
| Portugal | both | 113·68(106·27-121·69) | 88·96(78·8-99·86) | -21·74 | 0·85(0·8-0·91) | 0·36(0·32-0·4) | -4·11(1·82--9·69) |
| Puerto Rico | both | 25·26(22·69-27·97) | 12·99(11·36-15·24) | -48·57 | 0·69(0·62-0·76) | 0·2(0·17-0·23) | -5·73(1·92--12·8) |
| Qatar | both | 0·84(0·49-1·17) | 2·75(1·69-6·32) | 226·93 | 0·59(0·32-0·84) | 0·28(0·2-0·37) | -3·58(3·55--10·22) |
| Romania | both | 87·11(76·79-98·95) | 70·69(62·9-78·49) | -18·85 | 0·32(0·28-0·35) | 0·2(0·18-0·23) | -1·66(7·22--9·81) |
| Russian Federation | both | 841·82(758·86-951·02) | 819·59(771·16-863·57) | -2·64 | 0·46(0·42-0·52) | 0·37(0·35-0·39) | -0·94(5·84--7·29) |
| Rwanda | both | 19·83(14·58-32·74) | 24·84(18·45-31·92) | 25·23 | 0·59(0·45-0·97) | 0·4(0·3-0·52) | -1·79(4·75--7·92) |
| Saint Lucia | both | 0·93(0·83-1·04) | 1·09(0·96-1·23) | 16·85 | 0·98(0·87-1·09) | 0·52(0·46-0·59) | -2·77(2·86--8·1) |
| Saint Vincent and the Grenadines | both | 1·26(1·12-1·39) | 0·74(0·66-0·83) | -41·38 | 1·62(1·45-1·79) | 0·56(0·5-0·63) | -4·67(0·51--9·58) |
| Samoa | both | 0·35(0·26-0·49) | 0·36(0·26-0·51) | 4·24 | 0·36(0·27-0·52) | 0·26(0·19-0·36) | -1·5(7·11--9·41) |
| Sao Tome and Principe | both | 0·24(0·18-0·32) | 0·46(0·32-0·64) | 87·96 | 0·34(0·25-0·44) | 0·36(0·25-0·51) | 0·15(8·05--7·17) |
| Saudi Arabia | both | 30·11(19·78-40·85) | 62·67(46·99-80·68) | 108·15 | 0·47(0·3-0·65) | 0·4(0·31-0·5) | -0·49(6·61--7·12) |
| Senegal | both | 18·89(15·09-23·41) | 40·29(30·71-51·88) | 113·28 | 0·51(0·41-0·63) | 0·5(0·38-0·64) | 0·07(6·79--6·23) |
| Serbia | both | 27·01(21·15-32·95) | 37·79(27·63-44·93) | 39·93 | 0·25(0·2-0·31) | 0·25(0·18-0·3) | 0·74(10·1--7·83) |
| Seychelles | both | 0·55(0·45-0·79) | 0·86(0·62-1·03) | 56·41 | 0·94(0·77-1·36) | 0·82(0·6-0·98) | -0·61(4·2--5·2) |
| Sierra Leone | both | 10·99(8·62-14·36) | 20·22(15·46-26·23) | 83·92 | 0·49(0·39-0·64) | 0·49(0·38-0·63) | 0·14(6·84--6·14) |
| Singapore | both | 13·79(12·52-14·95) | 8·41(7·32-9·59) | -39·02 | 0·5(0·46-0·55) | 0·12(0·11-0·14) | -5·31(3·65--13·5) |
| Slovakia | both | 37·24(29·34-47·09) | 16·01(11·99-19·34) | -57 | 0·62(0·48-0·78) | 0·18(0·14-0·22) | -4·38(3·07--11·28) |
| Slovenia | both | 24·32(22·32-26·46) | 35·99(30·91-41·89) | 47·99 | 0·99(0·91-1·07) | 0·84(0·72-0·96) | -0·07(4·77--4·69) |
| Solomon Islands | both | 1·12(0·81-1·56) | 1·7(1·24-2·32) | 51·75 | 0·62(0·46-0·84) | 0·43(0·32-0·58) | -1·32(5·17--7·41) |
| Somalia | both | 16·02(8·5-25·54) | 43·24(29·41-60·95) | 169·96 | 0·53(0·33-0·82) | 0·61(0·42-0·84) | 0·3(6·47--5·5) |
| South Africa | both | 18·05(14-21·85) | 20·37(17·05-25·57) | 12·9 | 0·07(0·06-0·09) | 0·04(0·04-0·05) | -1·86(17·56--18·07) |
| South Korea | both | 147·16(130·83-170·73) | 114·81(96·83-133·09) | -21·99 | 0·36(0·32-0·42) | 0·14(0·12-0·16) | -2·29(7·17--10·92) |
| South Sudan | both | 11·17(6·39-17·27) | 19·2(12·91-27·24) | 71·93 | 0·41(0·27-0·6) | 0·45(0·31-0·62) | 0·15(7·41--6·61) |
| Spain | both | 477·5(449·69-502·71) | 318·61(283·4-353·62) | -33·28 | 0·89(0·84-0·94) | 0·3(0·27-0·33) | -4·47(1·5--10·1) |
| Sri Lanka | both | 43·3(35·78-54·6) | 40·51(30·51-50·9) | -6·45 | 0·33(0·28-0·42) | 0·17(0·13-0·21) | -2·45(7·05--11·1) |
| Sudan | both | 49·53(25·15-73·22) | 64·77(34·42-87·63) | 30·77 | 0·46(0·22-0·67) | 0·31(0·16-0·43) | -1·49(6·13--8·56) |
| Suriname | both | 1·14(0·99-1·28) | 1·68(1·45-1·92) | 46·44 | 0·41(0·35-0·46) | 0·29(0·25-0·33) | -1·44(6·57--8·85) |
| Swaziland | both | 0·43(0·28-0·56) | 0·73(0·45-0·99) | 70·9 | 0·12(0·08-0·16) | 0·11(0·07-0·16) | 0·39(13·6--11·28) |
| Sweden | both | 97·5(91·46-103·41) | 78·21(71·72-85·34) | -19·79 | 0·67(0·63-0·71) | 0·38(0·34-0·41) | -2·17(4·53--8·44) |
| Switzerland | both | 66·64(59·02-76·54) | 54·5(48·37-61·51) | -18·21 | 0·62(0·55-0·72) | 0·3(0·27-0·34) | -3·19(3·33--9·3) |
| Syria | both | 52·77(35·17-80·93) | 66·55(47·81-99·69) | 26·12 | 0·89(0·58-1·37) | 0·51(0·36-0·75) | -2·66(2·89--7·9) |
| Tajikistan | both | 12·87(9·49-16·9) | 12·36(9·41-17·49) | -3·93 | 0·39(0·28-0·52) | 0·2(0·15-0·27) | -2·98(6·21--11·38) |
| Tanzania | both | 54·37(37·63-73·72) | 104·28(74·75-131·68) | 91·78 | 0·44(0·32-0·6) | 0·4(0·29-0·5) | -0·63(6·66--7·42) |
| Thailand | both | 225·67(160·44-272·14) | 318·88(205·95-390·51) | 41·31 | 0·56(0·39-0·67) | 0·34(0·22-0·42) | -2·19(4·78--8·7) |
| The Bahamas | both | 1·54(1·38-1·72) | 2·42(2·11-2·77) | 56·69 | 0·86(0·77-0·95) | 0·63(0·55-0·72) | -1·11(4·43--6·36) |
| The Gambia | both | 2(1·5-2·73) | 5·21(3·87-7·09) | 160·48 | 0·44(0·34-0·6) | 0·47(0·35-0·63) | 0·41(7·38--6·11) |
| Timor-Leste | both | 1·21(0·8-2·08) | 2·42(1·62-3·44) | 99·71 | 0·32(0·21-0·56) | 0·3(0·2-0·42) | -0·29(8·35--8·23) |
| Togo | both | 7·98(6·24-9·95) | 18·19(13·28-24) | 128·01 | 0·49(0·38-0·61) | 0·44(0·33-0·56) | -0·48(6·32--6·84) |
| Tonga | both | 0·18(0·14-0·22) | 0·17(0·13-0·21) | -5·56 | 0·29(0·23-0·37) | 0·2(0·16-0·25) | -1·51(8·14--10·3) |
| Trinidad and Tobago | both | 7·02(6·26-7·88) | 6·61(5·19-8·34) | -5·9 | 0·76(0·67-0·85) | 0·38(0·3-0·47) | -3·15(3·2--9·11) |
| Tunisia | both | 12·04(8·09-17·93) | 19·81(14·27-26·8) | 64·59 | 0·24(0·15-0·35) | 0·17(0·12-0·22) | -1·52(9--11·03) |
| Turkey | both | 297·77(227·22-370·49) | 251·83(207·33-330·43) | -15·43 | 0·79(0·6-0·99) | 0·29(0·24-0·39) | -3·88(2·7--10·03) |
| Turkmenistan | both | 6·91(5·06-8·92) | 8·32(6·2-10·68) | 20·39 | 0·3(0·22-0·38) | 0·2(0·15-0·25) | -1·61(7·8--10·19) |
| Uganda | both | 27·71(21·69-34·72) | 55·81(42·55-69·29) | 101·4 | 0·37(0·29-0·46) | 0·37(0·28-0·45) | -0·16(7·41--7·2) |
| Ukraine | both | 462·44(392·79-551·75) | 301·87(270·48-341·57) | -34·72 | 0·66(0·57-0·79) | 0·43(0·38-0·49) | -2·69(3·69--8·68) |
| United Arab Emirates | both | 2·76(1·13-4·15) | 16·85(8·44-25·63) | 510·45 | 0·47(0·15-0·75) | 0·36(0·13-0·64) | -1·13(6·18--7·94) |
| United Kingdom | both | 1030·74(983·71-1085·15) | 449·47(428·71-484·73) | -56·39 | 1·14(1·08-1·2) | 0·34(0·33-0·37) | -5·61(0·26--11·14) |
| United States | both | 2576·48(2508·04-2651·17) | 1389·62(1331·6-1462·78) | -46·07 | 0·81(0·79-0·83) | 0·26(0·25-0·28) | -5·16(1·71--11·57) |
| Uruguay | both | 37·01(33·99-40·29) | 21·74(18·9-25·3) | -41·25 | 0·99(0·91-1·08) | 0·41(0·36-0·48) | -3·57(2·04--8·87) |
| Uzbekistan | both | 43·14(32·29-52·68) | 59·29(47·52-72·19) | 37·43 | 0·31(0·23-0·39) | 0·24(0·19-0·29) | -0·83(7·94--8·88) |
| Vanuatu | both | 0·63(0·34-1·01) | 1·09(0·54-1·83) | 72·72 | 0·74(0·41-1·16) | 0·57(0·28-0·93) | -1·05(4·68--6·47) |
| Venezuela | both | 94·08(85·41-104·25) | 116·91(98·65-136·67) | 24·26 | 0·83(0·76-0·92) | 0·42(0·35-0·49) | -2·88(3·17--8·57) |
| Vietnam | both | 72·33(57·66-95·82) | 130·2(92·22-162·33) | 80·01 | 0·17(0·14-0·23) | 0·14(0·1-0·18) | -0·45(11·64--11·23) |
| Virgin Islands, U.S. | both | 0·48(0·42-0·57) | 0·52(0·43-0·61) | 7·01 | 0·56(0·48-0·66) | 0·31(0·26-0·36) | -2·54(5·09--9·62) |
| Yemen | both | 33·81(16-53·95) | 66·27(34·73-94·83) | 96 | 0·56(0·27-0·86) | 0·44(0·22-0·62) | -1·06(5·53--7·24) |
| Zambia | both | 20·16(14·05-28·47) | 34·47(25·16-44·16) | 70·94 | 0·59(0·44-0·81) | 0·47(0·34-0·6) | -1·28(5·07--7·26) |
| Zimbabwe | both | 5·25(3·24-8·62) | 11·84(7·07-15·56) | 125·69 | 0·11(0·07-0·18) | 0·15(0·1-0·21) | 1·99(14·58--9·22) |

ASDR, age-standardized death rate; EAPC, estimated annual percentage change.

**Supplementary table S13. DALY and age-standardized DALY rate by countries in 1990 and 2017 and its change trends from 1990 to 2017.**

| Nation | Sex | DALY  No.×10^3^  (95% UI) | | Change (%) | DALYs  per 100,000  No.(95% UI) | | EAPC |
| --- | --- | --- | --- | --- | --- | --- | --- |
|  |  | 1990 | 2017 |  | 1990 | 2017 |  |
| Afghanistan | both | 2077·98(747·51-4194·88) | 4710·87(2309·28-8567·82) | 126·7 | 28·28(10·08-57·91) | 24·16(11·51-41·45) | -0·69(0·17--1·54) |
| Albania | both | 333·3(240·26-514·18) | 279·5(177·19-481·89) | -16·14 | 11·81(8·38-18·79) | 8·06(5·26-13·16) | -1·53(-0·09--2·95) |
| Algeria | both | 3051·59(2460·17-3673·05) | 4735·74(3585·11-5623·23) | 55·19 | 17·83(14·19-21·38) | 12·33(9·48-14·57) | -1·18(-0·02--2·33) |
| American Samoa | both | 4·13(3·17-4·98) | 3·92(3·24-4·81) | -5·12 | 12·57(9·53-15·14) | 7·7(6·39-9·49) | -2·22(-0·78--3·63) |
| Andorra | both | 11·24(8·82-13·89) | 10·41(8·14-13·49) | -7·4 | 18·07(14·19-22·27) | 8·49(6·69-10·99) | -3·16(-1·86--4·44) |
| Angola | both | 1072·42(611·25-1507·25) | 2444·4(1607·31-3327·52) | 127·93 | 16·87(10·44-23·01) | 15·04(9·97-20·37) | -0·32(0·8--1·43) |
| Antigua and Barbuda | both | 9·96(8·7-11·28) | 8·69(7·54-9·88) | -12·69 | 18·38(16·03-20·83) | 8·54(7·41-9·68) | -3·24(-1·96--4·5) |
| Argentina | both | 5951·43(5294·57-6624·8) | 3206·31(2768-3653·31) | -46·13 | 18·23(16·25-20·28) | 6·39(5·51-7·31) | -4·36(-3·06--5·64) |
| Armenia | both | 628·02(542·79-733·78) | 432·5(375·61-493·29) | -31·13 | 19·2(16·6-22·05) | 10·77(9·42-12·17) | -2·21(-1·04--3·37) |
| Australia | both | 5759·65(5470·57-6075·35) | 3467·61(2994·51-4012·6) | -39·79 | 29·63(28·15-31·28) | 10·04(8·61-11·55) | -4·72(-3·67--5·77) |
| Austria | both | 2657·57(2481·26-2828·16) | 1636·95(1466·8-1842·72) | -38·4 | 25·23(23·48-26·96) | 10·76(9·67-12·2) | -3·29(-2·2--4·37) |
| Azerbaijan | both | 494·32(377·36-676·36) | 722·62(552·75-908·63) | 46·18 | 7·8(6·06-10·66) | 6·66(5·12-8·28) | -0·98(0·64--2·57) |
| Bahrain | both | 40·11(29·51-57·27) | 61·29(47·99-85·39) | 52·81 | 13·79(9·23-17·82) | 4·93(3·88-6·35) | -5·11(-3·64--6·55) |
| Bangladesh | both | 15092·7(11455·72-19886·31) | 14444·31(11037·49-19389·28) | -4·3 | 22·02(17·09-28·83) | 10·2(7·93-13·38) | -2·75(-1·58--3·9) |
| Barbados | both | 51·57(45·01-58·54) | 52·68(45·26-60·96) | 2·16 | 18·86(16·38-21·39) | 13·06(11·19-15·08) | -1·52(-0·38--2·65) |
| Belarus | both | 2179·74(1819·14-2541·19) | 1494·82(1284·58-1747·79) | -31·42 | 17·75(14·96-20·48) | 10·63(9·14-12·41) | -1·52(-0·38--2·65) |
| Belgium | both | 3174·16(2933·06-3432·02) | 1764·63(1587·59-1957·67) | -44·41 | 22·11(20·46-23·92) | 8·74(7·84-9·79) | -3·68(-2·54--4·8) |
| Belize | both | 7·52(5·76-9·1) | 19·5(16·74-22·51) | 159·49 | 6·06(4·72-7·27) | 5·68(4·92-6·5) | -0·49(1·32--2·28) |
| Benin | both | 416·29(326·61-512·72) | 936·21(692·71-1217·49) | 124·89 | 15·22(11·82-18·9) | 13·29(9·87-17·12) | -0·54(0·66--1·73) |
| Bermuda | both | 17·14(14·25-20·94) | 12·2(10·43-14·31) | -28·86 | 25·69(21·32-31·45) | 12·08(10·35-14·3) | -2·78(-1·65--3·89) |
| Bhutan | both | 71·91(56·07-93·28) | 77·28(51·06-103·67) | 7·47 | 19·19(15·27-24·74) | 9·65(6·64-12·64) | -2·68(-1·46--3·88) |
| Bolivia | both | 671·08(480·69-914·05) | 1025·15(694·63-1344·3) | 52·76 | 14·96(10·86-20·1) | 10·33(7·04-13·53) | -1·43(-0·16--2·68) |
| Bosnia and Herzegovina | both | 722·62(539·4-1035·96) | 532·85(414·4-685·86) | -26·26 | 15·28(11·34-21·98) | 10·21(7·84-13·2) | -1·94(-0·66--3·2) |
| Botswana | both | 24·07(14·6-34·39) | 39·18(21·93-51·62) | 62·78 | 3·07(1·85-4·28) | 2·22(1·25-2·86) | -0·61(2·23--3·37) |
| Brazil | both | 20273·6(19404·64-21236·34) | 15263·27(14526·31-16082·11) | -24·71 | 17·2(16·52-17·96) | 6·59(6·28-6·93) | -3·77(-2·46--5·07) |
| Brunei | both | 86·78(69·32-106·91) | 147·13(118·25-177·55) | 69·55 | 43·44(35·22-56·43) | 33·88(27·87-40·17) | -0·68(0·1--1·45) |
| Bulgaria | both | 1688·91(1490·02-1950·25) | 913·28(803·83-1030·78) | -45·93 | 14·54(12·75-16·73) | 7·28(6·39-8·26) | -2·4(-1·07--3·7) |
| Burkina Faso | both | 1032·7(776·62-1335·34) | 1964·61(1449·49-2536·88) | 90·24 | 18·36(13·91-23·79) | 15·1(11·3-19·27) | -0·8(0·34--1·94) |
| Burundi | both | 563·84(389·89-860·02) | 746·43(558·11-967·92) | 32·39 | 17·36(12·22-26·52) | 12·07(9·18-15·5) | -1·58(-0·38--2·77) |
| Cambodia | both | 834·51(560·43-1645·96) | 1261·78(940·37-1932·67) | 51·2 | 13·39(9·23-26·68) | 9·33(7·05-14·33) | -1·31(0·04--2·64) |
| Cameroon | both | 1042·46(804·04-1304·39) | 2508·36(1739·81-3321·95) | 140·62 | 16·31(12·65-20·37) | 14·09(9·87-18·63) | -0·54(0·59--1·67) |
| Canada | both | 7023·15(6574·59-7448·02) | 3318·49(2936·23-3746·25) | -52·75 | 22·08(20·6-23·44) | 6·04(5·36-6·77) | -5·85(-4·53--7·14) |
| Cape Verde | both | 23·96(15·94-37·43) | 61·96(44·26-84·94) | 158·56 | 10·12(6·82-15·55) | 11·08(7·97-15·17) | 0·24(1·6--1·11) |
| Central African Republic | both | 323·78(191·12-452·44) | 672·68(427·51-907·58) | 107·76 | 18·81(12·08-25·24) | 21·36(14·11-27·73) | 0·54(1·55--0·46) |
| Chad | both | 494·74(371·77-641·87) | 1150·14(851·17-1453·43) | 132·47 | 13·86(10·39-18·06) | 14·4(10·65-18·27) | 0·26(1·48--0·94) |
| Chile | both | 2280·16(2076·77-2478·9) | 1672·6(1445·74-1943·64) | -26·65 | 19·37(17·67-21·01) | 7·66(6·64-8·91) | -3·41(-2·16--4·64) |
| China | both | 58729·69(43175·73-74944·34) | 32957·43(27972·78-39594·67) | -43·88 | 4·95(3·73-6·23) | 1·81(1·56-2·17) | -4·44(-1·92--6·89) |
| Colombia | both | 5095·45(4732·87-5465) | 5819·49(5024·09-6675·37) | 14·21 | 21·31(19·93-22·78) | 10·92(9·43-12·52) | -2·8(-1·68--3·91) |
| Comoros | both | 47·48(36·38-65·91) | 71·1(51·72-91·14) | 49·76 | 16·68(12·83-23·04) | 12·83(9·48-16·59) | -1·02(0·19--2·21) |
| Congo | both | 274·44(155·98-367·15) | 590(366·42-831·47) | 114·98 | 18·22(10·77-23·87) | 16·72(10·72-22·48) | -0·4(0·69--1·48) |
| Costa Rica | both | 593·89(538·1-653·77) | 940·77(816·26-1063·57) | 58·41 | 28·59(25·96-31·38) | 18·97(16·46-21·42) | -1·45(-0·5--2·39) |
| Cote d'Ivoire | both | 979·11(729·42-1224·9) | 2161·5(1637·83-2850·42) | 120·76 | 13·78(10·22-17·18) | 13·01(9·96-16·99) | -0·22(1--1·43) |
| Croatia | both | 1511·8(1324·99-1717·65) | 554·52(493·23-628·27) | -63·32 | 24·07(21·1-27·26) | 7·52(6·73-8·41) | -4·31(-3·11--5·51) |
| Cuba | both | 3510·35(3135·37-3917·89) | 2754·87(2378·85-3179·94) | -21·52 | 31·93(28·55-35·5) | 16·73(14·41-19·42) | -2·61(-1·66--3·55) |
| Cyprus | both | 69·97(57·66-87·46) | 68·43(56·47-86·26) | -2·21 | 8·37(6·83-10·4) | 3·99(3·31-5·07) | -2·79(-1·02--4·54) |
| Czech Republic | both | 2254·9(2074·47-2425·96) | 1237·03(1107·87-1374·64) | -45·14 | 18·06(16·61-19·47) | 7·7(6·9-8·55) | -3·51(-2·3--4·7) |
| Democratic Republic of the Congo | both | 3054·84(1941·48-4070·25) | 7064·46(5036·97-9207·59) | 131·25 | 13·26(8·75-17·51) | 14·57(10·37-18·9) | 0·52(1·76--0·7) |
| Denmark | both | 2609·91(2439·8-2782·64) | 1255·66(1121·18-1400·18) | -51·89 | 36·03(33·66-38·52) | 13·03(11·71-14·52) | -3·95(-3·05--4·83) |
| Djibouti | both | 38·38(25·32-53·65) | 95·09(62·54-139·88) | 147·77 | 14·05(9·71-19·34) | 11·72(7·75-16·76) | -0·81(0·44--2·05) |
| Dominica | both | 12·1(10·14-14·21) | 13·84(11·84-16·22) | 14·34 | 18·01(15·12-21·11) | 16·92(14·51-19·81) | -0·35(0·75--1·45) |
| Dominican Republic | both | 244·17(195·17-319·53) | 534·79(414·82-703·58) | 119·03 | 4·78(3·82-6·3) | 5·31(4·11-6·95) | 0·44(2·6--1·67) |
| Ecuador | both | 580·84(498·14-675·68) | 1535·65(1326·23-1747·18) | 164·38 | 7·84(6·81-9·01) | 9·55(8·26-10·86) | 1·48(3·08--0·09) |
| Egypt | both | 2209·83(1487·37-3338·05) | 3371·28(2402·53-5416·98) | 52·56 | 5·37(3·59-8·36) | 4·12(2·92-6·8) | -0·99(1·12--3·05) |
| El Salvador | both | 197·08(161·3-264·79) | 455·09(316·66-590·21) | 130·92 | 5·61(4·6-7·44) | 7·83(5·42-10·03) | 1·91(3·71-0·14) |
| Equatorial Guinea | both | 48·21(28·64-71·11) | 69·56(33·9-114·89) | 44·31 | 18·02(11·07-25·55) | 9·39(4·67-15·71) | -2·85(-1·57--4·11) |
| Eritrea | both | 345·83(212·67-490·72) | 725·95(520·28-967·29) | 109·92 | 22·35(15·07-31·42) | 19·87(14·66-25·79) | -0·62(0·33--1·57) |
| Estonia | both | 402·12(344·13-467·02) | 212·59(179·15-250·23) | -47·13 | 21·29(18·19-24·85) | 9·93(8·19-11·84) | -3·17(-2·08--4·26) |
| Ethiopia | both | 27227·95(17927·04-40351·57) | 33234·36(23437·86-42680·41) | 22·06 | 89·34(62·95-126·93) | 54·13(38·02-69·9) | -2·08(-1·55--2·6) |
| Federated States of Micronesia | both | 12·9(9·35-17·75) | 10·92(7·16-15·23) | -15·33 | 19·95(14·55-26·89) | 12·24(8·22-16·84) | -2·04(-0·89--3·19) |
| Fiji | both | 75·43(42·12-97·79) | 65·04(38·56-83·11) | -13·77 | 14·03(7·75-18·25) | 7·5(4·46-9·52) | -2·81(-1·36--4·24) |
| Finland | both | 1760·98(1639·73-1892·58) | 663·88(590·44-753·24) | -62·3 | 26·74(24·89-28·72) | 7·47(6·6-8·41) | -5·22(-4·09--6·33) |
| France | both | 21829·95(20754·07-22960·38) | 11017·52(9884·94-12292·43) | -49·53 | 29·21(27·68-30·81) | 9·36(8·41-10·47) | -4·44(-3·42--5·45) |
| Gabon | both | 88·08(52·15-116·29) | 164·9(106·32-223·98) | 87·21 | 12·88(7·66-17·16) | 12·47(8·18-17·07) | 0·11(1·37--1·14) |
| Georgia | both | 416·87(319·89-508·47) | 415·82(363·51-477·1) | -0·25 | 6·9(5·3-8·44) | 9·02(7·78-10·43) | 1·18(3·01--0·62) |
| Germany | both | 44015·36(39383·19-48319·66) | 18614·87(16481·23-21062·62) | -57·71 | 38·63(34·56-42·47) | 11·68(10·28-13·23) | -4·89(-3·94--5·83) |
| Ghana | both | 1792·96(1262·91-2334·54) | 2786·11(2076·37-3990·17) | 55·39 | 17·52(12·99-22·27) | 12·17(9·29-17·2) | -1·6(-0·41--2·78) |
| Greece | both | 2611·13(2416·42-2827·77) | 2237·35(1962·91-2524·9) | -14·31 | 17·95(16·61-19·41) | 10·91(9·56-12·24) | -2·33(-1·18--3·48) |
| Greenland | both | 16·11(12·55-20·66) | 6·91(5·48-8·76) | -57·11 | 31·75(24·19-39·03) | 10·01(8·06-12·72) | -4·7(-3·65--5·73) |
| Grenada | both | 25·28(21·22-28·96) | 29·45(25·42-33·32) | 16·47 | 35·94(30·2-41·08) | 23·24(20·14-26·48) | -1·74(-0·83--2·64) |
| Guam | both | 11·82(9·51-14·68) | 16·97(14·26-22·05) | 43·57 | 10·96(8·88-13·63) | 9·24(7·76-12·07) | -0·88(0·52--2·26) |
| Guatemala | both | 204(169·68-261·86) | 760·66(648·86-866·72) | 272·87 | 4·39(3·69-5·53) | 5·97(5·11-6·85) | 0·75(2·7--1·15) |
| Guinea | both | 568·63(411·88-707·72) | 1076·42(804·34-1502·67) | 89·3 | 14·18(10·2-17·67) | 14·84(11·01-20·74) | 0·29(1·51--0·91) |
| Guinea-Bissau | both | 125·82(91·63-177·94) | 212·9(150·76-296·27) | 69·21 | 21·36(15·72-29·86) | 17·7(12·63-24·09) | -0·62(0·42--1·65) |
| Guyana | both | 122·25(108·82-135·4) | 132·91(112·08-154·58) | 8·72 | 22·73(20·28-25·12) | 18·88(15·95-21·83) | -0·13(0·88--1·14) |
| Haiti | both | 1272·41(879·22-2007·84) | 1795·78(1231·34-2557·98) | 41·13 | 29·1(20·62-44·67) | 20·25(14-28·01) | -1·39(-0·47--2·3) |
| Honduras | both | 1063·92(834·48-1379·87) | 1950·85(1445·98-2700·29) | 83·36 | 36·44(28·31-46·32) | 27·25(20·32-37·43) | -1·19(-0·41--1·97) |
| Hungary | both | 4382·28(4103·86-4643·44) | 1244·22(1122·77-1379·62) | -71·61 | 32·93(30·8-35·02) | 8·11(7·31-9·02) | -5·82(-4·73--6·9) |
| Iceland | both | 65·34(59·6-71·58) | 51·27(44·44-58·06) | -21·52 | 23·46(21·4-25·78) | 10·94(9·52-12·31) | -1·79(-0·75--2·82) |
| India | both | 108372·95(88411·6-138422·13) | 163091·32(137572·74-190042·68) | 50·49 | 16·7(13·87-21·08) | 13·14(11·04-15·3) | -0·98(0·2--2·14) |
| Indonesia | both | 11806·65(9059·83-19469·11) | 18718·56(14136·17-26527·42) | 58·54 | 8·45(6·66-14·32) | 7·47(5·67-10·59) | -0·34(1·25--1·89) |
| Iran | both | 7155·36(3668·35-8976·06) | 6131·83(4864·75-7303·65) | -14·3 | 18·62(9·76-23·3) | 7·44(5·87-8·98) | -3·08(-1·78--4·37) |
| Iraq | both | 1490·36(1015·62-2183·42) | 1636·1(1286·08-2288·03) | 9·78 | 13·7(9·83-20·19) | 5·03(3·91-6·99) | -3·81(-2·38--5·22) |
| Ireland | both | 968·51(898·81-1043·05) | 482·24(427·72-545·7) | -50·21 | 24·68(22·9-26·67) | 7·4(6·54-8·43) | -4·32(-3·15--5·47) |
| Israel | both | 1167·91(1050·39-1296·8) | 713·7(633·46-796·39) | -38·89 | 24·13(21·68-26·69) | 6·9(6·12-7·71) | -5(-3·84--6·14) |
| Italy | both | 23911·88(22820·62-25031·8) | 10888·82(9817·09-12078·47) | -54·46 | 30·6(29·22-32·14) | 8·87(8·03-9·87) | -4·8(-3·72--5·86) |
| Jamaica | both | 142·22(123·91-161·24) | 399·72(309·06-508·61) | 181·06 | 7·83(6·76-8·89) | 13·63(10·52-17·35) | 2·68(4·16-1·21) |
| Japan | both | 27039·07(26121·56-27977·39) | 8025·12(7489·27-8646·4) | -70·32 | 17·61(17·02-18·23) | 3·59(3·37-3·84) | -6·71(-5·11--8·27) |
| Jordan | both | 100·48(71·67-137·58) | 169·33(129·24-227·37) | 68·52 | 4·48(3·08-6·09) | 2·01(1·54-2·65) | -3·45(-0·88--5·95) |
| Kazakhstan | both | 2894·27(2320-3792·43) | 1740·23(1332·02-2332·08) | -39·87 | 18·81(15·08-24·59) | 9·26(7·11-12·33) | -2·59(-1·46--3·71) |
| Kenya | both | 574·76(436·12-813·37) | 1312·77(1066·31-1634·57) | 128·4 | 4·68(3·51-6·83) | 4·23(3·41-5·4) | -0·3(1·81--2·38) |
| Kiribati | both | 10·47(7·76-13·08) | 15·5(11·28-20·37) | 48·08 | 20·05(14·62-24·84) | 16·86(12·35-21·93) | -0·51(0·56--1·57) |
| Kuwait | both | 83·25(69·99-99·05) | 137·22(112·5-165·26) | 64·83 | 5·88(5·07-6·78) | 3·33(2·76-3·9) | -1·59(0·57--3·71) |
| Kyrgyzstan | both | 380(327·81-438·12) | 263(225·61-307·53) | -30·79 | 10·71(9·23-12·42) | 4·82(4·17-5·57) | -2·65(-1·08--4·19) |
| Laos | both | 371·99(233·34-733·06) | 483·16(337·27-720·6) | 29·89 | 13·39(8·59-26·43) | 8·74(6·24-12·98) | -1·56(-0·21--2·89) |
| Latvia | both | 672·3(574·17-790·74) | 351·16(297·72-415·08) | -47·77 | 19·22(16·47-22·56) | 10·61(8·95-12·51) | -2·71(-1·57--3·83) |
| Lebanon | both | 258·87(169·3-379·97) | 423·98(301·45-570·77) | 63·78 | 9·16(5·93-13) | 5·69(4·01-7·58) | -1·82(-0·07--3·53) |
| Lesotho | both | 41·84(27·94-56·75) | 67·18(45·91-92·84) | 60·55 | 3·5(2·3-4·71) | 4·55(3·1-6·28) | 1·63(3·84--0·53) |
| Liberia | both | 180·34(136·2-226·73) | 374·77(278·15-479·73) | 107·81 | 13·81(10·46-17·42) | 12·25(9·16-15·64) | -0·62(0·65--1·88) |
| Libya | both | 199·33(139·6-267·5) | 448·37(332·53-598·63) | 124·94 | 7·66(5·27-10·41) | 7·1(5·31-9·29) | -0·31(1·38--1·97) |
| Lithuania | both | 1402·15(1241·46-1555·4) | 501·19(442·96-606·89) | -64·26 | 31·85(28·25-35·19) | 11·44(10·09-13·77) | -3·64(-2·71--4·56) |
| Luxembourg | both | 117·65(102·3-135·92) | 67·56(58·14-79·89) | -42·58 | 22·95(19·99-26·59) | 7·67(6·63-9·04) | -4·61(-3·41--5·8) |
| Macedonia | both | 279·82(216·55-347·62) | 242·66(189·23-314·88) | -13·28 | 13·56(10·61-16·74) | 8·09(6·3-10·49) | -2·28(-0·89--3·65) |
| Madagascar | both | 1149·29(912·46-1550·45) | 1966·18(1486·52-2523·78) | 71·08 | 15·01(11·98-20·15) | 12·36(9·33-15·84) | -0·73(0·53--1·98) |
| Malawi | both | 536·69(231·04-772·95) | 793·81(582·71-1007·34) | 47·91 | 9·82(4·88-13·49) | 8(5·72-10) | -1·3(0·17--2·76) |
| Malaysia | both | 2186·48(1746·89-2677·49) | 3268·75(2369·85-4542·22) | 49·5 | 17·12(13·95-21·77) | 11·27(8·18-16·2) | -1·52(-0·29--2·74) |
| Maldives | both | 37·39(19·33-76·09) | 37·78(29·6-49·8) | 1·05 | 26·39(14·45-51·16) | 9·6(7·58-12·09) | -3·95(-2·83--5·05) |
| Mali | both | 749·82(585·68-946·16) | 1151·08(809·71-1581·15) | 53·52 | 13·86(10·79-17·53) | 10·13(7·23-13·86) | -1·27(0·08--2·59) |
| Malta | both | 94·45(85·54-103·04) | 67·41(59·32-76·54) | -28·63 | 22·08(20·04-24·08) | 8·84(7·83-10·01) | -3·89(-2·71--5·05) |
| Marshall Islands | both | 4·87(3·55-6·67) | 6·42(4·1-9·49) | 32·03 | 18·37(13·68-24·82) | 13·45(8·76-19·37) | -1·24(-0·12--2·34) |
| Mauritania | both | 201·84(153·37-258·45) | 310·3(218·04-406·2) | 53·73 | 15·4(11·56-19·75) | 12(8·48-15·63) | -0·87(0·38--2·1) |
| Mauritius | both | 51·55(45·53-57·73) | 81·72(69·86-94·72) | 58·52 | 5·68(5·03-6·34) | 4·77(4·11-5·48) | -0·61(1·53--2·7) |
| Mexico | both | 8369·25(8071·41-8707·88) | 10512·08(9902·44-11132·34) | 25·6 | 13·86(13·38-14·43) | 8·46(7·97-8·95) | -2·27(-0·94--3·58) |
| Moldova | both | 298·36(244·47-353·71) | 152·42(133·22-179·22) | -48·91 | 6·34(5·19-7·48) | 2·99(2·62-3·54) | -2·75(-0·64--4·81) |
| Mongolia | both | 145·96(115·19-193·32) | 209·77(161·75-307·84) | 43·72 | 10·71(8·48-13·74) | 7·06(5·62-9·83) | -2·01(-0·56--3·45) |
| Montenegro | both | 129·33(104·03-165·52) | 112·21(88·54-150·91) | -13·24 | 19·6(15·77-25) | 12·62(9·98-16·93) | -1·82(-0·72--2·9) |
| Morocco | both | 1131·26(547·99-1516·73) | 1383·98(778·7-1901·21) | 22·34 | 6·16(2·84-8·3) | 3·94(2·2-5·38) | -1·72(0·3--3·7) |
| Mozambique | both | 1485·25(1042·55-2619·45) | 2317·03(1630·65-4291·32) | 56 | 17·74(12·81-29·87) | 14·66(10·77-25·66) | -0·74(0·43--1·89) |
| Myanmar | both | 5510·56(3342·17-11776·66) | 5180·31(3705·05-7833·6) | -5·99 | 18·02(11·2-39·01) | 10·09(7·28-15·3) | -2·03(-0·84--3·2) |
| Namibia | both | 26·9(16·39-38·74) | 32·18(20·16-45·52) | 19·66 | 3·06(1·85-4·28) | 1·91(1·18-2·68) | -1·96(0·79--4·63) |
| Nepal | both | 2746·17(2013·1-3884·95) | 3216·33(2342·5-4327·4) | 17·12 | 21·1(15·64-29·7) | 13·13(9·71-17·68) | -1·85(-0·71--2·98) |
| Netherlands | both | 4421·73(4158·72-4670·5) | 1834·1(1652·07-2058·86) | -58·52 | 23·4(22-24·79) | 6·37(5·75-7·14) | -4·57(-3·38--5·75) |
| New Zealand | both | 1038·2(949·96-1123·65) | 553·02(496·85-619·24) | -46·73 | 26·96(24·67-29·18) | 8·95(8·04-10·01) | -4·75(-3·6--5·89) |
| Nicaragua | both | 320·27(254·53-380·94) | 519·21(399·43-637·95) | 62·11 | 12·86(10·47-15·17) | 9·33(7·21-11·39) | -1·11(0·22--2·43) |
| Niger | both | 670·98(492·08-859·59) | 1477·82(1029·74-1930·67) | 120·25 | 14·91(10·91-19·25) | 13·4(9·5-17·55) | -0·6(0·62--1·81) |
| Nigeria | both | 5093·94(3265·04-8224·46) | 9520·09(5590·09-15137·82) | 86·89 | 8·77(5·62-13·97) | 7·93(4·77-12·39) | -0·47(1·12--2·04) |
| North Korea | both | 1397·62(1015·7-1863·38) | 1561·32(1109·43-2218·07) | 11·71 | 6·76(4·99-8·9) | 5·17(3·62-7·41) | -1·1(0·74--2·91) |
| Northern Mariana Islands | both | 5·15(3·44-6·91) | 3·73(2·91-5·29) | -27·59 | 14·45(10·15-18·44) | 6·51(5·13-9·23) | -3·65(-2·21--5·07) |
| Norway | both | 993·49(959·32-1027·66) | 480·64(456·85-507·52) | -51·62 | 17·2(16·61-17·78) | 6·09(5·79-6·44) | -3·95(-2·58--5·29) |
| Oman | both | 67·79(47·63-91·22) | 118·66(84·41-158·01) | 75·04 | 5·7(3·9-7·61) | 3·22(2·16-4·26) | -2·04(0·13--4·16) |
| Pakistan | both | 13887·18(9731·26-18233·31) | 26960·43(18148·55-38027·58) | 94·14 | 18·3(13·28-24·2) | 16·4(11·38-22·57) | -0·52(0·53--1·55) |
| Palestine | both | 82·88(58·13-128·59) | 175·05(128·95-260·01) | 111·22 | 6·59(4·73-10·34) | 4·89(3·62-6·93) | -1·09(0·82--2·97) |
| Panama | both | 304·42(269·67-342·45) | 351·49(308·91-397·26) | 15·46 | 16·48(14·61-18·45) | 8·83(7·75-10) | -2·22(-0·88--3·55) |
| Papua New Guinea | both | 781·83(557·18-1101·17) | 1315·28(899·53-1939·14) | 68·23 | 26·66(19·43-36·65) | 18·28(13·04-26·18) | -1·39(-0·42--2·35) |
| Paraguay | both | 223·32(176·07-284·45) | 524·5(406·24-672·82) | 134·87 | 8·1(6·45-10·39) | 8·77(6·81-11·24) | 0·82(2·36--0·68) |
| Peru | both | 1329·11(1045·96-1654·52) | 1929·13(1368·14-2386·85) | 45·14 | 7·9(6·25-9·7) | 5·98(4·25-7·36) | -1·1(0·61--2·79) |
| Philippines | both | 3454·25(2992·62-4040·58) | 8989·71(7567·78-10531·54) | 160·25 | 7·13(6·27-8·17) | 9·64(8·16-11·27) | 1·45(3·03--0·11) |
| Poland | both | 10917·69(9809·51-12051·95) | 4868·53(4398·7-5412·42) | -55·41 | 24·75(22·27-27·2) | 8·03(7·25-8·91) | -4·47(-3·32--5·59) |
| Portugal | both | 2756·16(2579·34-2938·59) | 1513·63(1340·7-1691·55) | -45·08 | 21·8(20·36-23·4) | 7·65(6·75-8·55) | -4·68(-3·49--5·86) |
| Puerto Rico | both | 677·07(608·33-752·14) | 282·7(248·72-329·58) | -58·25 | 18·46(16·56-20·48) | 5·34(4·72-6·16) | -5·66(-4·23--7·07) |
| Qatar | both | 34·34(20·64-48·93) | 120·72(63·76-364·72) | 251·57 | 13·56(7·81-18·97) | 6·06(4-12·66) | -3·84(-2·33--5·32) |
| Romania | both | 2768·94(2443·93-3141·08) | 1650·45(1478·59-1836·5) | -40·39 | 10·32(9·11-11·67) | 5·61(5·02-6·24) | -2·34(-0·78--3·88) |
| Russian Federation | both | 25120·1(22458·43-28742·35) | 21143·27(19968·72-22245·72) | -15·83 | 14(12·58-15·92) | 10·17(9·64-10·7) | -1·38(-0·18--2·57) |
| Rwanda | both | 729·28(525·54-1190·79) | 844·63(613·39-1085·45) | 15·82 | 17·27(12·68-28·45) | 10·75(7·97-13·89) | -2·24(-1·05--3·42) |
| Saint Lucia | both | 29·76(26·52-33·15) | 31·02(27·18-35·3) | 4·24 | 29·18(25·87-32·52) | 15·02(13·11-17·11) | -2·94(-1·92--3·94) |
| Saint Vincent and the Grenadines | both | 39·48(34·95-44·48) | 22·54(19·91-25·53) | -42·92 | 47·51(42·19-53·06) | 17·64(15·53-20·01) | -4·37(-3·46--5·28) |
| Samoa | both | 12·62(9·32-17·03) | 11·85(8·3-16·56) | -6·13 | 11·5(8·56-15·92) | 7·62(5·44-10·5) | -1·74(-0·24--3·22) |
| Sao Tome and Principe | both | 8·83(6·54-11·71) | 17·37(12-24·13) | 96·71 | 11·47(8·54-15·12) | 11·57(8·06-16·19) | -0·08(1·23--1·38) |
| Saudi Arabia | both | 863·22(593·63-1189·1) | 2134·51(1569·85-2855·21) | 147·27 | 10·19(6·76-13·87) | 8·57(6·4-11·09) | -0·55(0·94--2·03) |
| Senegal | both | 693·37(548·69-861·16) | 1415·2(1074·81-1834·97) | 104·11 | 15·53(12·34-19·17) | 14·54(11·09-18·73) | -0·19(1--1·36) |
| Serbia | both | 839·39(654·04-1021·87) | 857·39(625·06-1017·48) | 2·14 | 7·84(6·12-9·52) | 6·49(4·71-7·69) | -0·02(1·64--1·66) |
| Seychelles | both | 16·33(13·3-23·14) | 24·73(17·74-29·93) | 51·46 | 26·55(21·72-38·37) | 21·9(15·74-26·35) | -0·76(0·15--1·66) |
| Sierra Leone | both | 389·87(300·39-506·77) | 768·47(578·09-1012·41) | 97·11 | 15·39(11·97-20·01) | 14·81(11·15-19·41) | 0·07(1·25--1·09) |
| Singapore | both | 515·79(461·83-565·63) | 265·47(227·39-304·68) | -48·53 | 15·99(14·42-17·38) | 3·82(3·28-4·38) | -5·42(-3·87--6·94) |
| Slovakia | both | 1031·29(786·75-1360·3) | 370·63(292·88-457·25) | -64·06 | 17·45(13·19-22·84) | 4·46(3·56-5·49) | -4·88(-3·47--6·27) |
| Slovenia | both | 686·68(632·37-746·55) | 657·89(569·4-757·24) | -4·19 | 28·81(26·57-31·36) | 18·39(15·99-20·85) | -1·38(-0·45--2·3) |
| Solomon Islands | both | 44·52(31·2-64·63) | 65·43(46·33-93·26) | 46·95 | 21·28(15·29-30·01) | 14·2(10·32-19·56) | -1·51(-0·41--2·59) |
| Somalia | both | 601·41(278·23-988·69) | 1576·7(1046·04-2272·38) | 162·17 | 15·24(8·15-24·05) | 16·95(11·56-23·97) | 0·12(1·24--0·98) |
| South Africa | both | 683·99(533·86-817·56) | 686·2(561·88-926·02) | 0·32 | 2·42(1·88-2·91) | 1·33(1·09-1·76) | -2·2(0·96--5·26) |
| South Korea | both | 6546·2(5773·66-7716·07) | 3172·97(2704·17-3685·01) | -51·53 | 14·2(12·6-16·53) | 4·18(3·56-4·82) | -3·65(-2·08--5·19) |
| South Sudan | both | 396·56(191·45-651·81) | 708·94(471·83-1008·74) | 78·77 | 11·58(6·52-18·23) | 12·73(8·58-18·08) | 0·17(1·5--1·14) |
| Spain | both | 11815·22(11146·3-12466·82) | 5181·02(4639·08-5747·18) | -56·15 | 23·98(22·61-25·3) | 6·28(5·63-6·97) | -5·45(-4·24--6·63) |
| Sri Lanka | both | 1636·13(1322·82-2085·78) | 1307·57(985·75-1658·74) | -20·08 | 11·13(9·12-13·92) | 5·4(4·04-6·8) | -2·58(-0·99--4·14) |
| Sudan | both | 1803·19(939·47-2903·35) | 2284·81(1248·11-3205·42) | 26·71 | 13·74(7·09-21·07) | 8·79(4·72-12) | -1·73(-0·37--3·08) |
| Suriname | both | 39·74(34·38-45·35) | 51·9(44·24-60·15) | 30·6 | 12·36(10·73-14) | 8·61(7·36-9·95) | -1·56(-0·16--2·95) |
| Swaziland | both | 15·18(10·25-19·83) | 25·54(15·98-34·87) | 68·24 | 3·59(2·39-4·7) | 3·34(2·07-4·56) | 0·43(2·73--1·81) |
| Sweden | both | 2094·02(1964·55-2218·78) | 1375·68(1261·23-1499·21) | -34·3 | 17·04(15·97-18·14) | 8·45(7·7-9·25) | -2·7(-1·36--4·02) |
| Switzerland | both | 1438·59(1266·92-1671·55) | 929·24(822·05-1049·17) | -35·41 | 14·97(13·24-17·51) | 6·31(5·59-7·13) | -3·79(-2·47--5·1) |
| Syria | both | 1821·53(1244·15-2791·72) | 2012·04(1469·57-3109·84) | 10·46 | 25·75(16·96-39·85) | 13·45(9·81-20·71) | -3·05(-2·03--4·07) |
| Tajikistan | both | 495·04(363·79-633·35) | 478·21(359·97-698·72) | -3·4 | 13·42(9·71-17·44) | 6·35(4·82-9·09) | -3·2(-1·67--4·7) |
| Tanzania | both | 1876·08(1161·27-2638·1) | 3501·61(2475·81-4484·92) | 86·64 | 12·56(8·46-17·02) | 10·79(7·65-13·79) | -0·86(0·47--2·18) |
| Thailand | both | 8133·86(5812·84-10070·46) | 9032·54(5959·53-11117·32) | 11·05 | 16·55(11·82-20·13) | 9·89(6·64-12·11) | -2·44(-1·21--3·66) |
| The Bahamas | both | 54·22(48·13-61·21) | 78·68(67·66-91·44) | 45·11 | 26·09(23·24-29·23) | 19·22(16·57-22·2) | -1·11(-0·13--2·09) |
| The Gambia | both | 78·49(57·56-107·87) | 188·84(135·24-269·21) | 140·58 | 13·93(10·4-18·99) | 14·17(10·3-19·42) | 0·29(1·52--0·93) |
| Timor-Leste | both | 48·89(31·56-84·41) | 74·46(45·91-107·94) | 52·29 | 9·86(6·51-16·86) | 8·13(5·2-11·76) | -0·71(0·84--2·23) |
| Togo | both | 320·75(249·22-403·88) | 711·18(517·54-963·53) | 121·73 | 15·61(12·13-19·52) | 13·39(9·78-17·72) | -0·65(0·53--1·81) |
| Tonga | both | 5·66(4·51-7·13) | 5·27(4·14-6·65) | -6·98 | 8·45(6·76-10·6) | 6·02(4·76-7·58) | -1·35(0·36--3·03) |
| Trinidad and Tobago | both | 216·25(192·56-243·15) | 190·99(149·78-244·86) | -11·68 | 21·52(19·16-24·12) | 11·04(8·67-14·11) | -2·94(-1·79--4·08) |
| Tunisia | both | 350·81(250·24-509·23) | 508·28(364·03-695·98) | 44·89 | 5·82(4·1-8·5) | 4·06(2·93-5·53) | -1·55(0·48--3·54) |
| Turkey | both | 9499·49(7147·64-11961·33) | 5980·96(4979·76-8032·22) | -37·04 | 21·52(16·45-26·88) | 6·87(5·71-9·24) | -4·53(-3·27--5·78) |
| Turkmenistan | both | 269·16(194·07-348·53) | 301·26(218·07-390·34) | 11·92 | 10(7·22-12·83) | 6·39(4·69-8·21) | -1·86(-0·27--3·42) |
| Uganda | both | 984·45(752·81-1240·1) | 1977·01(1506·5-2485·86) | 100·82 | 10·46(8·13-13·18) | 9·75(7·46-12·14) | -0·54(0·85--1·91) |
| Ukraine | both | 14265·87(12134·27-17110·43) | 8629·49(7621·61-9762·24) | -39·51 | 21·97(18·67-26·36) | 13·74(12·12-15·59) | -3·05(-1·96--4·13) |
| United Arab Emirates | both | 109·4(48·24-164·52) | 673·8(368-987·74) | 515·89 | 11·85(4·19-18·56) | 8·98(3·84-15·22) | -1·13(0·29--2·53) |
| United Kingdom | both | 21716·96(20590·67-23016·05) | 7538·92(7174·27-8076·95) | -65·29 | 27·04(25·67-28·66) | 7·07(6·73-7·57) | -6·06(-4·85--7·25) |
| United States | both | 66850·84(64902·65-68952·61) | 28036·14(26696·19-29715·1) | -58·06 | 22·58(21·93-23·27) | 6·15(5·82-6·53) | -5·87(-4·56--7·17) |
| Uruguay | both | 987·35(900·47-1079·08) | 481·54(417·58-557·5) | -51·23 | 28·17(25·61-30·85) | 10·93(9·42-12·73) | -3·86(-2·81--4·9) |
| Uzbekistan | both | 1669·52(1269·12-2043·43) | 2130·7(1679·57-2613·87) | 27·62 | 10·78(8·16-13·26) | 7·32(5·83-8·9) | -1·33(0·14--2·79) |
| Vanuatu | both | 25·17(13·18-40·91) | 40·04(19·66-69·22) | 59·04 | 24·99(13·37-40·1) | 18·32(9-31·41) | -1·26(-0·29--2·22) |
| Venezuela | both | 3308·74(2960·03-3716·87) | 3476·07(2909·64-4096·44) | 5·06 | 24·39(22·07-27·16) | 11·4(9·56-13·41) | -3·2(-2·1--4·3) |
| Vietnam | both | 2144·36(1705·56-2826·26) | 3674(2583·49-4640·59) | 71·33 | 4·48(3·59-5·94) | 3·71(2·62-4·63) | -0·47(1·81--2·69) |
| Virgin Islands, U.S. | both | 14·55(12·47-16·93) | 12·47(10·41-14·68) | -14·31 | 14·78(12·69-17·16) | 8·02(6·69-9·53) | -2·64(-1·19--4·07) |
| Yemen | both | 1285·61(575·82-2180·99) | 2429·65(1340·02-3635·87) | 88·99 | 17·17(8·18-28·07) | 12·75(6·72-18·65) | -1·27(-0·1--2·43) |
| Zambia | both | 752·58(492·94-1090·65) | 1250·22(899·15-1632·25) | 66·12 | 17·26(12·01-24·07) | 12·8(9·29-16·45) | -1·59(-0·43--2·73) |
| Zimbabwe | both | 185·01(102·82-295·95) | 411·78(230·23-546·33) | 122·58 | 3·21(1·88-5·24) | 4·44(2·59-5·87) | 1·9(4·1--0·25) |

DALY, disability-adjusted life year; EAPC, estimated annual percentage change.
